# The methyltransferase SETD2 couples transcription and splicing by engaging mRNA processing factors through its SHI domain

**DOI:** 10.1038/s41467-021-21663-w

**Published:** 2021-03-04

**Authors:** Saikat Bhattacharya, Michaella J. Levy, Ning Zhang, Hua Li, Laurence Florens, Michael P. Washburn, Jerry L. Workman

**Affiliations:** 1 Stowers Institute for Medical Research, Kansas City, MO USA; 2 Department of Cancer Biology, University of Kansas Medical Center, Kansas City, KS USA

**Keywords:** Enzyme mechanisms, Histone post-translational modifications, RNA splicing, Transcription

## Abstract

Heterogeneous ribonucleoproteins (hnRNPs) are RNA binding molecules that are involved in key processes such as RNA splicing and transcription. One such hnRNP protein, hnRNP L, regulates alternative splicing (AS) by binding to pre-mRNA transcripts. However, it is unclear what factors contribute to hnRNP L-regulated AS events. Using proteomic approaches, we identified several key factors that co-purify with hnRNP L. We demonstrate that one such factor, the histone methyltransferase SETD2, specifically interacts with hnRNP L in vitro and in vivo. This interaction occurs through a previously uncharacterized domain in SETD2, the SETD2-hnRNP Interaction (SHI) domain, the deletion of which, leads to a reduced H3K36me3 deposition. Functionally, SETD2 regulates a subset of hnRNP L-targeted AS events. Our findings demonstrate that SETD2, by interacting with Pol II as well as hnRNP L, can mediate the crosstalk between the transcription and the splicing machinery.

## Abstract (web summary)

The methylation of Histone 3 at Lysine 36 (H3K36) has been implicated in the regulation of transcription and coupled processes such as mRNA splicing. Here the authors show that the histone methyltransferase SETD2 interacts with hnRNP L to mediate the crosstalk between the transcription and splicing machineries.

## Introduction

Alternative splicing (AS) of pre-mRNA is a crucial process that enables cells to synthesize different protein isoforms from the same gene^1^. It occurs by the rearrangement of intron and exon elements that are joined by protein–RNA complexes known as the spliceosome to yield mature RNAs. It is estimated that 95% of the human genes undergo AS and this gives rise to the protein diversity needed for the varied cell types and functions from a limited set of genes^2,3^. AS functions in critical biological processes including cell growth, cell death, pluripotency, cell differentiation, development, and circadian rhythms^4–6^.

It is clear now that pre-mRNA splicing is coupled to transcription. Such coupling permits the sequential recognition of emerging splicing signals by the splicing factors^7^. Two models have been proposed to explain this coupling. The “kinetic model” proposes that changes in the rate of Pol II transcription influence the splice site selection process and hence, AS^8–11^. According to the “recruitment model”, Pol II plays a central role in recruiting specific splicing regulators for co-transcriptional regulation of AS^9,11,12^.

An example of specific splicing regulators that are important in pre-mRNA processing and could be players in the “recruitment model” are the RNA-binding heterogeneous nuclear ribonucleoproteins (hnRNPs). hnRNPs bind to splice sites in the pre-mRNA and regulate splicing^13^. The role of hnRNPs in regulating gene expression is of increasing interest in disease research. The expression level of hnRNPs is altered in many types of cancer, suggesting their role in tumorigenesis^14^. In addition to cancer, many hnRNPs have also been linked to neurodegenerative diseases, such as spinal muscular atrophy, amyotrophic lateral sclerosis, Alzheimer’s disease, and frontotemporal lobe dementia^15–18^.

AS is a very context-dependent process that depends on factors such as cell type, development stage, cytokine stimulation, and DNA damage^19–24^. Furthermore, factors like the rate of transcription, specific splicing factors, histone modifications, etc. are also involved in AS regulation, which increases the complexity of the process. This being the case, it would be logical to expect that hnRNPs would bind to specific target sequences to influence AS. Proteins such as hnRNP L, bind to CA-rich regions in mRNAs^13,25,26^. Although intronic CA sequences constitute novel regulatory elements of alternative splicing, they are widespread, and hnRNPs are very abundant and ubiquitous proteins^25,27^. It is unclear what additional players determine the hnRNPs target specificity. Moreover, while a context-dependent regulation of splicing by hnRNP L has been noted^28^, it is unknown what factors determine this.

It is reasonable to speculate that the players that govern AS work in concert with one another to regulate splicing outcomes and are likely dependent on specific factors to mediate the cross talk and couplings amongst them. In support of this, it was previously shown that hnRNP L specifically interacts with the Mediator complex subunit, Med23, and regulates the splicing of a common set of genes^29^. Interaction of hnRNP L with the splicing factor, PTBP1, and their role in co-regulating splicing has also been reported^30,31^. In addition, hnRNP L has been shown to co-purify the histone methyltransferase SETD2^32^ although, the functional relevance of this interaction is not clear. Together, these findings reinforce the idea that specific interactions between hnRNPs and other proteins may not only allow the coupling of transcription and splicing but also facilitate the enrichment of hnRNPs near their target pre-mRNA transcripts.

Here, we present further evidence to support this emerging concept by showing that SETD2, which is known to travel with the elongating Pol II, interacts with hnRNP L. Further characterization of this association revealed that SETD2 interacts through the RNA-recognition motif (RRM2) of hnRNP L and a previously uncharacterized novel domain in SETD2, the SETD2-hnRNP Interaction (SHI) domain. Functionally, the deletion of the SHI domain from SETD2 leads to a reduced deposition of the histone mark H3K36me3 that is known to regulate splicing. Furthermore, the depletion of SETD2 and hnRNP L followed by RNA-seq revealed that the two proteins regulate the transcription and splicing of a common set of genes. Our findings reveal the role of SETD2 in the functional integration between the transcription and splicing machinery in mammalian cells and emphasize the direct roles of specific components in regulating AS.

## Results

### Purification of hnRNP L RRM2 reveals SETD2 as an interactor

Previously, the RRM2 domain of hnRNP L had been shown to interact with the Mediator complex subunit Med23^29^. The RRM is the most abundant RNA-binding domain in higher vertebrates^33^. Biochemical studies have revealed the versatility of the RRM’s interaction with single-stranded nucleic acids, proteins, and lipids^34,35^. We were curious whether the RRM of hnRNP L interacted with more transcription-related proteins besides Med23 to regulate splicing, especially since AS events co-regulated by Med23 and hnRNP L were a very small fraction of the hnRNP L-regulated AS events.

**Fig. 1:**
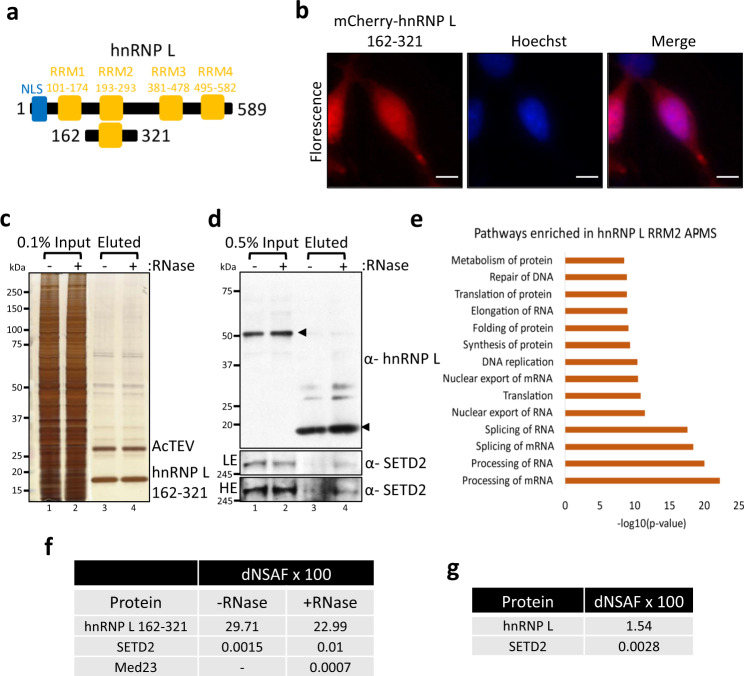
Purification of hnRNP L RRM2 reveals SETD2 as an interactor. **a** Cartoon illustrating the truncation of hnRNP L along with the known domains. RRM RNA-recognition motif, NLS nuclear localization signal. **b** Microscopy images showing localization of mCherry-hnRNP L 162–321. The scale bar is 10 µm. The experiment was repeated at least four times all yielding similar results. **c**, **d** Halo purification was performed from extracts of 293T cells expressing Halo-hnRNP L 162–321. Input and eluted samples were resolved on gel followed by silver staining or western blotting. The expected band for the target proteins are depicted by arrows. HE high exposure, LE low exposure. The experiment was repeated at least two times all yielding similar results. **e** IPA (Ingenuity Pathway Analysis) of proteins enriched in Halo-hnRNP L 162–321 purification. **f**, **g** Table showing the dNSAFs ×100 of the listed protein. dNSAF distributed normalized spectral abundance factor.

To identify putative interactor(s) of hnRNP L that might contribute to the coupling of splicing and transcription, we decided to purify the RRM2-containing hnRNP L fragment, 162–321 (Fig. 1a). hnRNP L is predicted to have a nuclear localization signal (NLS) at its N-terminal region (Fig. 1a, Supplementary Fig. 1a) and is a nuclear protein. The localization of mCherry-hnRNP L 162–321 demonstrated that it is pan-cellular, and hence its purification should reveal its nuclear interactors (Fig. 1b). Next, Halo-hnRNP L 162–321 was affinity-purified using Halo ligand-conjugated magnetic resin from 293T extracts with and without RNase treatment. Elution of the proteins purified using this technique involved cleaving the Halo-tag with TEV protease. To confirm the purification of the bait protein, silver staining as well as immunoblotting with an anti-hnRNP L antibody, that has epitope in RRM2, was performed (Fig. 1c, d). The purified complexes were subjected to multidimensional protein identification technology (MudPIT) mass spectrometry (Supplementary Data 1). The Ingenuity Pathway Analysis (IPA) of +RNase purified complexes revealed that the co-purified proteins were enriched in the pathways of RNA processing and splicing (Fig. 1e). Consistent with an earlier report^29^, Med23 was co-purified from lysate treated with RNase (Fig. 1f). Notably, there were only 25 proteins identified from both the hnRNP L with and without RNase samples that were not identified in the mock. One such protein was the histone methyltransferase SETD2 (Fig. 1f). The co-purification of SETD2 with hnRNP L 162–321 was confirmed by western blotting with an anti-SETD2 antibody (Fig. 1d).

We wanted to confirm whether the full-length hnRNP L also co-purified SETD2. For this, MudPIT of full-length Halo-hnRNP L purified from 293T extracts was performed. A total of 1236 proteins were significantly enriched over the mock (log fold change >1 and Z-statistic >2) including SETD2 (Fig. 1g, Supplementary Data 1). Previously, we and others have shown that the full-length SETD2 protein is robustly degraded by the ubiquitin-proteasome pathway^36,37^. Therefore, the low dNSAF values of SETD2 could be due to the low abundance of the endogenous SETD2 protein. The IPA showed that the purified proteins were enriched in the RNA processing pathways which is consistent with the known role of the family of hnRNP proteins (Supplementary Fig. 1b). SETD2 engages the C-terminal domain (CTD) of elongating RNA Pol II which makes it a good candidate to bridge the splicing apparatus and the transcription elongation machinery. Furthermore, much like for hnRNP L, SETD2 depletion is known to result in splicing aberrations^37–39^. Therefore, we decided to further investigate the SETD2-hnRNP L interaction.

### SETD2 can interact with hnRNP L independent of its Pol II association

It is known that hnRNPs are a class of pre-mRNA binding proteins that associate with RNA from the early stages of transcription, export to the cytoplasm, and loading onto the ribosome for translation. It is also known that SETD2 binds to elongating Pol II. Therefore, we investigated whether the SETD2-hnRNP L interaction observed was due to the presence of RNA in the lysate or the known SETD2-Pol ll interaction.

**Fig. 2:**
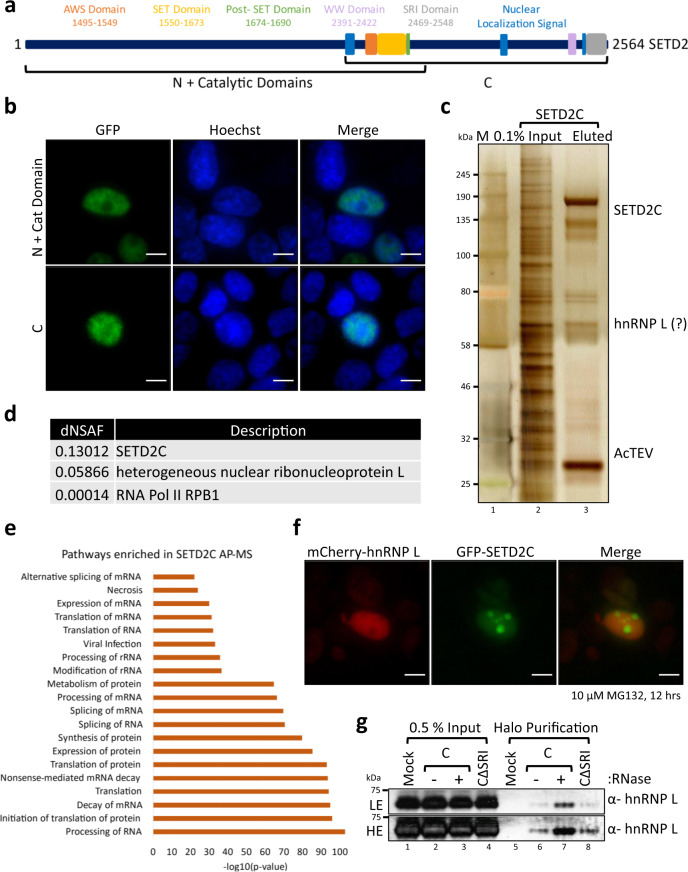
SETD2 reciprocally co-purifies hnRNP L. **a** Cartoon illustrating the overlapping segments of SETD2 used for affinity purification along with the known domains. AWS associated with SET, SET Su(var)3–9, Enhancer-of-zeste and Trithorax, SRI Set2-Rpb1 interaction. **b** Microscopy images showing localization of GFP-SETD2 fragments. The scale bar is 10 µm. The experiment was repeated at least eight times all yielding similar results. **c** Halo purification was performed from extracts of 293T cells expressing Halo-SETD2C. Input and eluted samples were resolved on a gel followed by silver staining. M—protein marker. The experiment was repeated at least 10 times all yielding similar results. **d** Table showing the dNSAFs (distributed normalized spectral abundance factor) of the listed proteins. **e** IPA (Ingenuity Pathway Analysis) of proteins enriched in Halo-SETD2C purification. AP-MS affinity purification-mass spectrometry. **f** Microscopy images showing localization of mCherry-hnRNP L and GFP-SETD2C fragment. The scale bar is 10 µm. GFP green fluorescent protein. The experiment was repeated at least four times all yielding similar results. **g** Halo purification was performed from extracts of 293T cells expressing Halo-SETD2C. Input and eluted samples were resolved on a gel and probed with an anti-hnRNP L antibody. The experiment was repeated at least two times all yielding similar results.

Previously, we established that full-length SETD2 is robustly degraded by the proteasome and its smaller fragments are much better expressed^40^. Based on this knowledge, we opted to purify two overlapping fragments of SETD2: N + catalytic domains (1–1692) and C (1404–2564 (N3) both having at least one NLS and the catalytic domains, AWS, SET, and Post-SET (Fig. 2a). Microscopy with the GFP-tagged version of the fragments revealed that both localized to the nucleus, which is consistent with our previous characterization of the SETD2 NLS (Fig. 2b)^36^. Next, Halo-SETD2 1–1692 and 1404–2564 (SETD2C) were affinity-purified from 293T extracts using Halo ligand-conjugated magnetic resin. The purified complexes were resolved on a 4–12% gradient gel, visualized by silver staining, and subjected to MudPIT (Fig. 2c). Proteomic analysis revealed a significant enrichment over mock (log fold change >1 and Z-statistic >2) of 116 proteins with the N + catalytic domains and 398 proteins with the C-terminal fragment (Supplementary Data 2). Strikingly, not only was hnRNP L co-purified with SETD2C, but it was also the most abundant protein identified in the purification after the bait (Fig. 2d, Supplementary Data 2). The reciprocal purification of Halo-SETD2 further confirmed the interaction between hnRNP L and SETD2 identified in Figs. 1f and g. Also, the RNA Pol II CTD subunit RPB1 was identified, consistent with the known role of Pol II in regulating H3K36me3 deposition (Fig. 2d). Furthermore, the IPA of proteins co-purified with both the SETD2 fragments showed enrichment of pathways belonging to RNA processing much like what was observed for hnRNP L (Fig. 2e, Supplementary Fig. 2a).

Earlier we showed that SETD2 is an aggregate-prone protein^36^. We co-expressed mCherry-hnRNP L and GFP-SETD2C in 293T cells. On MG132 treatment we saw visible puncta formed by SETD2C as expected but not by hnRNP L, suggesting that the observed interaction may not be due to protein-aggregation (Fig. 2f).

Next, Halo-FLAG-SETD2C with and without RNase treatment, and also Halo-FLAG-SETD2CΔSRI (the SRI domain is known to mediate the Set2-Pol II interaction) from 293T extracts were affinity-purified. Previously we have shown that the SETD2-Pol II interaction is not affected by RNase treatment and is abolished upon deletion of the SRI domain from SETD2^36^. Notably, the interaction with hnRNP L persisted without the Pol II interaction domain and in fact, increased upon RNase treatment (Fig. 2g and Supplementary Fig. 2b, c).

Taken together, we conclude that hnRNP L interacts with SETD2 and this interaction occurs irrespective of RNase treatment of lysate and lack of SETD2-Pol II interaction.

### Domain mapping reveals a novel SETD2-hnRNP Interaction domain in SETD2

**Fig. 3:**
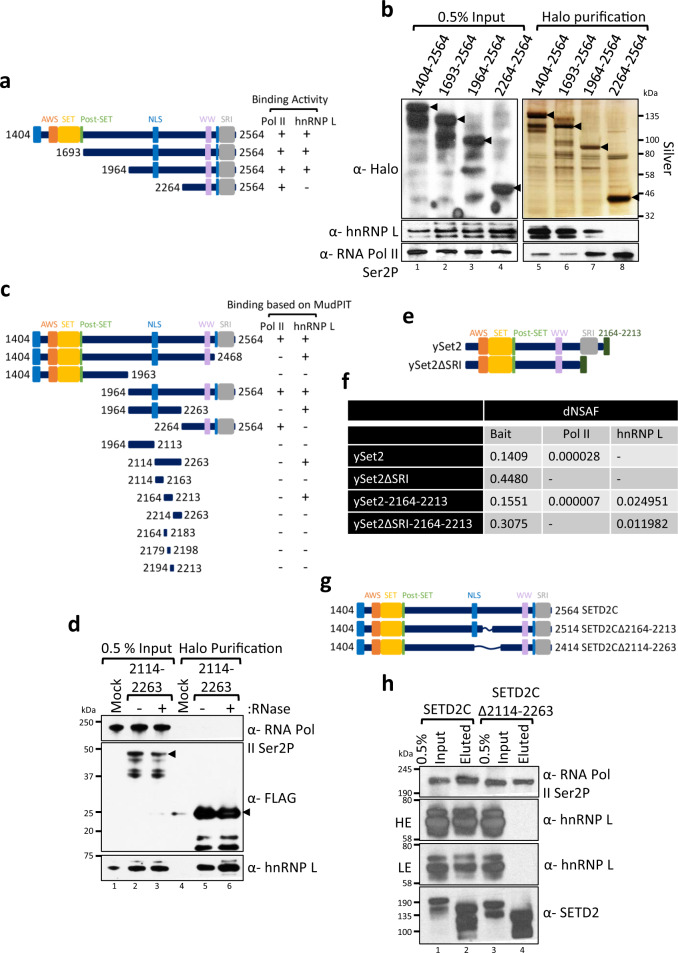
Domain mapping experiment reveals a novel SETD2-hnRNP interaction (SHI) domain in SETD2. RNase treatment was not performed for these experiments. **a**, **c**, **e**, **g** Cartoon illustrating the SETD2 and ySet2 constructs along with the known domains that were used in affinity-purifications. **b**, **d**, **h** Halo purification was performed from extracts of 293T cells expressing Halo- or Halo-FLAG-tagged proteins. Input and eluted samples were resolved on gel followed by silver staining or western blotting. The expected band for the target proteins are depicted by arrows. HE high exposure, LE low exposure, * non-specific. The experiments were repeated at least two times all yielding similar results. **f** Table showing the dNSAFs of the listed proteins post mass spectrometry analysis of purified complexes obtained by affinity purification of Halo-Set2 from 239T extracts. AWS associated with SET, SET Su(var)3–9, Enhancer-of-zeste and Trithorax, SRI Set2-Rpb1 Interaction, dNSAF distributed normalized spectral abundance factor, NLS nuclear localization signal.

We have found that the SETD2-hnRNP L interaction can occur even in the absence of the N + catalytic domains (1–1692) and the SRI domain of SETD2, indicating that the AWS, SET, Post-SET, WW, and the SRI domains are not required for the SETD2-hnRNP L interaction. We then investigated which region of SETD2 engages hnRNP L. For this, a domain mapping experiment was performed with N-terminal deletion constructs of SETD2C (Fig. 3a). The Halo-SETD2C truncations were affinity-purified from 293T extracts and binding of hnRNP L and Pol II was monitored by immunoblotting. Consistent with the known role of the SRI domain in mediating the SETD2-Pol II interaction, the fragment 2264–2564 was sufficient to co-purify Pol II (Fig. 3b). Notably, hnRNP L interaction was not observed with this fragment (Fig. 3b). This fragment was nuclear, consistent with our previous characterization of the SETD2 NLS (Supplementary Fig. 3a)^36^. Hence, the localization of this SETD2 fragment cannot explain the lack of interaction with hnRNP L, which is also nuclear. Based on these results we noted that the 1964–2263 region might be important for the SETD2-hnRNP L interaction.

To further confirm this finding, the Halo-tagged SETD2 fragment 1964–2263, as well as the adjacent fragments, 1404–1963 and 2264–2564, that contain the characterized SETD2 domains, were affinity-purified from 293T extracts. MudPIT analysis of the purified complexes revealed that the fragment 1964–2263 interacts with hnRNP L (Fig. 3c, Supplementary Fig. 3b). Remarkably, this also confirmed that this interaction occurs even without the involvement of the known SETD2 domains. To accurately define the region of interaction between SETD2 and hnRNP L, additional Halo-tagged fragments and sub-fragments of SETD2 were made as depicted in Fig. 3c and affinity-purified followed by MudPIT analysis (Supplementary Fig. 3b, Supplementary Data 3). The mass spectrometry data was confirmed by performing Halo purification of several SETD2 fragments followed by western blotting (Supplementary Fig. 3c). Using this approach, we were able to identify a 50 amino acid stretch in SETD2, 2164–2213 that co-purifies hnRNP L. To reconfirm that RNA is not required for SETD2-hnRNP L interaction, we purified Halo-SETD2 2114–2263 from 293T cell extracts with and without RNase treatment. The data corroborated our results as hnRNP L was efficiently co-purified with SETD2 even upon RNase treatment of the lysate (Fig. 3d).

The SETD2 homolog in yeast, Set2 (ySet2) contains the conserved AWS, SET, Post-SET, WW, and SRI domains (Fig. 3e). We wanted to test whether ySet2 can interact with hnRNP L when expressed in 293T cells. Halo-ySet2 and ySet2ΔSRI were expressed and purified from 293T cells and subjected to MudPIT. Interestingly, ySet2 could interact with Pol II even in human cells and this interaction was lost upon deletion of the SRI domain as expected (Fig. 3f). However, an interaction between ySet2 and hnRNP L was not observed (Fig. 3f). To test whether the 2164–2213 portion of SETD2 when added to ySet2 could result in interaction with hnRNP L, the stretch was added to ySet2, and ySet2ΔSRI followed by affinity purification and MudPIT (Fig. 3f, Supplementary Data 4). The data revealed that the addition of 2164–2213 amino acids indeed caused hnRNP L to be purified with ySet2 (Fig. 3f). These findings were also confirmed by immunoblotting with an anti-hnRNP L antibody (Supplementary Fig. 3d).

Structural modeling of 2164–2213 stretch using Robetta and iTASSER did not reveal any striking sequence characteristic with most of the predicted structure consisting of coils (Supplementary Fig. 4a, b). Based on the IUPRED2 prediction, most of the residues belonging to this region are disordered^40^. In an attempt to disrupt the SETD2-hnRNP L interaction, two truncation mutants, SETD2CΔ2164–2213, and Δ2114–2263, were made, both lacking the hnRNP L interaction domain (Fig. 3g). These mutants were affinity-purified from 293T cells using Halo ligand-conjugated resin. As anticipated, immunoblotting for RNA Pol II and anti-hnRNP L revealed that the SETD2-hnRNP L interaction was abolished without affecting the SETD2-Pol II interaction (Fig. 3h, Supplementary Fig. 4c, d). Based on these domain mapping experiments we identified a novel SETD2-hnRNP Interaction (SHI) (2114–2263) region.

### The SETD2 SHI and the hnRNP L RRM2 domains interact in vitro

**Fig. 4:**
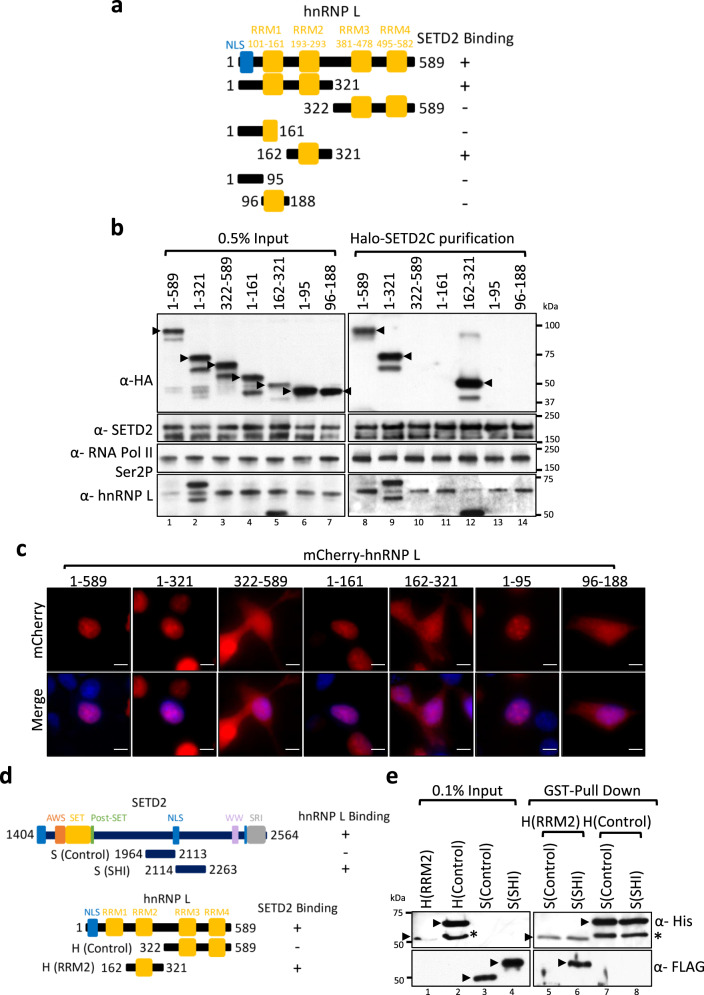
SETD2 and hnRNP L interact in vitro. **a**, **d** Cartoon illustrating the hnRNP L and SETD2 constructs along with the known domains that were used in affinity-purifications and in vitro binding. **b** Halo purification was performed from extracts of 293T cells co-expressing Halo-tagged SETD2C and mCherry-HA-hnRNP L. Input and eluted samples were resolved on gel followed by western blotting. The expected band for the target proteins are depicted by arrows. RNase treatment was not performed for these experiments. The experiment was repeated at least two times all yielding similar results. **c** Microscopy images showing the localization of mCherry-hnRNP L constructs. The scale bar is 10 µm. The experiment was repeated at least four times all yielding similar results. **e** GST pull-down was performed using recombinant proteins purified from bacteria. RNase was included in the binding assay. The input and eluted samples were resolved on gel followed by western blotting with the depicted antibodies. The experiment was repeated at least two times all yielding similar results. AWS associated with SET, SET Su(var)3–9, Enhancer-of-zeste and Trithorax, SRI Set2-Rpb1 interaction, SHI SETD2-hnRNP interaction, RRM RNA-recognition motif, NLS nuclear localization signal, GST glutathione-S-transferase.

Previously, it was reported that Med23-hnRNP L binding occurs through the RRM2 domain of hnRNP L but RRM1 also appeared to contribute to the interaction^29^. We wanted to test whether other regions of hnRNP L also interact with SETD2, besides the RRM2 domain. To address this, multiple segments of hnRNP L were tagged with mCherry-HA (Fig. 4a).

Next, the Halo-SETD2C and mCherry-HA-hnRNP L constructs were co-expressed in 293T cells and protein complexes were purified using Halo affinity purification. Immunoblotting of the purified complexes with anti-SETD2, anti-Pol II, and anti-hnRNP L antibodies demonstrated the successful purification of SETD2 and its complexes (Fig. 4b). Probing with an anti-HA antibody revealed that only RRM2-containing hnRNP L segments co-purified successfully with SETD2 (Fig. 4b). Remarkably, although the expression level of the 162–321 fragment was the lowest in lysates amongst the hnRNP L constructs as judged by input, it was co-purified most robustly with SETD2C, demonstrating a strong interaction (Fig. 4b). Also, segment 1–321, which contains both RRM1 and RRM2, was not co-purified more than RRM2 alone, and hence, it appears that the SETD2-hnRNP L interaction was not enhanced further in the presence of hnRNP L RRM1 (Fig. 4b). Microscopy revealed that the localization of the hnRNP L constructs was consistent with the NLS mapper prediction. The full-length hnRNP L (1–589) as well as the N-terminal fragments 1–321, 1–161, and 1–95 were nuclear, whereas the fragments that lack the predicted NLS, namely, 322–589, 161–321, and 96–161 were pan-cellular (Fig. 4c). Importantly, this suggested that the localization of any hnRNP L segment did not interfere with the co-purification with Halo-SETD2C, which is nuclear.

As the hnRNP L fragment 162–321 contains additional amino acids besides the RRM2, we wanted to test whether those also might be required for SETD2 binding. To test whether just the RRM2 of hnRNP L can interact with SETD2, we performed a co-IP experiment of mCherry-HA-hnRNP L 189–293 with Halo-SETD2C. The purification demonstrates that the RRM2 of hnRNP L alone is sufficient to interact with SETD2 (Supplementary Fig. 5a, b).

To further validate the interaction of the SETD2 SHI domain and the RRM2 of hnRNP L, FLAG-SETD2, and GST-His-hnRNP L fragments were recombinantly expressed and purified from bacteria and an in vitro pull-down assay was performed. For the assay, the SETD2 SHI (2114–2263) and the hnRNP L RRM2 (162–321) domains were recombinantly purified (Fig. 4d). As negative controls, a SETD2 fragment adjacent to the SHI domain (1964–2113) and an hnRNP L fragment containing the RRM3 and RRM4 (322–589) were also included in the assay (Fig. 4d). GST-His-hnRNP L segments were used as baits and FLAG-SETD2 fragments were used as preys. After the binding, the proteins were detected by immunoblotting with anti-His and anti-FLAG antibodies. The assay confirmed our affinity purification data from mammalian cell extracts that the SETD2 SHI and the hnRNP L RRM2 domains specifically interact with one another (Fig. 4e).

### SETD2 associates with splicing-related factors through the SHI domain

The function of the proteins co-purified on affinity purification of SETD2C revealed enrichment of the pathways involved in RNA processing by IPA (Fig. 2e). Of the 398 proteins that were significantly enriched over the mock (log fold change >1, Z-statistic >2), 132 were classified as RNA-binding proteins by PantherDB. The list of interactors consisted of other hnRNP proteins like A1, LL, C, U, etc. Additional pre-mRNA processing proteins like polyadenylate-binding proteins (1 and 4), serine/arginine-rich splicing factor (3 and 10), U2AF2, polypyrimidine tract binding protein 1, U1 small nuclear ribonucleoprotein A, etc. were also co-purified.

**Fig. 5:**
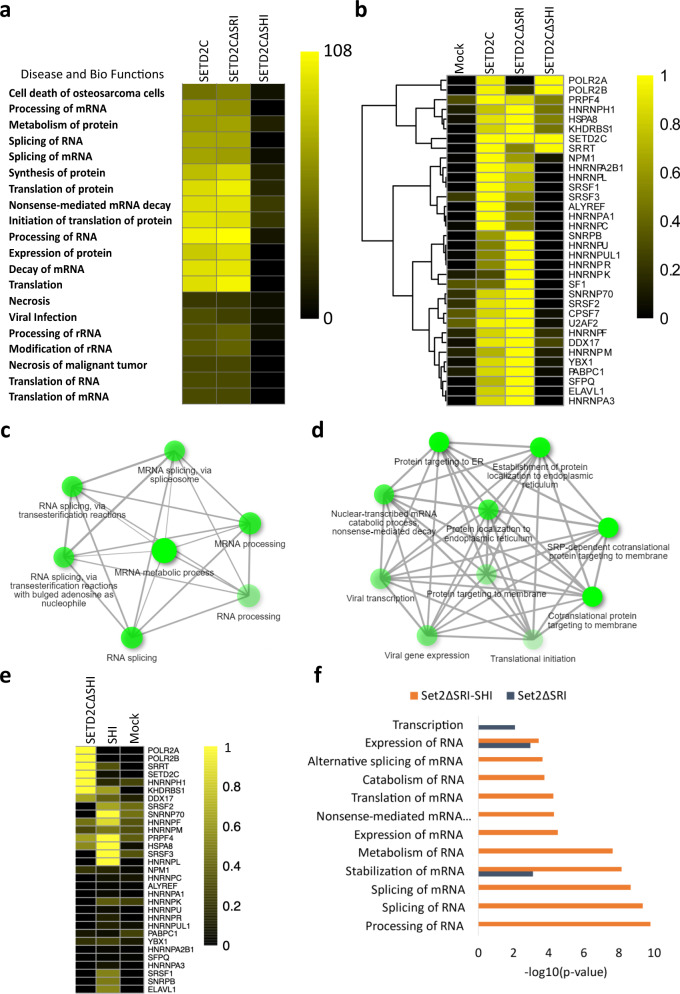
SETD2 associates with splicing-related proteins through its SHI domain. **a**, **b**, **e** Heat maps showing the enrichment of pathways in the IPA (Ingenuity Pathway Analysis) and proteins in MudPIT analysis. **c**, **d** GO-term analysis of proteins using ShinyGO (http://bioinformatics.sdstate.edu/go/) identified by MudPIT in the affinity purification of SETD2 SHI and 1964–2113 fragments. **f** Chart showing the enriched pathways in IPA of ySet2 proteins. SRI Set2-Rpb1 interaction, SHI SETD2-hnRNP interaction.

Removal of the SHI domain from SETD2 leads to the loss of interaction with hnRNP L. We wondered whether the interaction of SETD2 with other splicing-related proteins was also affected upon deletion of the SHI domain. To test this hypothesis, Halo-SETD2CΔSRI and Halo-SETD2CΔSHI, were affinity-purified and subjected to mass spectrometry (Supplementary Data 5). Function analysis by IPA of the proteins identified through MudPIT revealed that although the loss of the SRI domain did not affect the co-purification of RNA processing related proteins with SETD2, the deletion of the SHI domain led to a significant reduction in the enrichment of such protein groups (Fig. 5a). A closer inspection of the specific proteins associated with such pathways revealed that the deletion of the SHI domain not only led to the loss of the hnRNP L interaction but also resulted in the loss of interaction with other hnRNP family members like hnRNP A1 and C, that are considered part of the core hnRNP complex (Fig. 5b).

Next, a GO-term analysis of the proteins co-purified with the SETD2 SHI domain was performed. In agreement with the findings above, the proteins associated with the SHI domain were enriched in RNA processing pathways (Fig. 5c). Notably, such enrichment was not observed on the GO-term analysis of co-purified proteins with the fragment 1964–2113 which is adjacent to the SHI and does not interact with hnRNP L (Fig. 5d). Furthermore, besides hnRNP L, the SHI domain co-purified additional RNA processing proteins such as hnRNPs and SRSFs (Fig. 5e). To further validate the function of the SHI domain in mediating contact with RNA processing proteins, an IPA was performed of proteins co-purified with yeast Set2ΔSRI and chimeric fusion Set2ΔSRI-SHI. Indeed, the addition of the SHI domain to Set2 led to a pronounced enrichment in pathways related to RNA processing (Fig. 5f).

Our findings raise an interesting possibility that besides hnRNP L, SETD2 can interact with other proteins through its SHI domain. To test this possibility, we purified SETD2 from mammalian cell extracts after depleting hnRNP L. hnRNP L is a very abundant protein in cells and robustly interacts with SETD2. Hence, despite achieving a significant depletion of hnRNP L in cell extracts, a substantial amount of hnRNP L was still present in SETD2C purification (sihnRNP L) (Supplementary Fig. 6). Despite this, we found that not only did the binding of SETD2 with other splicing-related proteins persisted upon the depletion of hnRNP L, but strikingly, their binding increased relative to the purifications from extracts of scramble-siRNA (siScramble) treated cells (Supplementary Fig. 6, Supplementary Data 6). This is what would be expected to happen if the other proteins also engage with SETD2 through its SHI domain where hnRNP L binds.

Collectively, the analysis suggests that the SHI domain mediates contacts between SETD2 and proteins related to RNA processing.

### SETD2 and hnRNP L regulate the fate of a common subset of genes

**Fig. 6:**
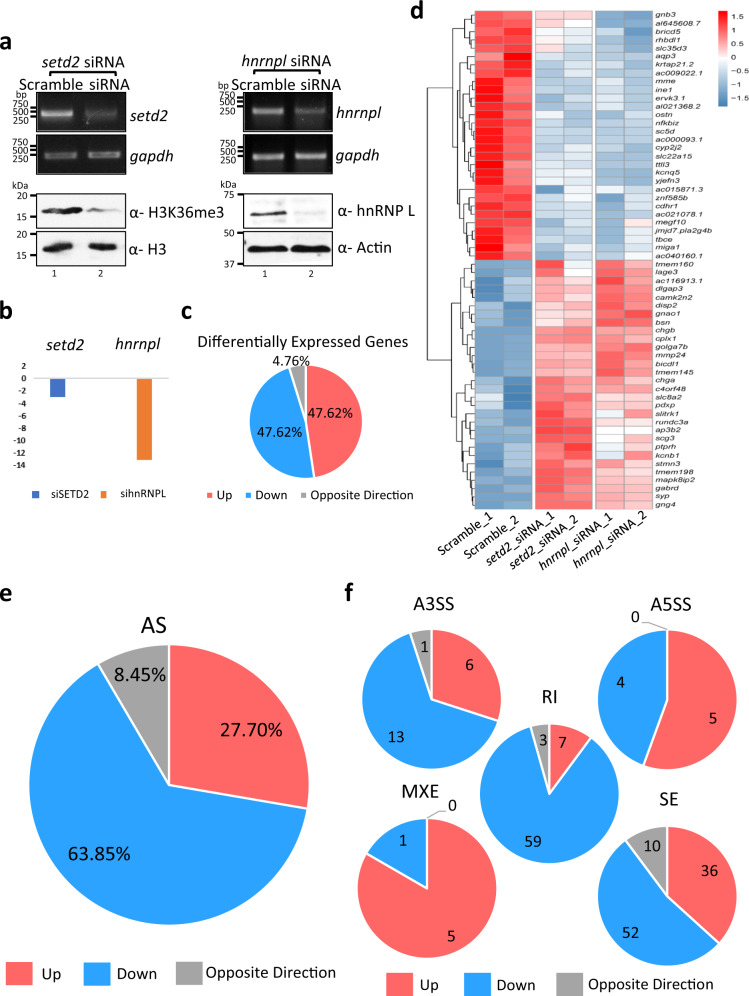
SETD2 and hnRNP L depletion affect transcription and splicing of a common subset of genes. **a** RNA was isolated from 293T cells transfected with siRNA and RTPCR was performed to check transcript levels. *gapdh* was used as a normalization control. Western blot of whole-cell lysates was performed with the depicted antibodies. The experiment was repeated at least seven times all yielding similar results. **b** Chart showing the decrease in expression of the genes depicted based on RNA-seq analysis post siRNA treatment. **c**, **e**, **f** Pie charts showing the fractions of differentially expressed genes and AS events that occur in both *setd2* and *hnrnpl* depletion. **d** Heat map showing the genes that show differential expression in both *setd2* and *hnrnpl* depleted 293T cells. AS alternative splicing, A3SS alternate 3′ splice site, A5SS alternate 5′ splice site, RI retained intron, MXE mutually exclusive exon, SE skipped exon.

The co-purification of RNA processing related proteins with SETD2 and its direct interaction with the splicing regulator hnRNP L suggests a regulatory role of SETD2 in AS. To gain insights into the functional effect of the SETD2-hnRNP L interaction in regulating the transcriptome of cells, RNA-seq was performed post depleting SETD2 and hnRNP L in 293T cells. The depletion of the target transcripts was first validated using gene-specific primers (Fig. 6a). The depletion of the targets at the protein level was confirmed by anti-H3K36me3 western blot for SETD2 (SETD2 is the sole methyltransferase for H3K36me3 deposition in human cells) and anti-hnRNP L antibody (Fig. 6a). The RNA-seq data revealed a global perturbation in terms of transcription and AS changes upon SETD2 and hnRNP L depletion (Supplementary Fig. 7). Also, the SETD2 depletion did not alter the transcript level of hnRNP L and vice versa (Fig. 6b).

We first looked deeper at the differential expression of genes induced by the depletion of SETD2 and hnRNP L. SETD2 depletion caused a significant (FDR < 0.05, fold change >1.5) upregulation of 57 genes out of which more than half showed a similar trend of increased expression upon hnRNP L depletion (Supplementary Fig. 7a, Supplementary Data 7). Also, out of the 146 genes that were significantly downregulated upon SETD2 depletion, more than a quarter of those showed a decreased expression upon hnRNP L knockdown (Supplementary Fig. 7a). Notably, 95.24% of the differentially expressed genes that are co-regulated by SETD2 and hnRNP L showed a similar trend, whereas only 4.76% showed an opposite trend of expression (Fig. 6c, d, and Supplementary Fig. 7a).

Analysis of the AS events showed a similar trend where out of the 1221 differential AS events upon SETD2 knockdown compared to a scrambled siRNA treated cells, 16% of the events showed a similar trend on hnRNP L depletion, and only 1.47% of the events showed opposite regulation (Supplementary Fig. 7b, Supplementary Data 8). Notably, of all the events that are co-regulated by SETD2 and hnRNP L, only 8.45% showed an opposite trend and the rest showed a similar trend (Fig. 6e). The overlap between SETD2-dependent and hnRNP L-dependent AS events was also reflected on analyzing for specific AS types, including alternative 3′ splice site usage (A3SS; 19 same direction, 1 opposite direction), alternative 5′ splice site usage (A5SS; 9 same direction, 0 opposite direction), intron retention (RI; 66 same direction, 3 opposite direction), mutually exclusive exon (MXE; 6 same direction, 0 opposite direction), and skipped exon (SE; 88 same direction, 10 opposite direction) (Fig. 6f). Notably, the commonly regulated AS events in the same direction outnumbered those that are oppositely regulated.

To confirm the involvement of the SETD2 SHI domain in regulating the overlapping splicing events, *setd2Δ* 293T cells, in which exon 3 of both the alleles of the endogenous *setd2* gene were disrupted using TALEN^36^, were rescued with Vector Control, SETD2 FL, and SETD2 FLΔSHI construct to test the splicing of few target genes the splicing of which was decreased upon SETD2 and hnRNP L depletion. Individual alternative splicing events were measured by quantitative PCR and represented by the ratios of the intron to an exon, or different exons. Indeed, the rescue of *setd2Δ* 293T cells with SETD2 FL led to an increase in the ratio as compared to the vector control (Supplementary Fig. 8). No change in splicing was observed upon expression of SETD2 FLΔSHI relative to the control (Supplementary Fig. 8).

These results indicate that SETD2 and hnRNP L can target partially overlapping sets of transcription and AS events.

### SETD2 and hnRNP L co-regulated genes have distinct H3K36me3 patterns

The histone mark H3K36me3 is known to regulate splicing^39,41,42^. Previously it was reported that the genes, whose splicing is co-regulated by Med23 and hnRNP L, have high H3K36me3 levels^29^. As SETD2 is the enzyme responsible for the deposition of H3K36me3, we wondered whether this mark has any correlation with the SETD2-hnRNP L co-regulated splicing events. To investigate this, ChIP-Seq of H3K36me3 was performed in 293T cells and the distribution of this mark was analyzed on genes the splicing of which is affected by SETD2 and hnRNP L depletion.

First, we examined the level of H3K36me3 on those genes the AS of which is regulated by SETD2. Clear enrichment of H3K36me3 was found on genes the splicing of which were downregulated upon SETD2 depletion, suggesting that high H3K36me3 promotes splicing (Supplementary Fig. 9a, b). This is consistent with previous reports that showed that splicing enhances recruitment of SETD2 and the deposition of the H3K36me3 mark^43,44^. Also, genes that showed a decrease in splicing upon SETD2 and hnRNP L depletion had higher H3K36me3 levels as compared to the genes that showed increased splicing or exhibited opposite trends (Supplementary Fig. 9c, d). Hence, SETD2 regulated splicing events, including those that are co-regulated by SETD2 and hnRNP L, show a correlation with the H3K36me3 level. Such a correlation was not observed when hnRNP L-regulated AS genes were analyzed (Supplementary Fig. 9e, f).

### The SHI domain is important for SETD2’s methyltransferase activity

**Fig. 7:**
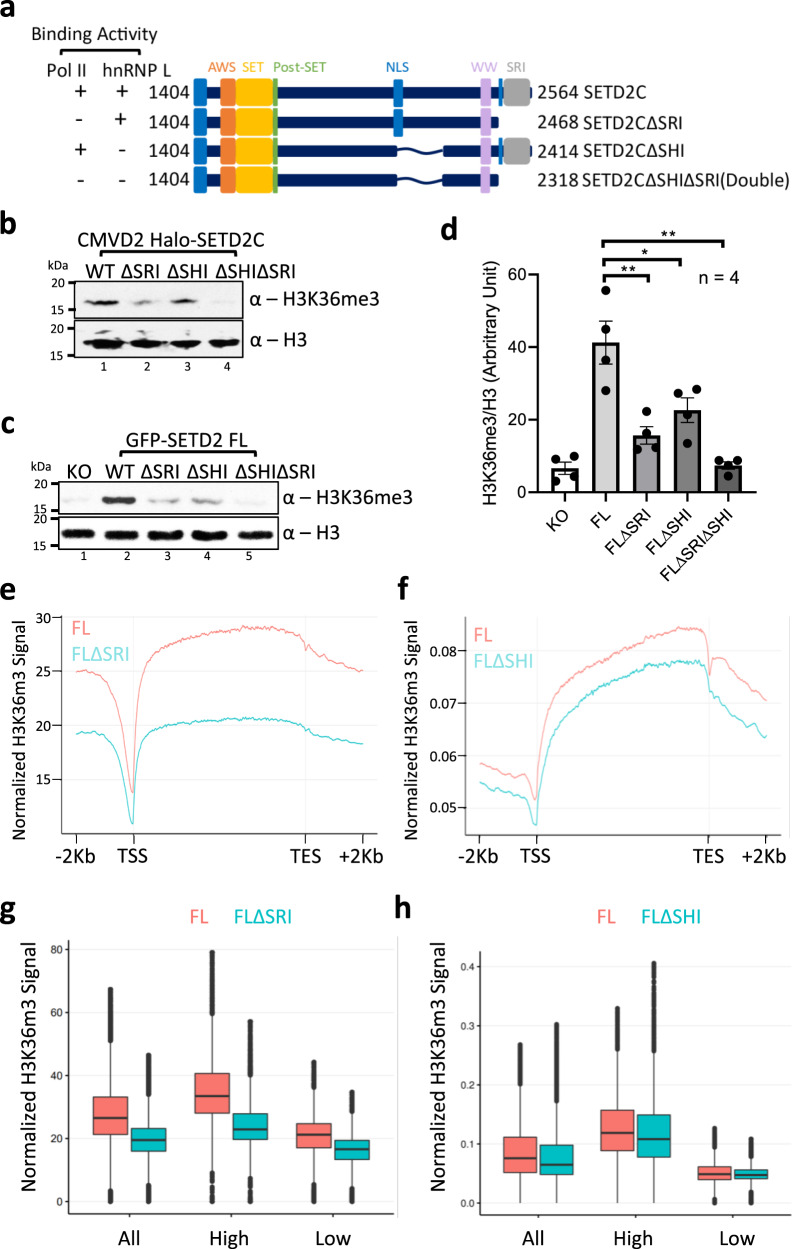
The SHI domain regulates SETD2 activity. **a** Cartoon illustrating the SETD2 constructs along with their known domains that were used to compare the ability to deposit H3K36me3 in KO cells. **b**, **c** Western blot with the depicted antibodies of whole-cell lysates of KO cells expressing SETD2 mutants. The experiment was repeated at least five times all yielding similar results. **d** Bar graph showing H3 normalized H3K36me3 signal intensity of data depicted in (**c**). *n* = 4 independent biological samples examined in four independent experiments. Unpaired *t* test (two-tailed) was performed. *p*-value <0.05 (FLΔSRI vs FL = 0.0071; FLΔSHI vs FL = 0.0344; FLΔSRIΔSHI vs FL = 0.0013) was considered significant. Data are presented as mean values with standard error of mean. **e**, **f**, **g**, **h** Metagene plot and boxplot depicting the distribution of H3K36me3 upon expression of SETD2 FL, FLΔSRI, and FLΔSHI in *setd2Δ* 293T cells. For each sample *n* = 2 independent biological samples examined in the same sequencing run. In the boxplots, the black line inside the box shows the median. The box bottom and top border correspond to 25th and 75th percentiles (Q1 and Q3, respectively). The whiskers represent ranges from Q1 − 1.5 * IQR to Q3 + 1.5 * IQR where IQR stands for interquartile range (Q3–Q1). Data points outside the whiskers could be outliers and are marked as black dots. AWS associated with SET, SET Su(var)3–9, Enhancer-of-zeste and Trithorax, SRI Set2-Rpb1 interaction, SHI SETD2-hnRNP interaction, NLS nuclear localization signal, KO knock out (*setd2Δ* 293T cells), TSS transcription start site, TES transcription end site.

It is well-established that the Set2/SETD2 SRI domain regulates its methyltransferase activity. This prompted us to investigate whether the SHI domain also governs SETD2 activity similar to the SRI domain. Consequently, Halo-SETD2C mutants having a deletion of SRI or SHI or both were made (Fig. 7a). ﻿To check the activity of the exogenously introduced SETD2 constructs, *setd2Δ* 293T (KO) cells were used. Halo-SETD2C constructs with CMVD2 promoter were introduced in the KO cells and the H3K36me3 levels were analyzed 72 h post-transfection. As expected, the deletion of the SRI domain reduced the SETD2C activity (Fig. 7b). Strikingly, the deletion of the SHI domain also led to a decrease in H3K36me3 deposition, although, the decrease was not as severe as that observed upon SRI deletion (Fig. 7b). To test that these observations also hold true for the full-length SETD2 protein, similar mutants were made in the full-length protein and GFP-SETD2 constructs were introduced in the KO cells. The H3K36me3 levels were analyzed 72 h post-transfection. Despite comparable expression levels of the constructs, a clear difference was observed in the H3K36me3 level between the cells rescued with WT SETD2 and the SETD2 mutants lacking the SRI and the SHI domains (Fig. 7c, d, and Supplementary Fig. 10a). Also, the double mutant lacking both the SHI and SRI domains almost completely lost activity suggesting that these domains are global regulators of SETD2 activity.

Next, we performed spike-in normalized H3K36me3 ChIP-Seq of *setd2Δ* 293T cells expressing SETD2 FL, FLΔSRI, and FLΔSHI. ChIP-Seq analysis corroborated our western blot results that there is a global decrease in H3K36me3 deposition in the cells rescued with SETD2 mutants as compared to SETD2 FL (Fig. 7e, f). Furthermore, a closer inspection revealed that the loss occurs from both high and low expressed genes, consistent with the idea that both Pol II and hnRNP L are global regulators of SETD2’s in vivo activity (Fig. 7g, h) (see the “Discussion” section). Also, the decrease in H3K36me3 does not occur specifically from those genes the splicing of which is co-regulated by SETD2 and hnRNP L as H3K36me3 pattern was similar in SETD2 FLΔSHI expressing cells to that observed in SETD2 FL expressing and WT cells (Supplementary Fig. 10b).

We conclude that besides the SRI domain, SETD2 activity is also regulated by its SHI domain. The additive effect of the loss of SHI and SRI further suggests that these domains independently impact SETD2 activity.

## Discussion

Our data provide evidence to support the recruitment model for the coupling of splicing and transcription. H3K36me3 is known to regulate splicing^39,41,42^. Our work reveals that in addition to regulating splicing through its catalytic activity by deposition of the H3K36me3 mark, SETD2 can regulate AS by directly interacting with the pre-mRNA processing proteins. Co-transcriptional splicing requires the splicing factors to engage pre-mRNA while it is still being transcribed. The ability of SETD2 to bind to the elongating Pol II as well as the splicing factors makes it an ideal candidate to facilitate such a temporal process.

**Fig. 8:**
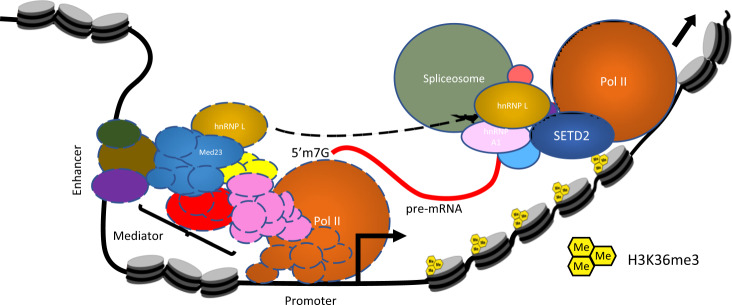
Pre-mRNA processing and transcription are coupled. A cartoon speculating a possible mechanism of cross talk between hnRNP L and the transcription machinery. See text for details.

Earlier studies aimed to find the RNA-binding motif of hnRNP L revealed that it binds to CA-rich regions, which are widespread in mRNAs. This might enable a splicing factor to bind to a variety of targets, hence, reducing the requirement for cells to create diversity in splicing factors with different target specificity but redundant function. However, this generates a need for a guiding mechanism to ensure correct pre-mRNA processing. Transcription factors and epigenetic regulators work in a context-dependent and cell line-specific manner. Hence, it is reasonable that the splicing factors will leverage this attribute of transcription factors and epigenetic regulators by interacting with them to govern AS. Maybe proteins like Med23 and SETD2 guide hnRNP L to engage with the correct target pre-mRNA. Med23 has been shown to recruit hnRNP L to the promoter of genes^29^. However, how hnRNP L might be recruited to pre-mRNA transcripts following its initial recruitment to the promoter by Med23 is not clear. As the mediator complex is not known to travel with the elongating Pol II, the finding raises the intriguing question of how hnRNP L exerts its effect on alternative splicing of pre-mRNA that is far downstream from the promoter region^45^. One mechanism that can be envisioned is that Med23 recruits hnRNP L to the target genes during transcription initiation and subsequently hands it over to other factors (Fig. 8). SETD2 is well suited to be such a factor as during the elongation phase, SETD2 hitchhikes by interacting with Pol II through its SRI domain. At this stage, hnRNP L binding to the SHI domain of SETD2 will bring it in close proximity to the pre-mRNA molecule being transcribed (Fig. 8). As more of the pre-mRNA molecule emerges from the transcription bubble and the hnRNP L binding sequence becomes available, hnRNP L may engage the pre-mRNA.

Several years of research have tried to elucidate how hnRNPs function, however, the details are still scarce. hnRNP L’s mechanism of action is not clear and also, it has been observed to have a context-dependent nature^28^. hnRNP proteins A1, A2/B1, B2, C1, and C2 have a somewhat indiscriminate association with nascent transcripts and are termed the “core” hnRNP proteins^46^. hnRNP L has been shown to bind its target pre-mRNA and recruit hnRNP A1^47^. hnRNP A1 in turn is known to recruit ﻿U2 small nuclear RNA auxiliary factor 2 (U2AF2) which is a critical part of early steps in spliceosome assembly^48^. Notably, in SETD2C purifications, hnRNP A1 was the second most abundant protein after the bait, and U2AF2 was also identified in MudPIT (Supplementary Data 2). Importantly, both of these interactions were lost along with hnRNP L upon deletion of the SHI domain but not upon the deletion of the SRI domain (Fig. 5b). Notably, the purification of SETD2 from extracts depleted of hnRNP L revealed enrichment of hnRNP A1 (Supplementary Fig. 6). On the other hand, the enrichment of U2AF2 was reduced (Supplementary Fig. 6). Possibly, SETD2 can recruit spliceosomes by more than one mechanism. SETD2 by interacting with hnRNP L might result in the recruitment of proteins such as U2AF2. SETD2 might also engage the core hnRNP proteins which in turn can bring other splicing factors to form the spliceosome to dictate AS outcomes.

Our detailed characterization of the SETD2-hnRNP L interaction revealed that only the RRM2 of hnRNP L binds to SETD2. Notably, the same region also binds to Med23^29^. A sequence alignment of the four hnRNP L RRMs did not reveal any striking pattern that would explain the specificity of RRM2, and not the other RRMs, in engaging SETD2 and hnRNP L (Supplementary Fig. 5c). More structural insights are likely needed to understand the reason behind such specificity.

It is also noticeable that SETD2 regulates some, but, not all the hnRNP L targets and vice versa much like what was observed for Med23 and hnRNP L^29^. This could be due to the complexity of hnRNP L’s mode of action in mRNA processing such as context-dependency and regulation involving other hnRNP proteins and transcription factors. On the other hand, IPA of the co-purified proteins with the SETD2 N-terminus revealed enrichment of splicing-related pathways (Supplementary Fig. 1b). Also, purification of SETD2 from cell extracts depleted of hnRNP L revealed enrichment of other proteins involved in pre-mRNA splicing (Supplementary Fig. 6). It is possible that the SETD2 N-terminus region and the SHI domain might be involved in regulating pre-mRNA processing that is independent of the hnRNP L interaction. The H3K36me3 modification that is deposited by SETD2, is known to recruit splicing factors like PTBP1 by acting as a docking site for MRG15^49^. Notably, hnRNP L has been reported to interact with PTBP1^30^. It would be interesting to address in the future if and to what extent a cross talk exists between these different regulatory factors.

We recently showed that the N-terminal segment that is absent in ySet2 regulates SETD2 half-life^40,50^. The C-terminal segment of SETD2 (1404–2564) shares similarities with ySet2 and has conserved domains such as AWS, SET, Post-SET, WW, and SRI. Remarkably, in addition to these, we found a novel domain in the SETD2 C-terminal segment, the SHI domain, that mediates SETD2-hnRNP L interaction. The inability of ySet2 to engage with hnRNP L in 293T cells demonstrates that hnRNP L is a mammalian specific interactor of Set2. Splicing is largely a higher eukaryotic specific event because most yeast genes do not have intron(s). With the development of alternative splicing in vertebrates, possibly the SHI domain co-evolved in the mammalian Set2 (SETD2) to facilitate interaction with the spliceosome. Previously it has been demonstrated that Set2-Pol II interaction through the SRI domain is required for the activation of Set2 which likely occurs by the alleviation of inhibition imposed by its autoinhibitory domain (AID)^51^. It has been speculated that SETD2 also has an AID and our recent work supports the idea that the interaction with Pol II is required for SETD2’s activation and not for chromatin recruitment^40,51^. Our data strikingly reveals that in mammalian cells, besides the SRI domain, the SHI domain also regulates SETD2 activity. This is again consistent with the need for AS in mammalian cells which is absent in yeast. In fact, unlike yeast, which does not have an hnRNP L homolog, flies do have a homolog called Smooth. It will be interesting to examine in future studies whether the *Drosophila* Set2 can interact with Smooth and whether this plays a similar role in flies that SETD2-hnRNP interaction plays in mammals.

## Methods

### Plasmids

hnRNP L and SETD2 human ORF were procured from Promega. Deletion mutants of hnRNP L and SETD2 were constructed by PCR (Phusion polymerase, NEB) using full-length hnRNP L and SETD2, respectively, as a template and individual fragments were cloned. All constructs generated were confirmed by sequencing. pCDNA3-ySet2 were procured from Addgene. siRNA for *setd2* and *hnrnpl* as well as scramble-siRNA sequence were procured from Dharmacon.

### Cell line maintenance and drug treatment

293T cells were procured from ATCC and maintained in DMEM supplemented with 10% FBS and 2 mM L-glutamine at 37 °C with 5% CO_2_. MG132 (Sigma) was added at a final concentration of 10 μM for 12 h. Transfections of plasmids were performed using Fugene HD (Promega) at and that of siRNAs was performed using Lipofectamine RNAi Max (Thermosfisher) at 40% cell confluency.

### Affinity purification

293T cells expressing the protein of interest were harvested in 1× PBS and collected by centrifugation. The cells were lysed by resuspending in lysis buffer (50 mM Tris, pH 7.5, 150 mM NaCl, 1% Triton X-100, 0.1% Na-deoxycholate, and a protease inhibitor cocktail). The lysed cells were centrifuged at 16,000 × *g* for 20 min. The supernatant was collected and diluted 1:3 by adding dilution buffer (1× PBS, pH 7.5 with 1 mM DTT and 0.005% NP40). The diluted lysate was added to pre-equilibrated Magne® HaloTag® Beads (Promega, G7282) and incubated overnight on a rotator at 4 °C. The beads were then washed with wash buffer (50 mM Tris-HCL, pH 7.5, 300 mM NaCl, 0.005% NP40, and 1 mM DTT. AcTEV (ThermoFisher, 12575015) protease was used for elution.

### Mass spectrometry analysis

TCA precipitated protein samples were analyzed independently by Multidimensional Protein Identification Technology (MudPIT)^52,53^. Briefly, precipitated protein samples were resuspended in 100 mM Tris pH 8.5, 8 M urea to denature the proteins. Proteins were reduced and alkylated prior to digestion with recombinant LysC (Promega) and trypsin (Promega). Reactions were quenched by the addition of formic acid (FA) to a final concentration of 5%. Peptide samples were pressure-loaded onto 100 µm fused silica microcapillary columns packed first with 9 cm of reverse phase material (Aqua; Phenomenex), followed by 3 cm of 5-μm Strong Cation Exchange material (Luna; Phenomenex), followed by 1 cm of 5-μm C18 RP. The loaded microcapillary columns were placed in-line with a 1260 Quartenary HPLC (Agilent). The application of a 2.5 kV distal voltage electrosprayed the eluting peptides directly into LTQ linear ion trap mass spectrometers (Thermo Scientific) equipped with a custom-made nano-LC electrospray ionization source. Full MS spectra were recorded on the eluting peptides over a 400–1600 *m*/*z* range, followed by fragmentation in the ion trap (at 35% collision energy) on the first to fifth most intense ions selected from the full MS spectrum. Dynamic exclusion was enabled for 120 s^54^. Mass spectrometer scan functions and HPLC solvent gradients were controlled by the XCalibur data system (Thermo Scientific).

RAW files were extracted into .ms2 file format^55^ using RawDistiller v. 1.0, in-house developed software^56^. RawDistiller D(g, 6) settings were used to abstract MS1 scan profiles by Gaussian fitting and to implement dynamic offline lock mass using six background polydimethylcyclosiloxane ions as internal calibrants^56^. MS/MS spectra were first searched using ProLuCID^57^ with a 500 ppm mass tolerance for peptide and fragment ions. Trypsin specificity was imposed on both ends of candidate peptides during the search against a protein database combining 44,080 human proteins (NCBI 2019-11-03 release), as well as 426 common contaminants such as human keratins, IgGs, and proteolytic enzymes. To estimate false discovery rates (FDR), each protein sequence was randomized (keeping the same amino acid composition and length) and the resulting “shuffled” sequences were added to the database, for a total search space of 89,038 amino acid sequences. A mass of 57.0125 Da was added as a static modification to cysteine residues and 15.9949 Da was differentially added to methionine residues.

DTASelect v.1.9^58^ was used to select and sort peptide/spectrum matches (PSMs) passing the following criteria set: PSMs were only retained if they had a DeltCn of at least 0.08; minimum XCorr values of 2.1 for singly-, 2.7 for doubly-, and 3.2 for triply-charged spectra; peptides had to be at least 7 amino acids long. Results from each sample were merged and compared using CONTRAST^58^. Combining all replicates, proteins had to be detected by at least 2 peptides and/or 2 spectral counts. Proteins that were subsets of others were removed using the parsimony option in DTASelect on the proteins detected after merging all runs. Proteins that were identified by the same set of peptides (including at least one peptide unique to such protein group to distinguish between isoforms) were grouped together, and one accession number was arbitrarily considered as representative of each protein group.

NSAF7^59^ was used to create the final reports on all detected peptides and non-redundant proteins identified across the different runs. Spectral and peptide level FDRs were, on average, 0.52 ± 0.41% and 0.39 ± 0.1%, respectively. QPROT^60^ was used to calculate a log fold change and Z-score for the samples compared to the mock control.

For instances where there was more than one replicate analyzed by MudPIT, proteins with log fold change >1 and Z-score >2 were further analyzed in IPA (Qiagen) to determine pathways enriched by the bait proteins. For proteins with only one replicate, a ratio was calculated of dNSAF values between sample and mock. For those to be further analyzed in IPA, the dNSAF ratio had to be >2 compared to mock. Pathways were considered significantly enriched with *p*-value <0.05 (−log10(*p*-value) >1.3).

### Recombinant protein purification

FLAG-SETD2 and His-hnRNP L coding sequences were cloned into pGEx4T vector backbone and transformed into Rosetta 2 (DE3) pLysS. A single colony was inoculated into LB media containing 100 µg/ml ampicillin and 25 µg/ml chloramphenicol and grown at 37 °C. After the OD_600_ reached 0.6, the cultures were induced with 0.1 mM IPTG and grown O/N at 16 °C in a shaker. Next, the cultures were pelleted down, flash-frozen in liquid nitrogen, and stored at −80 °C. Next, the pellets were thawed on ice and resuspended in lysis buffer (50 mM Tris-HCl, pH 8.0, 200 mM NaCl, 0.05% Triton X-100). The cells were then sonicated for lysis and centrifuged at 15,000 × *g* for 30 min at 4 °C to separate the soluble and insoluble fractions. Next, binding was performed between the soluble fraction (supernatant) and glutathione-conjugated magnetic beads (Promega) pre-equilibrated with lysis buffer. After binding, the beads were washed with lysis buffer and eluted with either glutathione or AcTEV protease.

### Isolation of total RNA and PCR

Total RNA was extracted from cells as per the manufacturer’s (Qiagen) instructions. It was further treated with DNaseI (NEB) for 30 min at 72 °C to degrade any possible DNA contamination. RNA (2 μg) was subjected to reverse transcription using QScript cDNA synthesis mix according to the manufacturer’s instructions. cDNAs were then amplified with the corresponding gene-specific primer sets. For RTPCR, PCR was conducted for 24 cycles using the condition of 30 s at 94 °C, 30 s at 60 °C, and 30 s at 72 °C. The PCR products were analyzed on 1% agarose gels containing 0.5 μg/ml ethidium bromide. The sequence of oligos is in Supplementary Table 1.

### Histone isolation and immunoblot analysis

First, nuclei were isolated from cells. For this, the cell pellet was resuspended in 0.1 ml PBS in a microcentrifuge tube. To this suspension, 0.9 ml lysis solution (250 mM sucrose, 50 mM Tris–Cl pH 7.5, 25 mM KCl, 5 mM MgCl_2_, 0.2 mM PMSF, 50 mM NaHSO_3_, 45 mM sodium butyrate, 10 mM β-ME, and 0.2% v/v Triton X-100) was added. Tube was inverted several times and centrifuged for 15 min at 800 × *g*, 4 °C. The nuclear pellet obtained was subjected to histone extraction by acid extraction method by adding 0.3 ml of 0.2 M H_2_SO_4_. The tubes were vortexed thoroughly with intermittent incubation on ice. The tubes were then centrifuged at 13,000 × *g*, 4 °C for 30 min. The supernatant was transferred to a fresh tube without disturbing the pellet. The proteins in the supernatant were precipitated by adding 4 volumes of acetone and stored overnight at −20 °C. The tubes were then centrifuged at 13,000 × *g*, 4 °C for 10 min. The pellet was washed once in chilled acidified acetone (0.05 M HCl in 100% acetone) and once in chilled 100% acetone. Protein pellet was dried in a vacuum centrifuge for 15 min. The pellet was resuspended in 0.1% β-ME at −20 °C. For immunoblotting, histones were resolved on 15% SDS–polyacrylamide gel, transferred to PVDF membrane, and probed with antibodies. Signals were detected by using the ECL plus detection kit (ThermoFisher).

### Antibodies

hnRNP L (CST 37562, dilution 1:3000), FLAG (Sigma-Aldrich A8592, dilution 1:10,000), Pol II (Abcam ab5095, dilution 1:5000), Halo (Promega G9211, dilution 1:10,000), SETD2 (Abclonal A3194, dilution 1:6000 and Aviva OAEB00589, dilution 1:3000), HA (Sigma 04-902, dilution 1:10,000), His (Abcam ab18184, dilution 1:3000), H3K36me3 (CST 4909S, dilution 1:1000), H3 (CST 9715S, dilution 1:3000), β-actin (Abcam ab8224, dilution 1:2500). The scanned films from western blotting experiments are provided as Source data file.

### ChIP

Cells were cross-linked by 1% formaldehyde for 10 min, and then quenched in 125 mM glycine for 5 min. After washing with cold 1× PBS thrice, cells were harvested by scraping and pelleted down by centrifugation. The cell pellet was resuspended in swelling buffer (25 mM HEPES pH 8, 1.5 mM MgCl_2_, 10 mM KCl, 0.1% NP40, 1 mM DTT, protease inhibitor cocktail), kept in ice for 10 min and then dounced. The nuclear pellet was obtained by centrifugation and resuspended in sonication buffer (50 mM HEPES pH 8, 140 mM NaCl, 1 mM EDTA, 1% Triton X-100, 0.1% Na-deoxycholate, 0.1% SDS, protease inhibitor cocktail), followed by sonication on ice for 12 cycles (30% amplitude, 10-s on/60-s off) using a Branson Sonicator. For spike-in normalization, the spike-in chromatin and antibody were added to the reaction as per the manufacturer’s recommendation (Active Motif). The chromatin was incubated with antibodies at 4 °C overnight and then added to 30 μl of protein G-Dyna beads (ThermoFisher Scientific) for an additional 2 h with constant rotation. The beads were extensively washed, and bound DNA was eluted with elution buffer (50 mM Tris-HCl pH 8, 5 mM EDTA, 50 mM NaCl, 1% SDS) and reverse-cross-linked at 65 °C overnight. DNAs were purified using QIAquick PCR purification kit (Qiagen) after treatment of proteinase K and RNase A.

### High-throughput sequencing

Sequencing libraries were prepared using High Throughput Library Prep Kit (KAPA Biosystems) following the manufacturer’s instructions. The library was sequenced on an Illumina HiSeq platform with paired reads of 75 bp for RNA-seq and single reads of 50 bp for ChIP-seq.

### ChIP-seq analysis

Raw reads were demultiplexed into FASTQ format allowing up to one mismatch using Illumina bcl2fastq2 v2.18. Reads were aligned to the human genome (hg38) using Bowtie2 (version 2.3.4.1) with default parameters^61^. For samples with fly spike-in, reads were first mapped to the *Drosophila melanogaster* genome (dm6), and unmapped reads were then aligned to the human genome (hg38). Reads per million (RPM) normalized bigWig tracks were generated by extending reads to 150 bp. For spike-in ChIP-seq data, we also generated spike-in normalized bigWig tracks (RPM normalization factor = 1E6/number of reads aligned to hg38, and spike-in normalization factor = 1E6/number of reads aligned to dm6).

### Metagene plots

4533 Protein-coding genes (Ensembl 96 release) were selected with length ≥600 bp and no other genes within −2 Kb TSS and +2 Kb TES regions. Metagene regions were from −2 Kb TSS to +2 Kb TES. In addition, 2 Kb upstream TSS and downstream TES regions are grouped into 100 bins (20 bp per bin), respectively. The gene body region is grouped into 300 bins (at least 2 bp per bin since the minimum gene length is 600 bp). In total, each gene is grouped into 500 bins. The average normalized (RPM or spike-in) H3K36me3 signals in each bin were plotted using R package EnrichedHeatmap^62^.

### RNA-seq analysis

Raw reads were demultiplexed into FASTQ format allowing up to one mismatch using Illumina bcl2fastq2 v2.18. Reads were aligned to the human genome (hg38 and Ensembl 96 gene models) using STAR (version STAR_2.6.1c)^63^. TPM expression values were generated using RSEM (version v1.3.0). edgeR (version 3.24.3 with R 3.5.2) was applied to perform differential expression analysis, using only protein-coding and lncRNA genes^64^. To perform differential splicing analysis, we used rMATs (version 4.0.2) with default parameters starting from FASTQ files^65^. FDR cutoff of 0.05 was used to determine statistical significance.

### Reporting summary

Further information on research design is available in the Nature Research Reporting Summary linked to this article.

## Supplementary information

Supplementary Information

Peer Review File

Description of Additional Supplementary Files

Supplementary Data 1-8

Reporting Summary

## Data Availability

All relevant data are available from the corresponding author upon reasonable request. The data sets are available in the Gene Expression Omnibus (GEO) database under the accession number GSE151296. The mass spectrometry proteomics data have been deposited to the ProteomeXchange Consortium via the PRIDE partner repository with the dataset identifier PXD019376. In addition, the SETD2C truncation variants for Fig. 3 have been deposited with the dataset identifier PXD019538. Also, the data for Supplementary Fig. 6b have been deposited with the dataset identifier PXD022946. Original data underlying this manuscript can also be accessed from the Stowers Original Data Repository at http://www.stowers.org/research/publications/libpb-1582. Source data are provided with this paper.

## Source data

Source Data
