# Supplementary material for: The methyltransferase SETD2 couples transcription and splicing by engaging mRNA processing factors through its SHI domain
Source: Nat Commun. 2021 Mar 4;12:1443. doi: 10.1038/s41467-021-21663-w (PMC7933334; doi:10.1038/s41467-021-21663-w)
Supplement: Supplementary file 1 — Supplementary Information [file 41467_2021_21663_MOESM1_ESM.pdf]

The methyltransferase SETD2 couples transcription and splicing by engaging mRNA processing factors through its SHI domain

a

Predicted monopartite NLS		
Position	Sequence	Score
6	DRGPLKKRRQEIE	6
7	RGPLKKRRQEI	8
54	PLKKRRQEIE	10

b

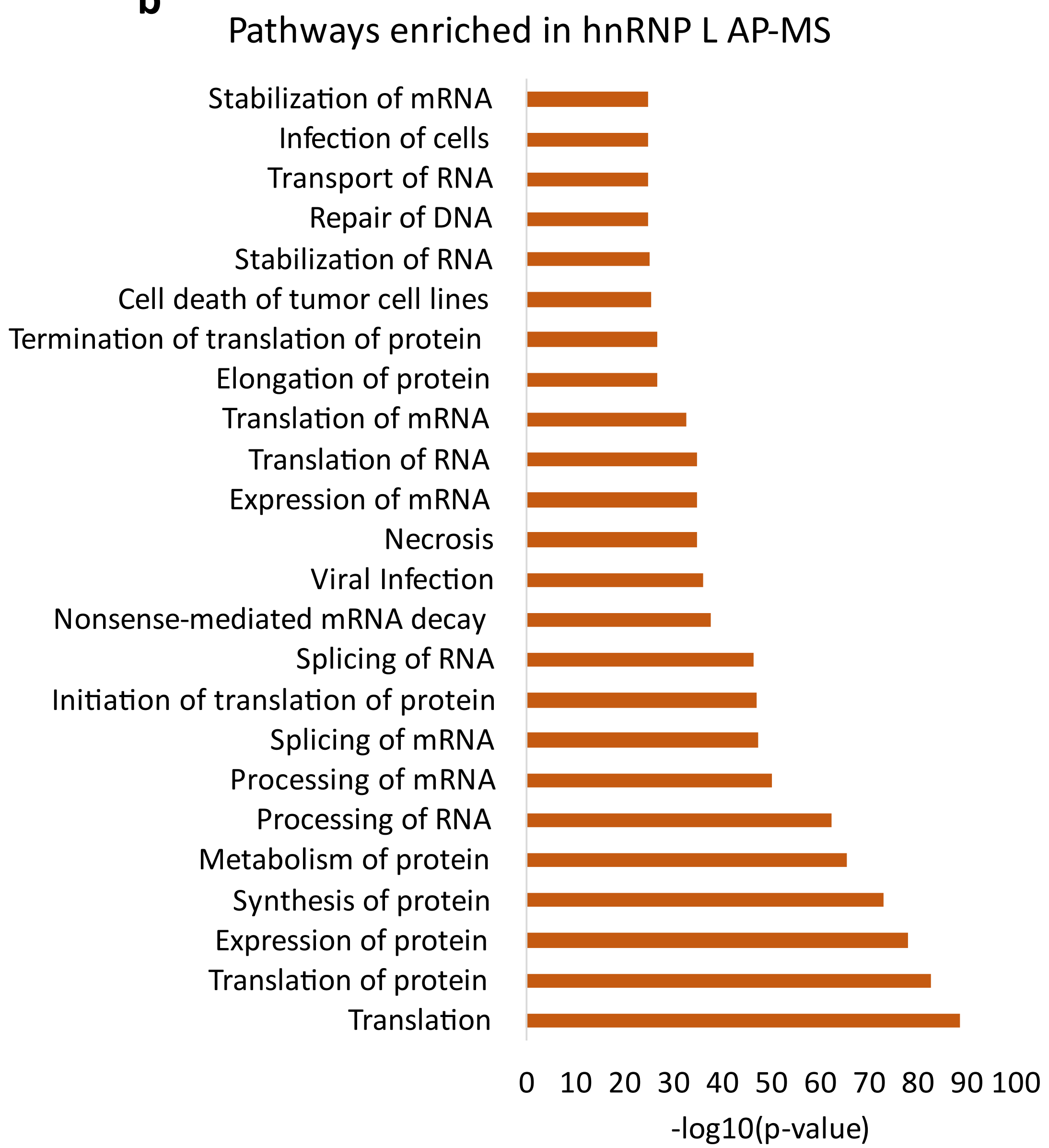

Supplementary Figure 1. **hnRNP L co-purifies proteins involved in RNA processing.** (a) Position, sequence and score of putative NLS (Nuclear Localization Signal) in hnRNP L based on NLS Mapper prediction. (b) IPA (Ingenuity Pathway Analysis) of proteins enriched in Halo-hnRNP L purification. AP-MS-Affinity Purification-Mass Spectrometry.

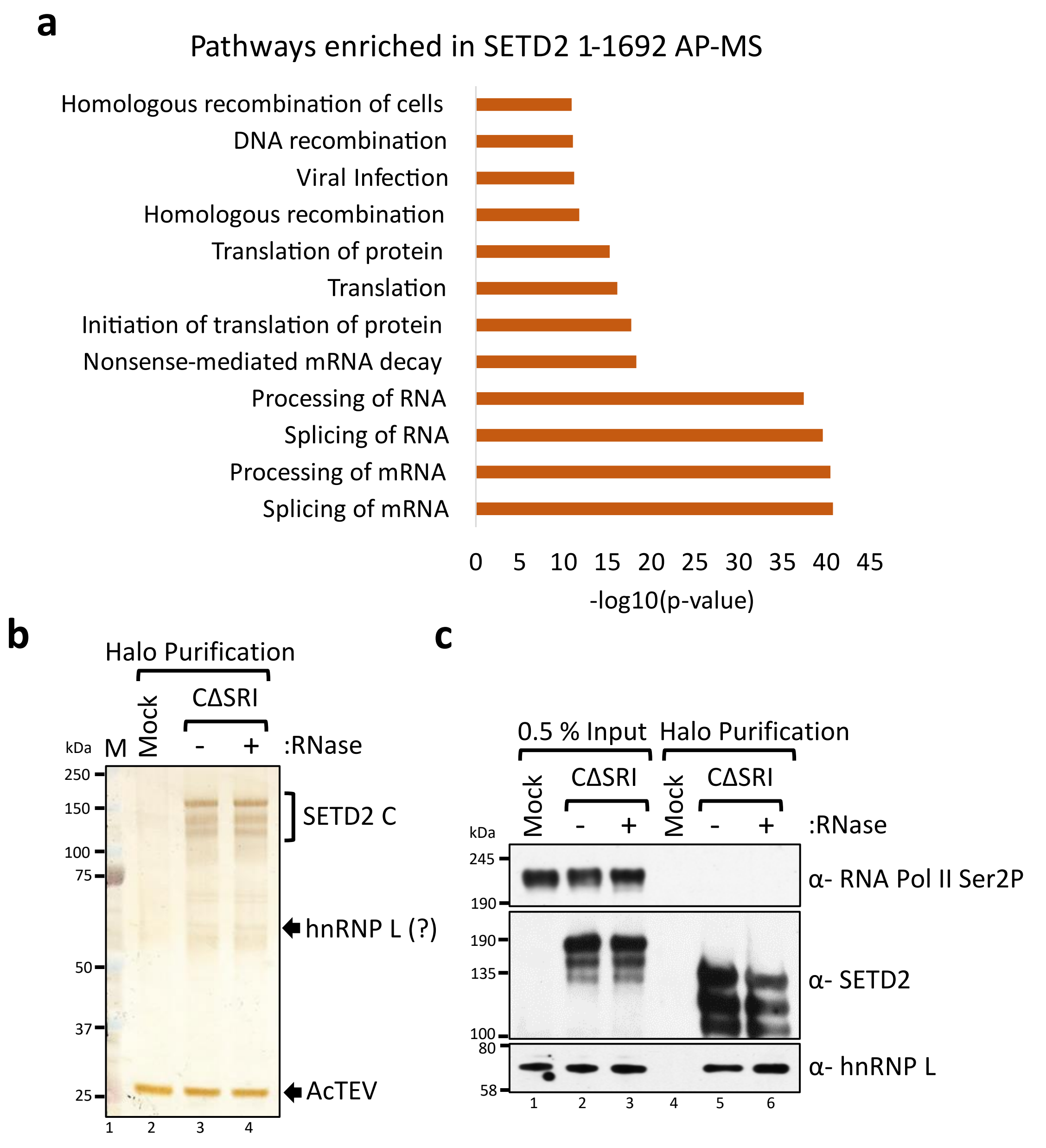

Supplementary Figure 2. **SETD2 co-purifies RNA binding proteins like hnRNP L.** (a) IPA (Ingenuity Pathway Analysis) of proteins enriched in Halo-SETD2 N + Catalytic Domains purification. (b, c) Halo purification was performed from extracts of 293T cells expressing Halo-SETD2CΔSRI. Input and eluted samples were resolved on a gel followed by silver staining or western blotting with the antibodies depicted. The experiment was repeated 2 times all yielding similar results. AP-MS-Affinity Purification-Mass Spectrometry, SRI-Set2-Rpb1 Interaction.

a

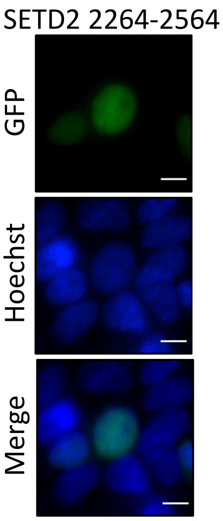

c

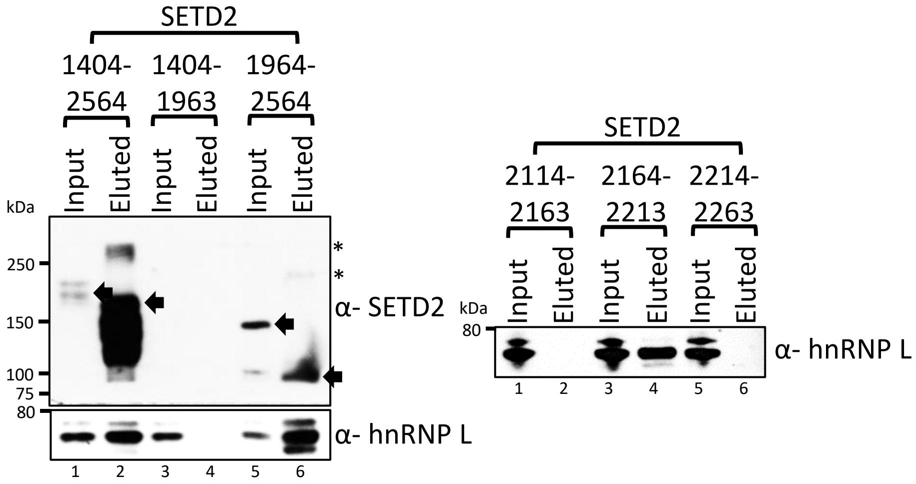

b

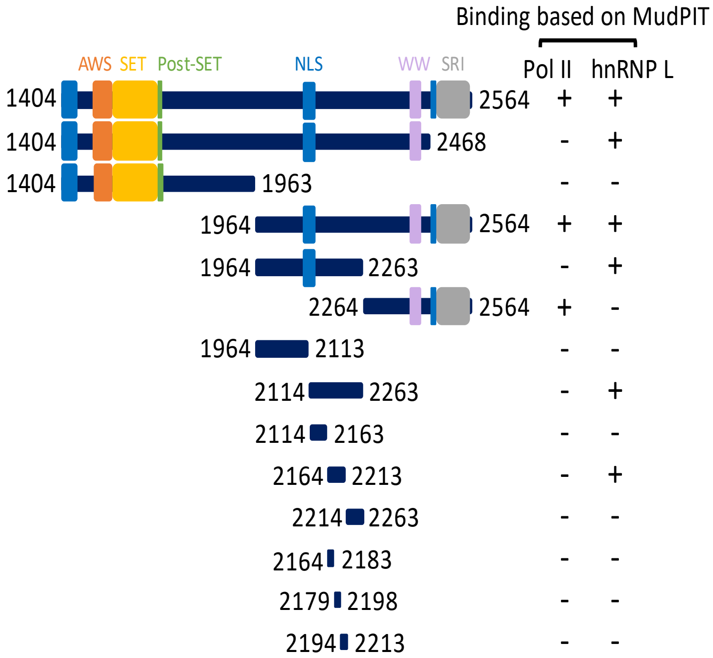

	dNSAF		
	Bait	Pol II	hnRNP L
1404-2564	0.130	0.000141	0.0586
1404-2468	0.133	-	0.0440
1404-1963	0.048	-	-
1964-2564	0.016	0.001596	0.04874
1964-2263	0.005	-	0.01227
2263-2564	0.013	0.000614	-
1964-2113	0.029	-	-
2114-2263	0.016	-	0.0617
2114-2163	0.004	-	-
2164-2213	0.165	-	0.0192
2214-2263	-	-	-
2164-2183	-	-	-
2179-2198	0.493	-	-
2194-2213	0.00002	-	-

d

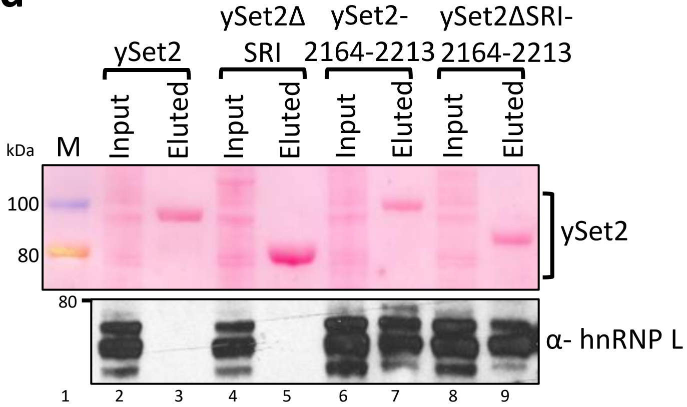

Supplementary Figure 3. **SETD2 2164-2213 is sufficient for hnRNP L interaction.** (a) Microscopy images showing localization of GFP- SETD2 2264-2564. The scale bar is 10 µm. The experiment was repeated at least 3 times all yielding similar results. (b) Cartoon illustrating the truncations of SETD2 along with the known domains used for the characterization of the hnRNP L- binding region. The table shows the dNSAFs of the listed proteins. (c, d) Halo purification was performed from extracts of 293T cells expressing Halo-SETD2/ySet2 constructs. Input (0.5%) and eluted samples were resolved on a gel followed by ponceau staining and western blotting. The expected band for the target proteins are marked by arrows. The experiment was repeated at least 2 times all yielding similar results. GFP-Green Fluorescent Protein, M-protein marker, AWS-Associated with SET, SET-Su(var)3-9, Enhancer-of-zeste and Trithorax, SRI-Set2-Rpb1 Interaction, dNSAF-distributed Normalized Spectral Abundance Factor, NLS-Nuclear Localization Signal.

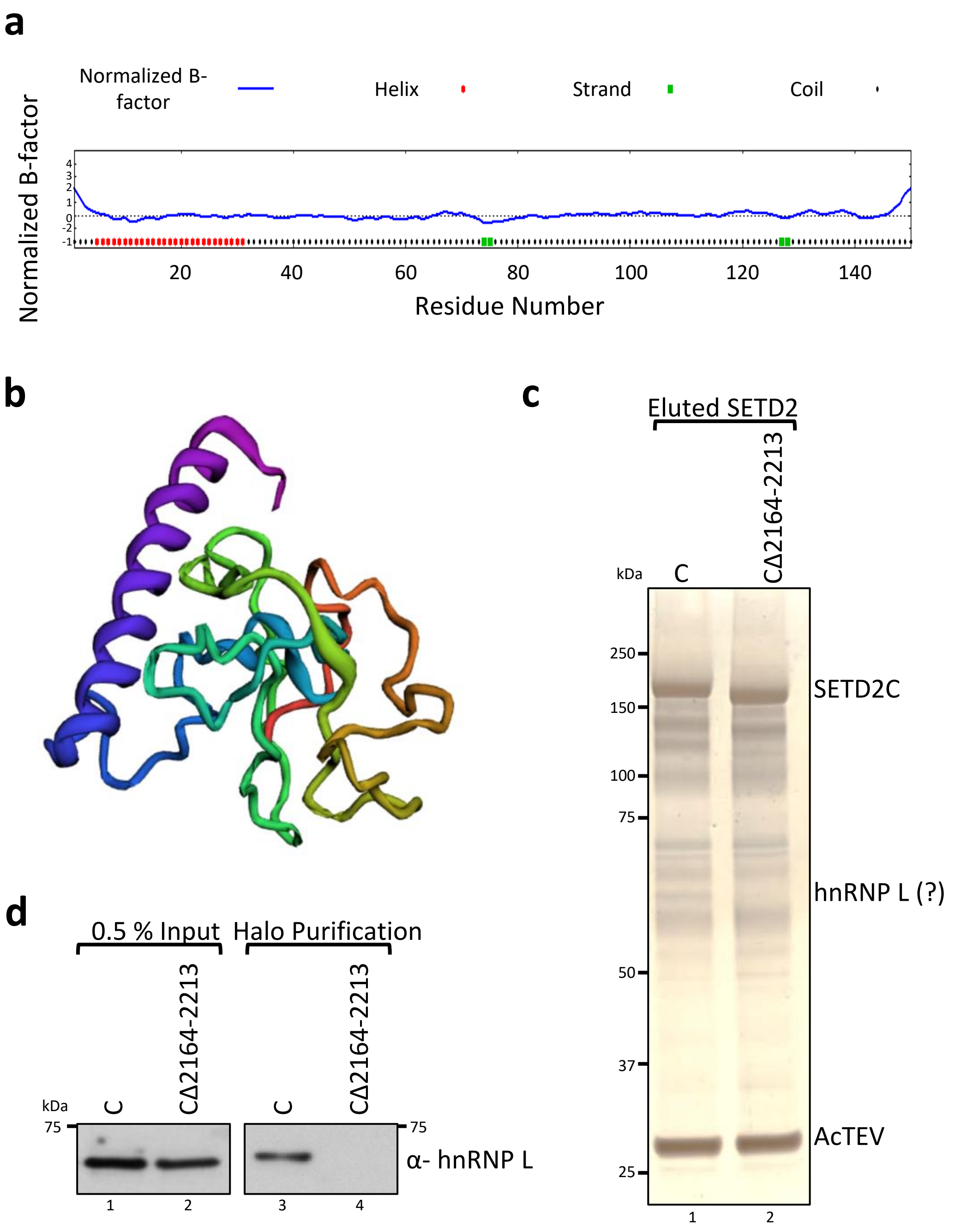

Supplementary Figure 4. **SETD2-SHI domain is unstructured.** (a) Prediction of the structure characteristic of the SHI domain by iTASSER server. (b) Modeled structure of the SETD2 SHI domain using *ab initio* method in Robetta server. (c, d) Halo purification was performed from extracts of 293T cells expressing Halo-SETD2C. Input and eluted samples were resolved on a gel followed by silver staining or western blotting. The experiment was repeated at least 2 times all yielding similar results.

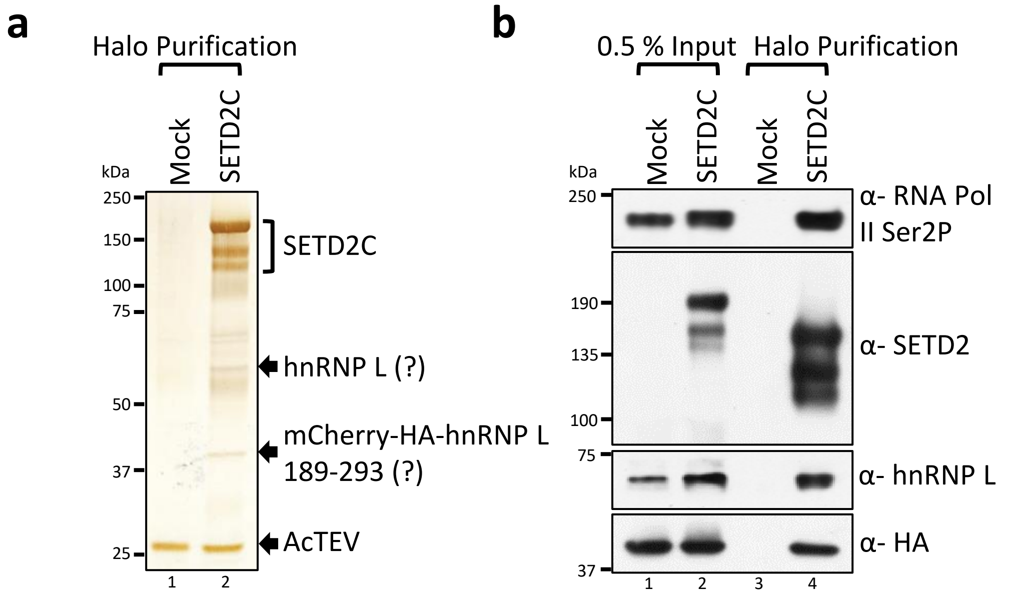

c

```
RRM4      --------------------RIQHPSNVLHFFN-APLEVTEENFFEICDELGVKRPSSVK  39
RRM2      ---------------------RSVNSVLLFTILNPIYSITTDVLYTICNPCGPVQR--IV  37
RRM1      -------------------------SPVVHIRGLIDGVVEADLV-EALQEFGPISY--VV  32
RRM3      PSRYGPQYGHPPPPPPPPEYGPHADSPVLMVYGLDQSKMNCDRVFNVFCLYGNVEK--VK  58
                                   * ::         :  : .       *      : 

RRM4      VFSGKSERSSSGLLEWESKSDALETLGFLNHYQMKNPNGPYPYTLKLCF-----------  88
RRM2      IFRKN---GVQAMVEFDSVQSAQRAKASLNGA------DIYSGCCTLKIE---YAKPTRL  85
RRM1      VMPKK----RQALVEFEDVLGACNAVNYAADN-------------QIYIA----------  65
RRM3      FMKSK---PGAAMVEMADGYAVDRAITHLNNN------FMFGQKLNVCVSKQPAIMPGQS  109
          .:  :      .::*  .   . .:                     : .           

RRM4      -------------------  88
RRM2      NVFK-NDQDTWDYTNP---  100
RRM1      -----GHPAFVNYSTSQKI  79
RRM3      YGLEDGSCSYKDFSES---  125
```

Supplementary Figure 5. **RRM2 of hnRNP L interacts with SETD2.** (a, b) Halo purification was performed from extracts of 293T cells co-expressing Halo-SETD2C and mCherry-HA-hnRNP L 189-293. Input and eluted samples were resolved on a gel followed by silver staining or western blotting with the antibodies depicted. The experiment was repeated 2 times all yielding similar results. (c) Clustal Omega (https://www.ebi.ac.uk/Tools/msa/clustalo/) sequence alignment of hnRNP L RRMs.

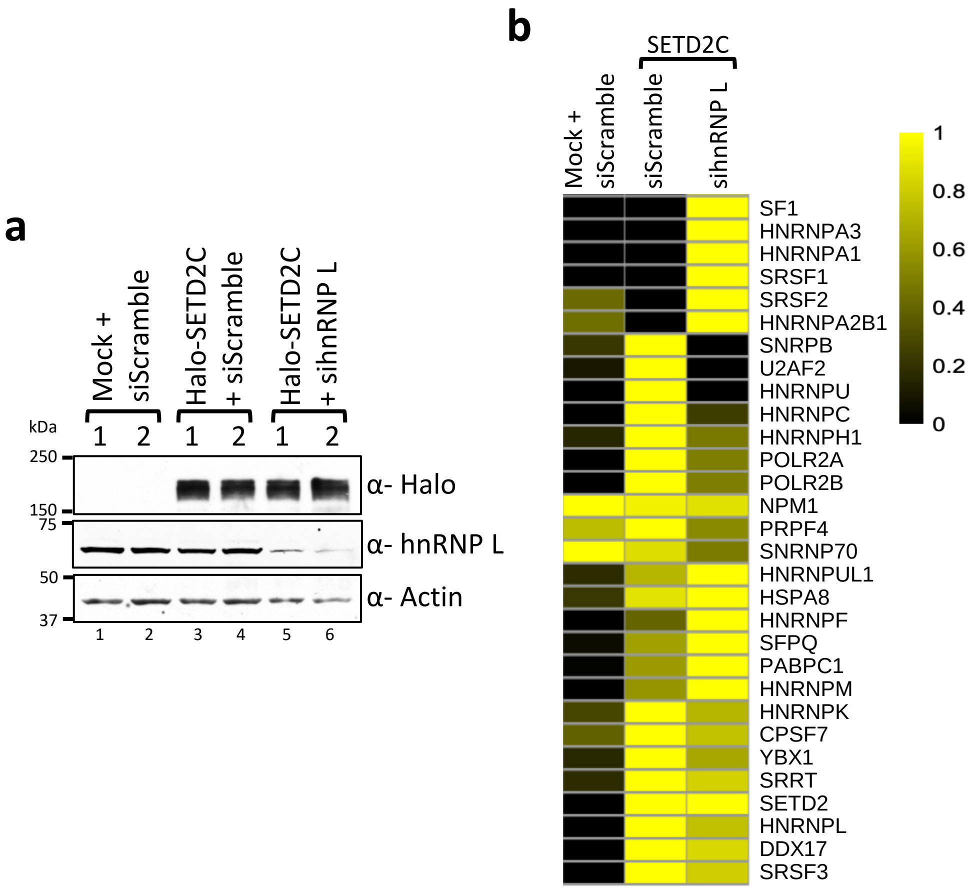

Supplementary Figure 6. **SETD2 interacts with many hnRNPs besides hnRNP L.** (a) Western blotting of whole cell extracts of 293T cells that were used for Halo purification followed by MudPIT. The experiment was repeated 2 times all yielding similar results. (b) Heat map showing the enrichment of proteins in MudPIT analysis.

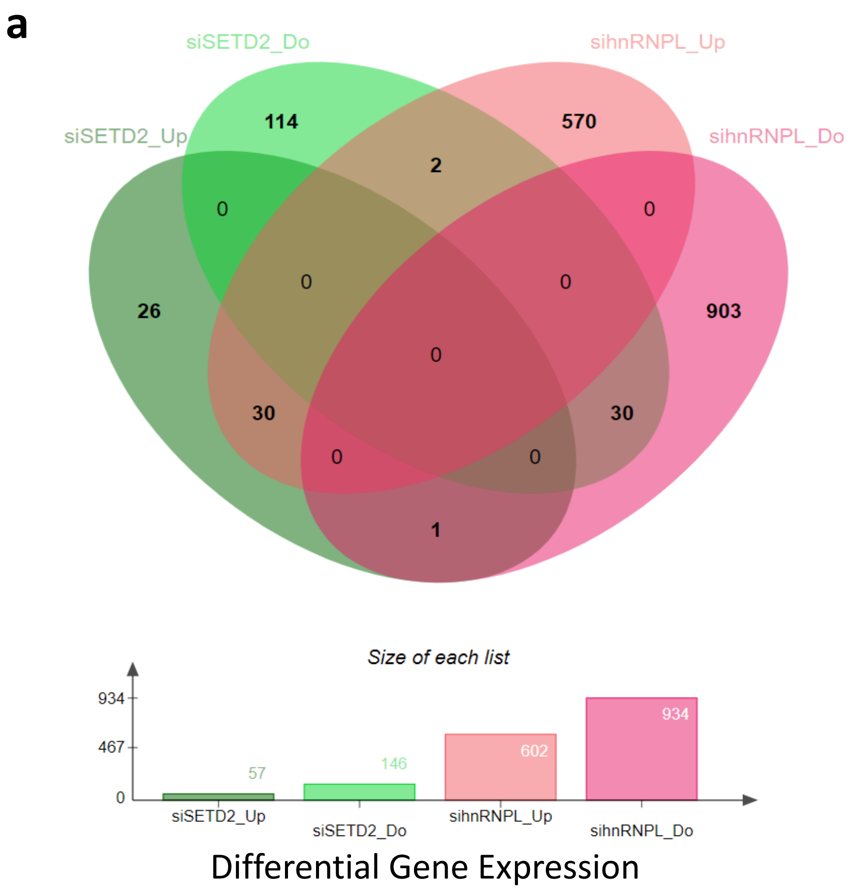

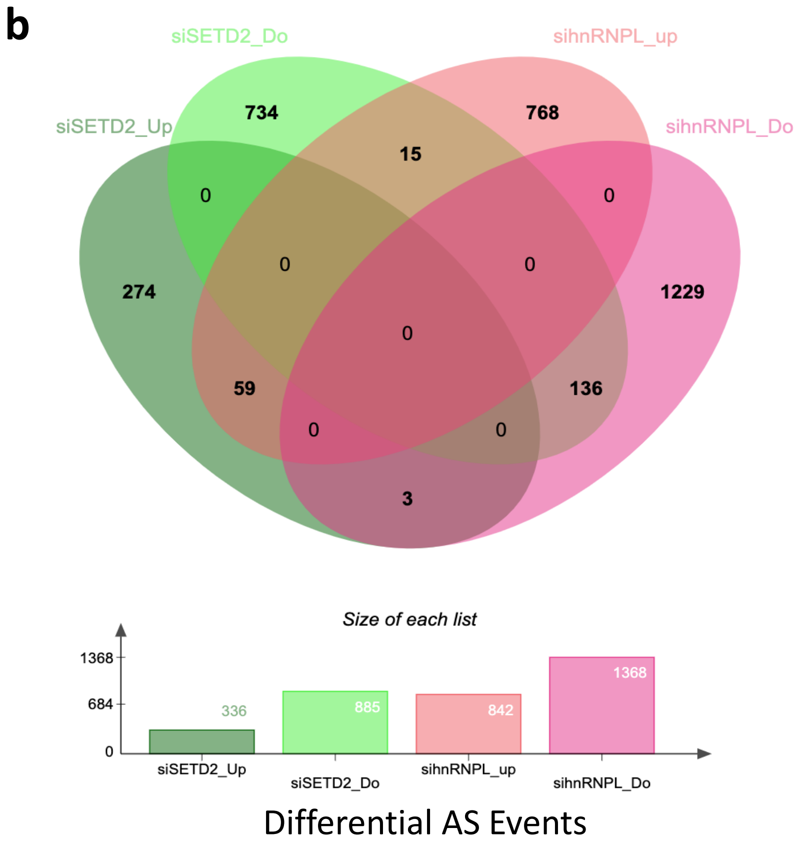

Supplementary Figure 7. **SETD2 and hnRNP L depletion causes transcriptome and alternative splicing (AS) changes.** (a, b) Venn diagram showing the overlaps of differentially expressed genes and AS events upon SETD2 and hnRNP L depletion. Do-Down.

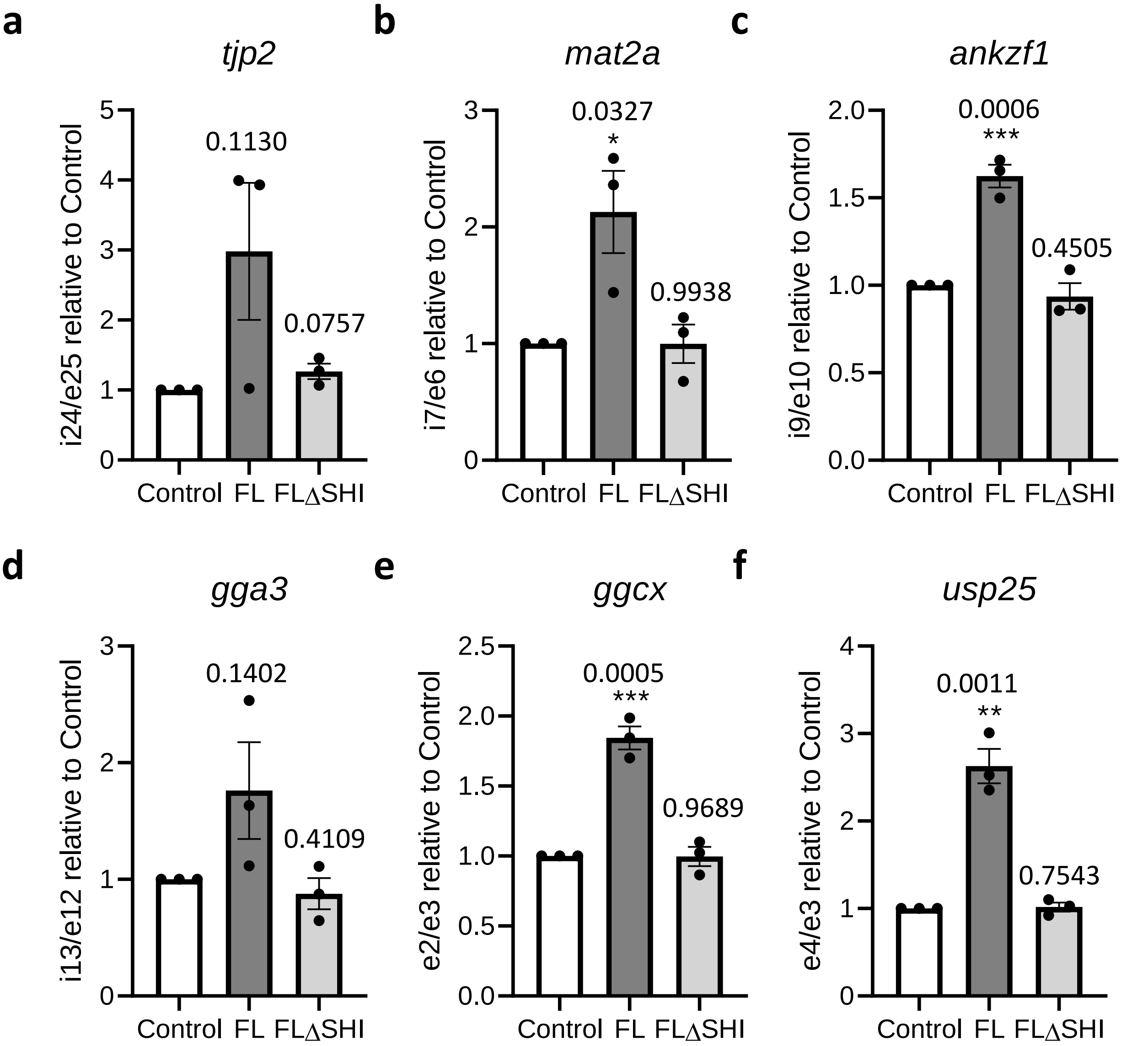

Supplementary Figure 8. **SETD2 SHI domain plays a role in alternative splicing.** (a-f) *setd2Δ* 293T cells were rescued with Vector Control, SETD2 FL and SETD2 FLΔSHI constructs and their RNA was isolated 72 hours post-transfection. Specific primers were designed to detect the indicated introns and exons and individual alternative splicing events were measured by quantitative PCR and represented by the ratios of intron to exon, or different exons. For each sample n = 3 independent biological samples examined in the same sequencing run. Data are presented as mean values with Standard Error of Mean. . Unpaired t test (two-tailed) was performed. p-value <0.05 was considered significant. p-values are depicted on the top of the respective graphs. SHI-SETD2-hnRNP Interaction.

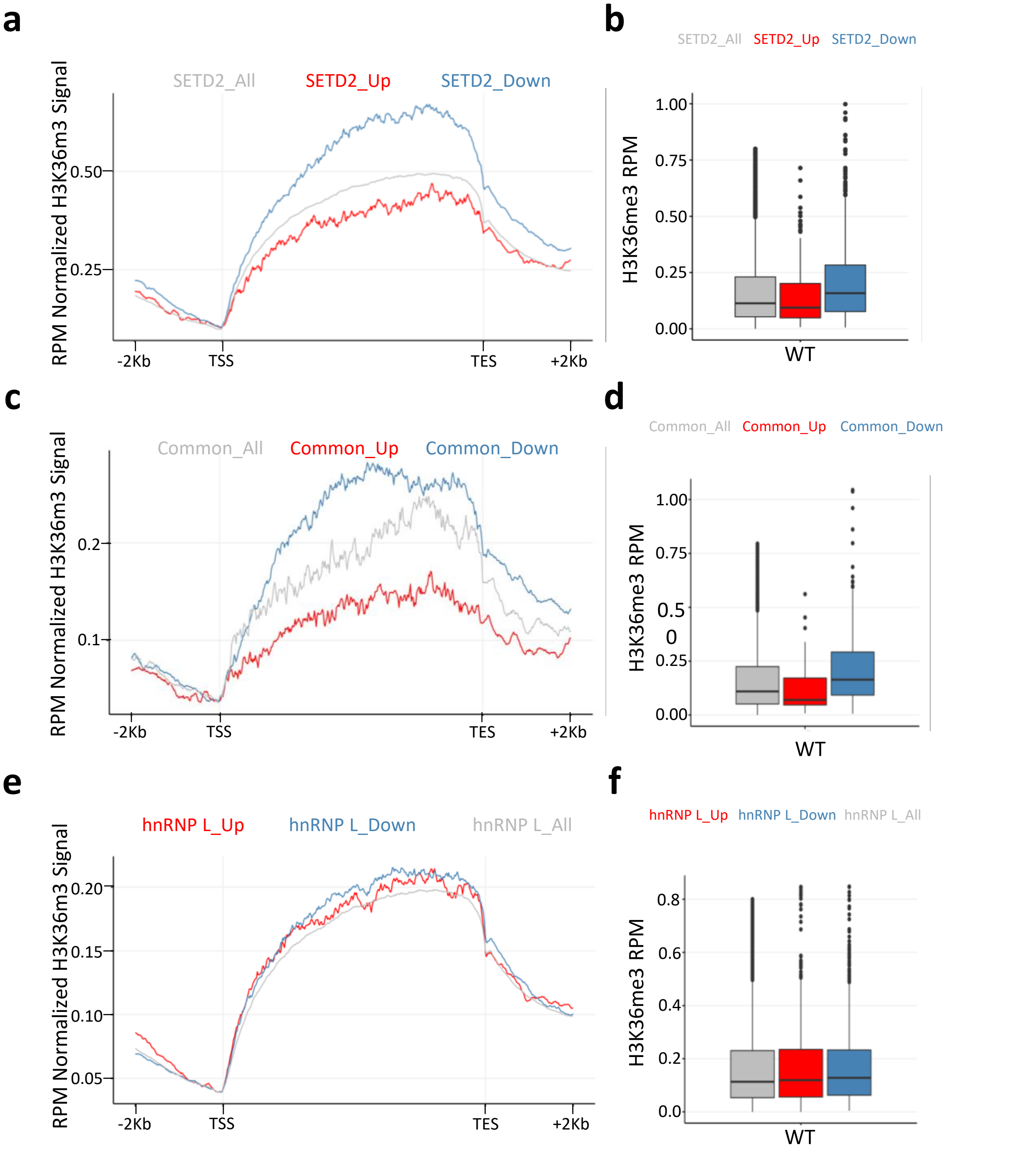

Supplementary Figure 9. **SETD2 and hnRNP L co-regulated splicing events correlate with H3K36me3 levels.** Metagene plots (a, c, e) and boxplots (b, d, f) depicting the distribution of H3K36me3 of genes that show differential alternative splicing upon SETD2 and hnRNP L depletion. For each sample n = 2 independent biological samples examined in the same sequencing run. RPM-Reads Per Million, TSS-Transcription Start Site, TES-Transcription End Site, WT-Wild Type. In the boxplots, the black line inside the box shows the median. The box bottom and top boarder correspond to 25th and 75th percentiles (Q1 and Q3 respectively). The whiskers represent ranges from Q1 – 1.5 * IQR to Q3 + 1.5 * IQR where IQR stands for interquartile range (Q3 – Q1). Data points outside the whiskers could be outliers and are marked as black dots.

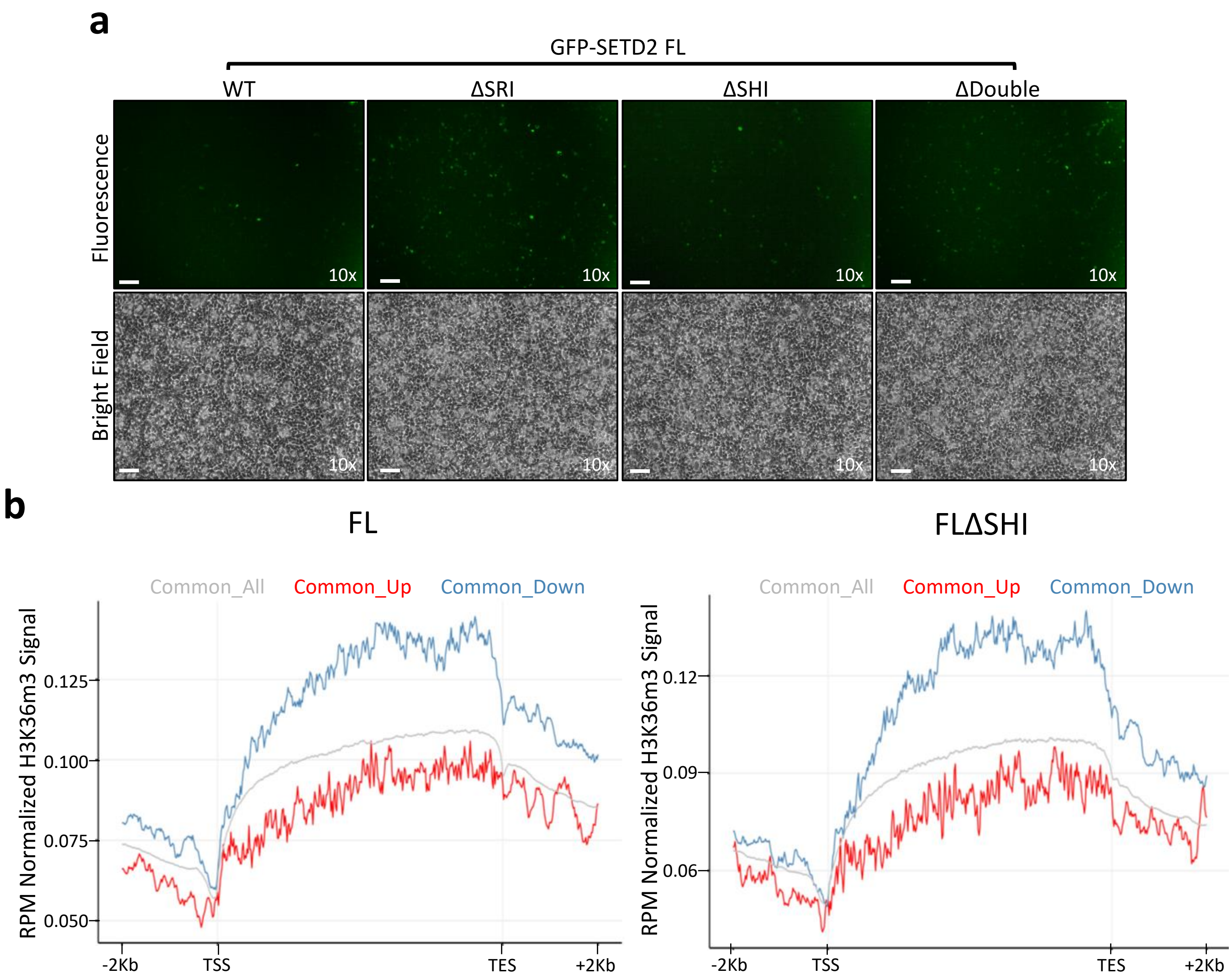

Supplementary Figure 10. **SETD2 FL and FLΔSHI show similar expression level and result in similar H3K36me3 distribution pattern.** (a) Microscopy images showing the expression of GFP-SETD2 FL mutants in 293T cells. The scale bar is 1 mm. (b) Metagene plot depicting the distribution of H3K36me3 of genes that show differential alternative splicing upon SETD2 and hnRNP L depletion after rescuing *setd2Δ* 293T cells SETD2 FL and SETD2 FLΔSHI. SRI-Set2-Rpb1 Interaction, SHI-SETD2-hnRNP Interaction, RPM-Reads Per Million, TSS-Transcription Start Site, TES-Transcription End Site.

Oligo	Sequence (5’-3’)
SETD2_F	AAGAAGCTCCCTCTCACCAC
SETD2_R	GATCCACATAGGCCTGCATG
hnRNPL_F	TTCTGCTTATATGGCAATGTGG
hnRNPL_R	GACTGACCAGGCATGATGG
GAPDH _F	TTCGACAGTCAGCCGCATCTTCTT
GAPDH _R	CAGGCGCCCAATACGACCAAATC
TJP2_C_F:	CACTCCAAGCGCGGTTACTA
TJP2_C_R:	GGAGTGGCTAGTCTGATGCC
TJP2_T_F:	TGAAACTGGCAGAGACCTCG
TJP2_T_R:	AGGTGCTCCCTGACCTAAGA
MAT2A_C_F:	TCCCATCAGAGTCCACACAA
MAT2A_C_R:	AAGGTATTTCGCAGGCACAAC
MAT2A_T_F:	GGCTTTCTGAAACCTACATGTGAA
MAT2A_T_R:	ACGCCTAGTGCTTTCCAAGTG
ANKZF1_C_F:	GCTGGAGATGTTGGAGTGCT
ANKZF1_C_R:	AGGAGAGTAAAGCCACCGGA
ANKZF1_T_F:	GATGCGTGGATGTGTTGCAT
ANKZF1_T_R:	GCCGCACAATAGAGGAAAGC
GGA3_C_F:	AGGATTGGGATGCCTGAGGA
GGA3_C_R:	CAGTAACCAAGGCACGCTCA
GGA3_T_F:	GCTCCAGTCCATGATGCAGA
GGA3_T_R:	CAGTGAAGTTCAGCGACCCT
USP25_C_F:	GCGAAGAATGCTAAGACCCC
USP25_C_R:	GTATCTGCTTGGCTTCCCACA
USP25_T_F:	TCTCACTGGAGATGATAAAGATGAT
USP25_T_R:	GTCTCCCTGAATGCCCTGTT
GGCX_C_F:	CAGTTCCCCTCACCCAGAAAC
GGCX_C_R:	ACCTTGATGGGCTGGATGTG
GGCX_T_F:	TAAGCTTGCAGGGTCCGTTG
GGCX_T_R:	CAGGACAGCCGAATAGGGAAA

Supplementary Table 1. Sequence of oligos used to perform PCR.
