# Supplementary material for: The methyltransferase SETD2 couples transcription and splicing by engaging mRNA processing factors through its SHI domain
Source: Nat Commun. 2021 Mar 4;12:1443. doi: 10.1038/s41467-021-21663-w (PMC7933334; doi:10.1038/s41467-021-21663-w)
Supplement: Supplementary file 3 — Description of Additional Supplementary Files [file 41467_2021_21663_MOESM3_ESM.pdf]

Supplementary Data 1. Proteins identified by hnRNP L 162-321 fragment as the bait either treated with or without RNase.

Supplementary Data 2. The proteins co-purified with Halo-SETD2 1-1692.

Supplementary Data 3. Proteins that interact with SETD2C truncations as identified by MudPIT mass spectrometry.

Supplementary Data 4. Proteins that interact with Halo-Set2 as identified by MudPIT mass spectrometry.

Supplementary Data 5. Proteins interaction with various SETD2C truncations including SETD2CΔSRI, SETD2CΔSHI, and SETD2C-SHI.

Supplementary Data 6. Proteins identified by MudPIT analysis when 293T cells were treated with a scrambled RNAi or RNAi against hnRNP L.

Supplementary Data 7. RNA-seq Differential Expression Analysis Report for hnRNP L depleted cells vs WT.

Supplementary Data 8. Splicing changes in SETD2 and hnRNP L depleted cells.
