# Supplementary material for: The methyltransferase SETD2 couples transcription and splicing by engaging mRNA processing factors through its SHI domain
Source: Nat Commun. 2021 Mar 4;12:1443. doi: 10.1038/s41467-021-21663-w (PMC7933334; doi:10.1038/s41467-021-21663-w)
Supplement: Supplementary file 5 — Reporting Summary [file 41467_2021_21663_MOESM5_ESM.pdf]

nature research

Corresponding author(s): Jerry Workman

Last updated by author(s): Feb 2, 2021

Reporting Summary

Nature Research wishes to improve the reproducibility of the work that we publish. This form provides structure for consistency and transparency in reporting. For further information on Nature Research policies, see our Editorial Policies and the Editorial Policy Checklist.

Statistics

For all statistical analyses, confirm that the following items are present in the figure legend, table legend, main text, or Methods section.

n/a	Confirmed	
☐	☒	The exact sample size (*n*) for each experimental group/condition, given as a discrete number and unit of measurement
☒	☐	A statement on whether measurements were taken from distinct samples or whether the same sample was measured repeatedly
☐	☒	The statistical test(s) used AND whether they are one- or two-sided<br>*Only common tests should be described solely by name; describe more complex techniques in the Methods section.*
☒	☐	A description of all covariates tested
☒	☐	A description of any assumptions or corrections, such as tests of normality and adjustment for multiple comparisons
☐	☒	A full description of the statistical parameters including central tendency (e.g. means) or other basic estimates (e.g. regression coefficient) AND variation (e.g. standard deviation) or associated estimates of uncertainty (e.g. confidence intervals)
☐	☒	For null hypothesis testing, the test statistic (e.g. *F*, *t*, *r*) with confidence intervals, effect sizes, degrees of freedom and *P* value noted<br>*Give P values as exact values whenever suitable.*
☒	☐	For Bayesian analysis, information on the choice of priors and Markov chain Monte Carlo settings
☒	☐	For hierarchical and complex designs, identification of the appropriate level for tests and full reporting of outcomes
☒	☐	Estimates of effect sizes (e.g. Cohen's *d*, Pearson's *r*), indicating how they were calculated

Our web collection on statistics for biologists contains articles on many of the points above.

Software and code

Policy information about availability of computer code

Data collection	Mass spectrometer scan functions and HPLC solvent gradients were controlled by the XCalibur 2.0.7 data system (Thermo Scientific). ChIP-Seq samples (single reads of 50 bp) were run on an Illumina HiSeq 2500, using HiSeq Control Software v2.2.58, with RTA v1.18.64. RNA-Seq samples (paired end reads 75 bp) were run on the Illumina NextSeq 500, using NextSeq Control Software 2.2.0.4, with NextSeq RTA 2.4.11.
Data analysis	Mass spectrometry RAW files were extracted into .ms2 file format using RawDistiller v. 1.0. MS/MS spectra were first searched using ProLuCID. DTASelect v.1.9 44 was used to select and sort peptide/spectrum matches (PSMs) passing the following criteria set: PSMs were only retained if they had a DeltCn of at least 0.08; minimum XCorr values of 2.1 for singly-, 2.7 for doubly-, and 3.2 for triply-charged spectra; peptides had to be at least 7 amino acids long. Results from each sample were merged and compared using CONTRAST v.1.9. Combining all replicates, proteins had to be detected by at least 2 peptides and/or 2 spectral counts. Proteins that were subsets of others were removed using the parsimony option in DTASelect v.1.9 44 on the proteins detected after merging all runs. NSAF7 was used to create the final reports on all detected peptides and non-redundant proteins identified across the different runs. QPROT v. 1.2.2 was used to calculate a log fold change and Z-score for the samples compared to the mock control. proteins with log fold change >1 and Z-score > 2 were further analyzed in Ingenuity Pathway Analysis (IPA, Qiagen) to determine pathways enriched by the bait proteins.<br>For nucleic acid sequencing data, Raw reads were demultiplexed into FASTQ format allowing up to one mismatch using Illumina bcl2fastq2 v2.18. Reads were aligned to the human genome (hg38) using Bowtie2 (version 2.3.4.1) with default parameters. The average normalized (RPM or spike-in) H3K36me3 signals in each bin were plotted using R package EnrichedHeatmap. RNA seq reads were aligned to the human genome (hg38 and Ensembl 96 gene models) using STAR (version STAR_2.6.1c). TPM expression values were generated using RSEM (version v1.3.0). edgeR (version 3.24.3 with R 3.5.2) was applied to perform differential expression analysis, using only protein-coding and lncRNA genes. To perform differential splicing analysis, we used rMATs (version 4.0.2) with default parameters starting from FASTQ files. For spike-in ChIP-seq data, we also generated spike-in normalized bigWig tracks. R packages GenomicRanges (1.38) and rtracklayer (1.46) were used to generate bigWig files.

For manuscripts utilizing custom algorithms or software that are central to the research but not yet described in published literature, software must be made available to editors and reviewers. We strongly encourage code deposition in a community repository (e.g. GitHub). See the Nature Research guidelines for submitting code & software for further information.

Data

Policy information about availability of data

All manuscripts must include a data availability statement. This statement should provide the following information, where applicable:

- Accession codes, unique identifiers, or web links for publicly available datasets
- A list of figures that have associated raw data
- A description of any restrictions on data availability

All relevant data are available. The data sets are publicly available in the Gene Expression Omnibus (GEO) database under the accession number GSE151296. The mass spectrometry proteomics data have been deposited to the ProteomeXchange Consortium via the PRIDE partner repository with the dataset identifier PXD019376 and 10.6019/PXD019376. Additionally, the SETD2C truncation variants for Figure 3 have been deposited with the dataset identifier PXD019538 and 10.6019/PXD019538. Also, the data for Supplementary Figure 6b have been deposited with the dataset identifier PXD022946 and 10.6019/PXD022946. Original data underlying this manuscript can be accessed from the Stowers Original Data Repository at http://www.stowers.org/research/publications/libpb-1582 after the manuscript has been officially accepted.

List of figures that have associated raw data:
Figure 1, 2, 3, 5, 6, 7
Supplementary Figure 1, 2, 3, 6, 7, 9, 10

Field-specific reporting

Please select the one below that is the best fit for your research. If you are not sure, read the appropriate sections before making your selection.

☒ Life sciences ☐ Behavioural & social sciences ☐ Ecological, evolutionary & environmental sciences

For a reference copy of the document with all sections, see nature.com/documents/nr-reporting-summary-flat.pdf

Life sciences study design

All studies must disclose on these points even when the disclosure is negative.

Sample size	Our study did not involve any organisms or human samples or clinical research. Two independent biological replicates were used in high throughput experiments. As the replicates were in very good agreement with one another, hence, the sample size was considered sufficient.
Data exclusions	No data was excluded.
Replication	Experiments were replicated by using biologically independent replicates at least twice and by utilizing more than one technique to confirm the findings. For instance, crucial mass spectrometry findings were also confirmed by western blotting of the purified complexes. Depletion of the target protein was confirmed by western blotting, RT-PCR before proceeding for high through-put sequencing. ChIP-Seq was performed post observing expected enrichment by ChIP-PCR. All attempts at replication were successful.
Randomization	The interpretation of the experiments performed by us required comparison of data from experimental sets versus the control set. For instance, for mass spectrometry experiments, data from known samples of interest were compared against mock samples. For RNA-Seq experiments, known samples of interest were first compared against known scramble siRNA treated cells. Hence, randomization was not possible.
Blinding	The interpretation of the experiments performed by us required comparison of data from experimental sets versus the control set. For instance, for mass spectrometry experiments, data from known samples of interest were compared against mock samples. For RNA-Seq experiments, known samples of interest were first compared against known scramble siRNA treated cells. Hence, the identity of the control and the experimental sets were required to be known.

Reporting for specific materials, systems and methods

We require information from authors about some types of materials, experimental systems and methods used in many studies. Here, indicate whether each material, system or method listed is relevant to your study. If you are not sure if a list item applies to your research, read the appropriate section before selecting a response.

Materials & experimental systems

n/a	Involved in the study
	☒ Antibodies
	☒ Eukaryotic cell lines
☒	☐ Palaeontology and archaeology
☒	☐ Animals and other organisms
☒	☐ Human research participants
☒	☐ Clinical data
☒	☐ Dual use research of concern

Methods

n/a	Involved in the study
	☒ ChIP-seq
☒	☐ Flow cytometry
☒	☐ MRI-based neuroimaging

Antibodies

Antibodies used

hnRNP L (CST, 37562), FLAG (Sigma-Aldrich, A8592) , Pol II (Abcam, ab5095), Halo (Promega, G9211), SETD2 (Abclonal, A3194), HA (Sigma, 04-902), His (Abcam, ab18184), H3K36me3 (CST, 4909S) H3 (CST, 9715S), β-actin (Abcam, ab8224).

Validation

Commercially available antibodies were used in the study that are validated as per published reports and the manufacturer. Further validation was performed by us as follows:
1) hnRNP L: Depletion of hnRNP L resulted in the decreased intensity of the expected band [Figure 6a]. Product website (https://www.cellsignal.com/products/primary-antibodies/hnrnp-l-antibody/37562) states "Specificity / Sensitivity: hnRNP L Antibody recognizes endogenous levels of total hnRNP L protein.
Species Reactivity: Human, Mouse, Rat, Monkey."
2) H3K36me3: Depletion of SETD2 methyltransferase results in in the decreased intensity of H3K36me3 [Figure 6a]. Also, our ChIP-seq data using this antibody exhibits the expected enrichment of the peaks obtained. Product website (https://www.cellsignal.com/products/primary-antibodies/tri-methyl-histone-h3-lys36-d5a7-xp-rabbit-mab/4909?site-search-type=Products&N=4294956287&Ntt=h3k36me3&fromPage=plp) states "Specificity / Sensitivity: Tri-Methyl-Histone H3 (Lys36) (D5A7) XP® Rabbit mAb detects endogenous levels of histone H3 only when tri-methylated on Lys36. The antibody does not cross-react with non-methylated, mono-methylated, or di-methylated Lys36. In addition, the antibody does not cross-react with histone H3 methylated at Lys4, Lys9, Lys27 or histone H4 methylated at Lys20.
Species Reactivity: Human, Mouse, Rat, Monkey."
3) FLAG, HA, His: Probing lysates of untrasfected mammalian cells and untransformed bacterial cells do not show bands. The transfected/transformed cells show band of the expected size. FLAG: Product website (https://www.sigmaaldrich.com/catalog/product/sigma/a8592?lang=en&region=US) states that the Monoclonal ANTI-FLAG M2-Peroxidase is a mouse IgG antibody covalently conjugated to horseradish peroxidase (HRP). The antibody binds to FLAG fusion proteins and recognizes the FLAG epitope at N-terminal, Met-N-terminal, C-terminal, and internal FLAG peptides. HA: Product website (https://www.sigmaaldrich.com/catalog/product/mm/04902?lang=en&region=US) states that the antibody recognizes recombinant proteins containing the HA epitope tag. His: Product website (https://www.abcam.com/6x-his-tag-antibody-hish8-ab18184.html) states "Specificity: Recognizes His-tagged recombinant proteins or His-tagged proteins overexpressed in cells. ab18184 reacts to recombinant proteins containing the 6X His tag® or 10X His tag® fused to either the amino or carboxy terminus. Species reactivity: Species independent."
4) Pol II: This antibody has been successfully used by us to demonstrate that SETD2 lacking the SRI domain loses interaction with RNA Pol II. Mass spectrometry data of the purified complexes validate these findings (Figure 3). Product website (https://www.abcam.com/rna-polymerase-ii-ctd-repeat-ysptsps-phospho-s2-antibody-ab5095.html) states "Speicificity: This antibody recognises the phosphorylated serine found in the amino acid 2 position of the C-terminal domain repeat YSPTSPS. Species reactivity: Mouse, Rat, Human, Saccharomyces cerevisiae."
5) H3: Besides the expected size on a western blot, our ChIP-seq data using this antibody exhibits the expected distribution of the peaks obtained. Product website (https://www.cellsignal.com/products/primary-antibodies/histone-h3-antibody/9715) states "Specificity / Sensitivity: Histone H3 Antibody detects endogenous levels of total histone H3 protein. This antibody does not cross-react with other histones.
Species Reactivity: Human, Mouse, Rat, Monkey, Zebrafish, Bovine, Pig"

Eukaryotic cell lines

Policy information about cell lines

Cell line source(s)	HEK293T from ATCC
Authentication	STR analysis
Mycoplasma contamination	All cell lines tested negative for mycoplasma contamination.
Commonly misidentified lines (See ICLAC register)	No such cell lines were used in the study.

ChIP-seq

Data deposition

☒ Confirm that both raw and final processed data have been deposited in a public database such as GEO.

☒ Confirm that you have deposited or provided access to graph files (e.g. BED files) for the called peaks.

Data access links<br>*May remain private before publication.*	https://www.ncbi.nlm.nih.gov/geo/query/acc.cgi?acc=GSE151296
Files in database submission	GSM4572151 wt 1.h3k36me3<br>GSM4572152 wt 2.h3k36me3<br>GSM4572153 WT scramble 1<br>GSM4572154 WT scramble 2<br>GSM4572155 WT siSETD2 2<br>GSM4572156 WT siSETD2 3<br>GSM4572157 WT sihnRNPL 1<br>GSM4572158 WT sihnRNPL 2<br>GSM4965970 fl_1.h3k36me3.batch1<br>GSM4965971 fl_2.h3k36me3.batch1<br>GSM4965972 flwoshi_1.h3k36me3.batch1<br>GSM4965973 flwoshi_2.h3k36me3.batch1<br>GSM4965974 fl_1.h3k36me3.batch2<br>GSM4965975 fl_2.h3k36me3.batch2<br>GSM4965976 flwosri_1.h3k36me3.batch2<br>GSM4965977 flwosri_2.h3k36me3.batch2
Genome browser session<br>(e.g. UCSC)	Genome browser track files (in bigwig format) are available under supplementary files for GSE151296

Methodology

Replicates: Biological replicates, two replicates, very good agreement

Sequencing depth

Sample	Total Reads/Pairs	Uniquely Aligned Reads/Pairs	Experiment Type	Seq Type	Read Length
wt_1.h3k36me3	78215270	48703501	ChIP-seq	Single	51
wt_2.h3k36me3	74319963	46969611	ChIP-seq	Single	51
fl_1.h3k36me3.batch1	77625091	56412229	ChIP-seq	Single	51
fl_2.h3k36me3.batch1	82176129	60044238	ChIP-seq	Single	51
flwoshi_1.h3k36me3.batch1	57712278	41160417	ChIP-seq	Single	51
flwoshi_2.h3k36me3.batch1	62201674	36964565	ChIP-seq	Single	51
Sample	Total Reads/Pairs	Uniquely Aligned Reads/Pairs	Experiment Type	Seq Type	Read Length
fl_1.h3k36me3.batch2	37541319	27309116	ChIP-seq	Single	51
fl_2.h3k36me3.batch2	63296635	44393833	ChIP-seq	Single	51
flwosri_1.h3k36me3.batch2	49586871	36180246	ChIP-seq	Single	51
flwosri_2.h3k36me3.batch2	59206193	42519227	ChIP-seq	Single	51
wt_scramble_1	54919637	45689102	RNA-seq	Paired-End	76
wt_scramble_2	51837600	42001447	RNA-seq	Paired-End	76
wt_sisetd2_2	51619570	41178899	RNA-seq	Paired-End	76
wt_sisetd2_3	64837830	56486926	RNA-seq	Paired-End	76
wt_sihnrnpl_1	53458881	43605321	RNA-seq	Paired-End	76
wt_sihnrnpl_2	54748878	47193521	RNA-seq	Paired-End	76

Antibodies: H3K36me3 (CST, 4909S)

Peak calling parameters:

Raw reads were demultiplexed into FASTQ format allowing up to one mismatch using Illumina bcl2fastq2 v2.18. Reads were aligned to human genome (hg38) using Bowtie2 (version 2.3.4.1) with default parameters. For samples with fly spike-in, reads were first mapped to Drosophila melanogaster genome (dm6), and unmapped reads were then aligned to human genome (hg38).

Reads per million (RPM) normalized bigWig tracks were generated by extending reads to 150bp. For spike-in ChIP-seq data, we also generated spike-in normalized bigWig tracks (RPM normalization factor = 1E6 / number of reads aligned to hg38, and spike-in normalization factor = 1E6 / number of reads aligned to dm6).

epic2 (with options: -gn hg38 -fs 200 -fdr 0.05) was used to call wide peaks for H3K36me3 ChIP-seq data for FL, N3, and N3ΔSRI. Next, R package ChIPseeker was applied (with options: genomicAnnotationPriority=c('Intergenic', '5UTR', '3UTR', 'Exon', 'Intron')) to obtain the genomic feature distribution (Ensembl 96 release) under peaks.

Data quality: The most majority of ChIP-seq samples have more than 40 million uniquely mapped reads. Cross-correlation and ChanceQC plots show good IP enrichment. Correlation heatmaps show good correlation between replicates.

Peaks with FDR < 0.05 are used to plot the genomic features under peaks.

Software	bcl2fastq2 (v2.18)<br>Bowtie2 (version 2.3.4.1)<br>R (3.5.2)<br>epic2
