# Supplementary material for: The methyltransferase SETD2 couples transcription and splicing by engaging mRNA processing factors through its SHI domain
Source: Nat Commun. 2021 Mar 4;12:1443. doi: 10.1038/s41467-021-21663-w (PMC7933334; doi:10.1038/s41467-021-21663-w)
Supplement: Supplementary file 6 — Source Data [file 41467_2021_21663_MOESM6_ESM.pdf]

Figure 1

Figure 1c

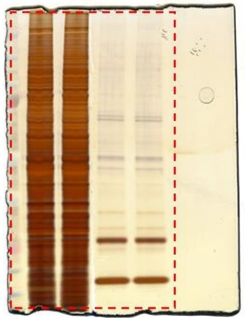

Silver Staining

Figure 1d

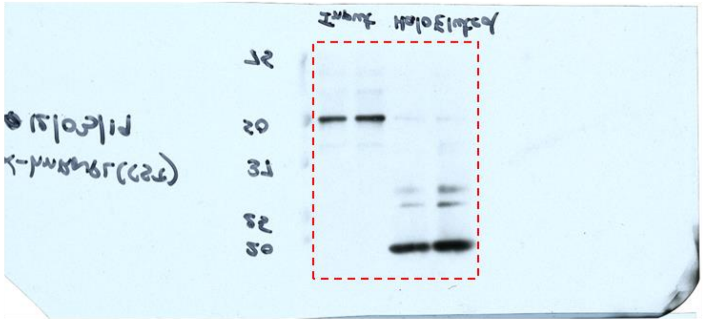

α-hnRNP L

Figure 1d

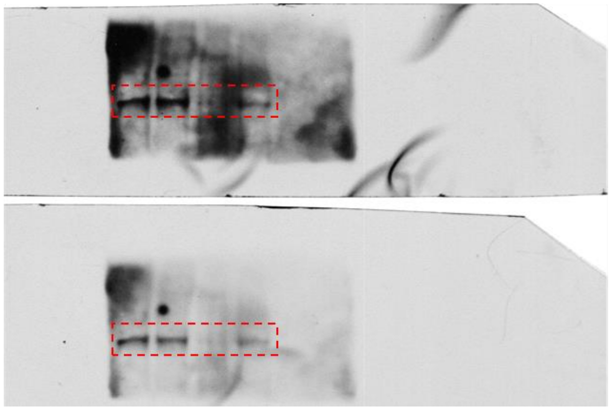

α-SETD2

Figure 2

Figure 2c

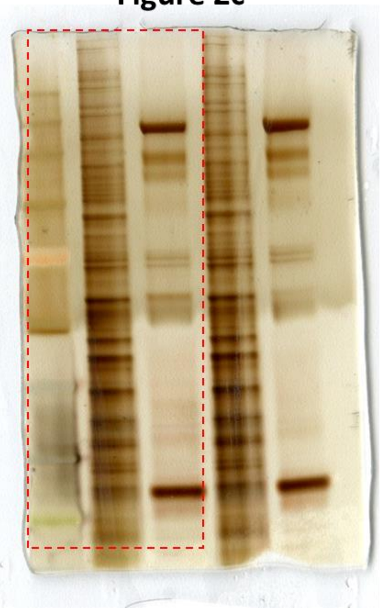

Silver Staining

Figure 2f

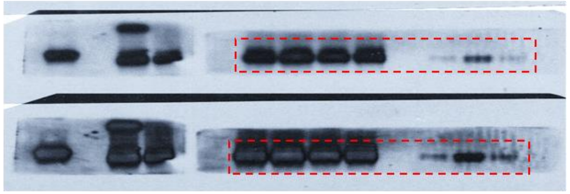

α- hnRNP L

Figure 3

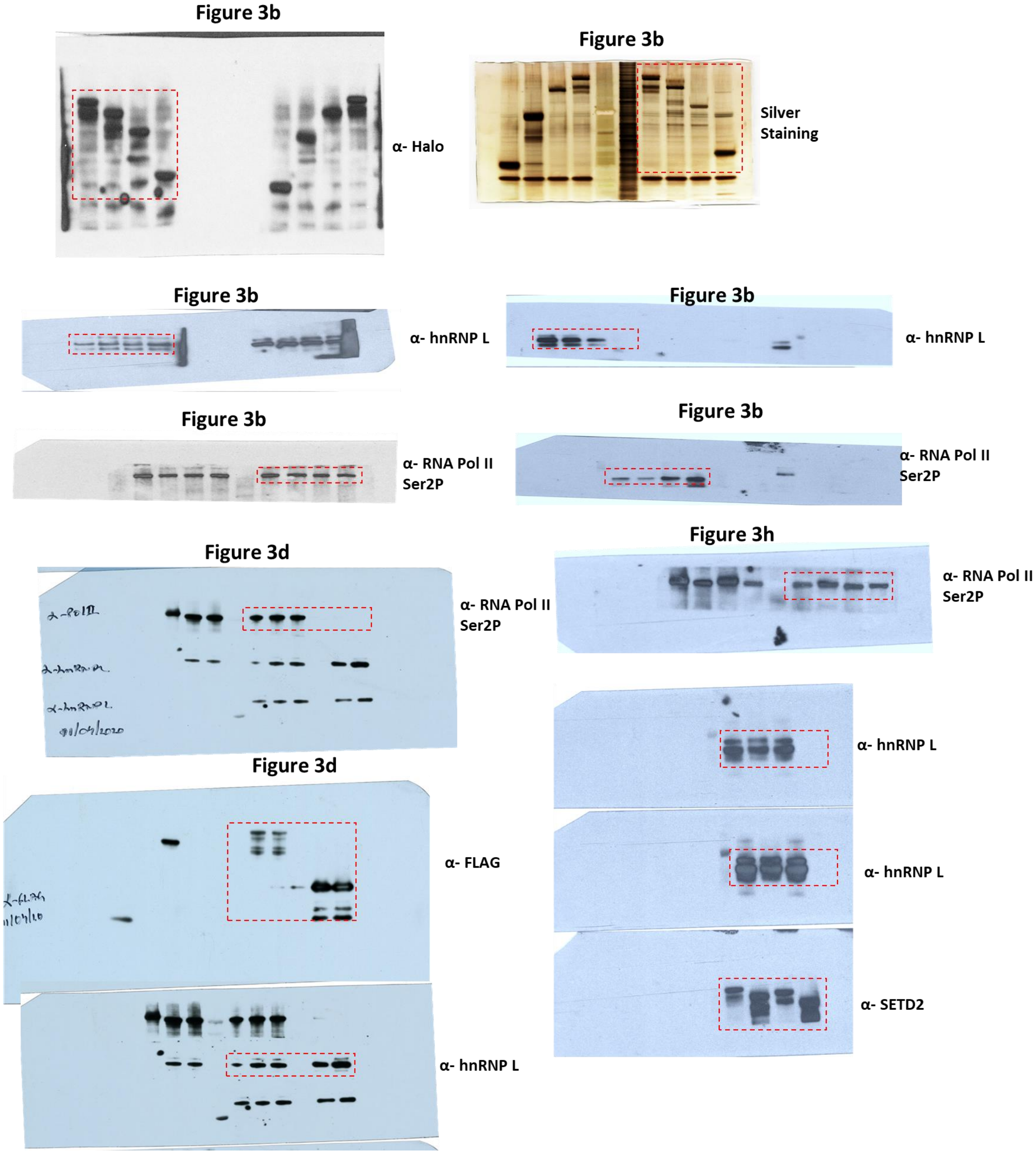
Figure 3b
α- Halo
Figure 3b
Silver Staining
Figure 3b
α- hnRNP L
Figure 3b
α- hnRNP L
Figure 3b
α- RNA Pol II Ser2P
Figure 3b
α- RNA Pol II Ser2P
Figure 3d
α- RNA Pol II Ser2P
Figure 3h
α- RNA Pol II Ser2P
Figure 3d
α- FLAG
α- hnRNP L
α- hnRNP L
α- hnRNP L
α- SETD2

Figure 4

Figure 4c

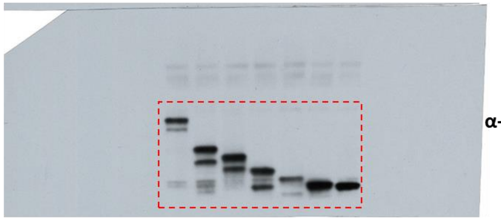

α- HA

Figure 4c

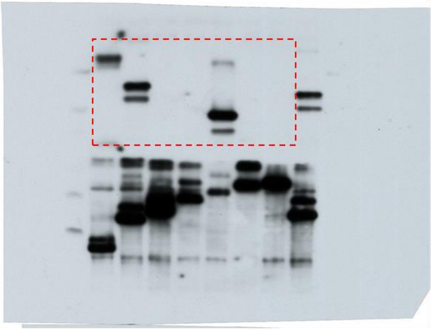

α- HA

Figure 4c

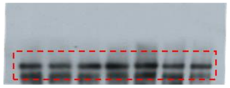

α- SETD2

Figure 4c

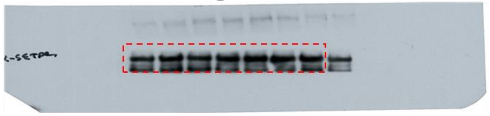

α- SETD2

Figure 4c

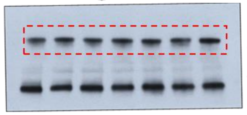

α- Pol II Ser2P

Figure 4c

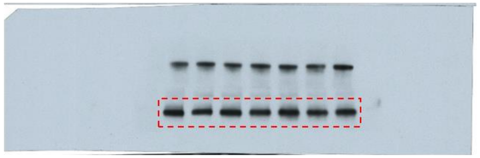

α- Pol II Ser2P

Figure 4c

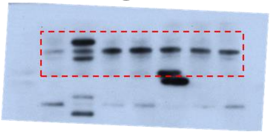

α- hnRNP L

Figure 4e

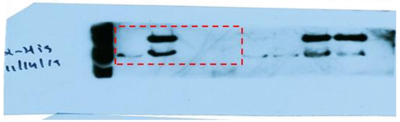

α- His

Figure 4c

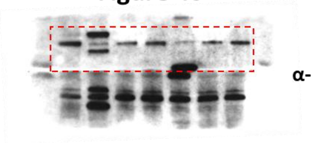

α- hnRNP L

Figure 4e

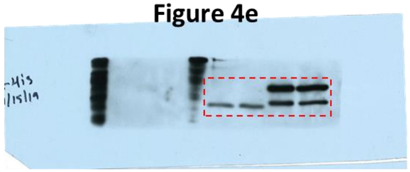

α- His

Figure 4e

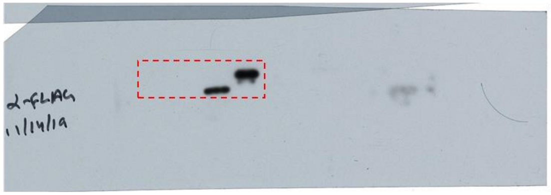

α- FLAG

Figure 4e

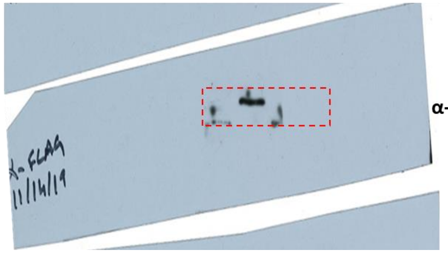

α- FLAG

Figure 6

Figure 6a

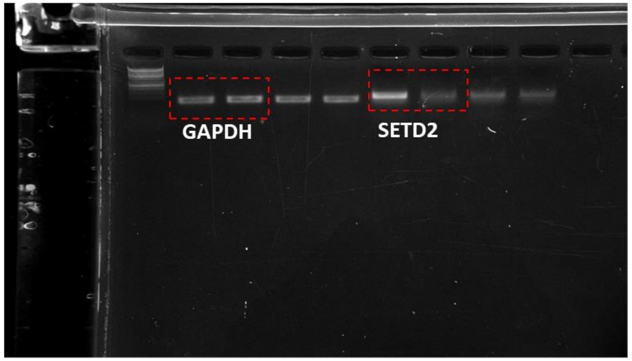

Figure 6a

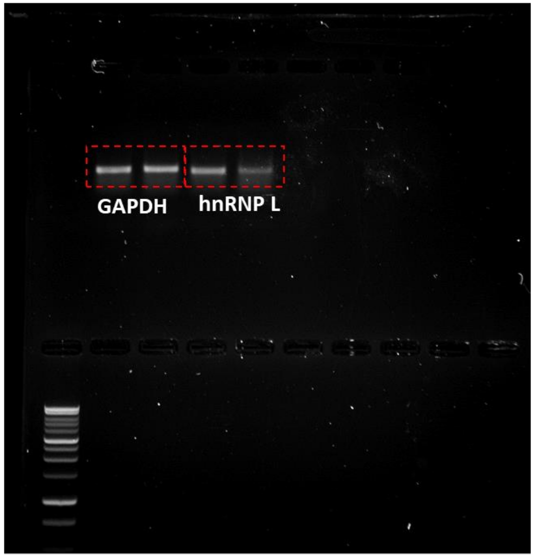

Figure 6a

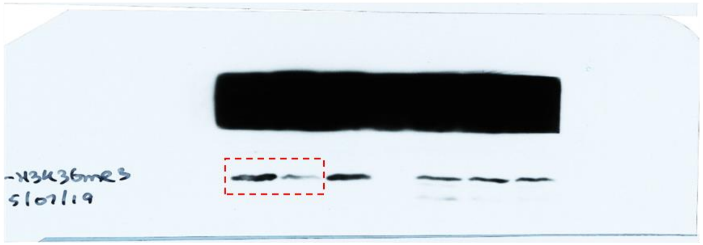

α-H3K36me3

Figure 6a

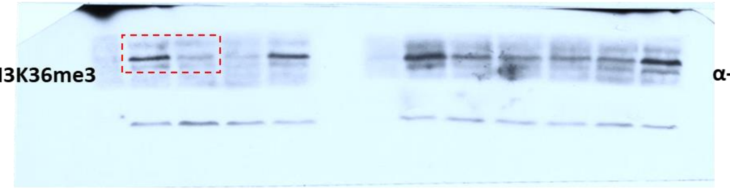

α-hnRNP L

Figure 6a

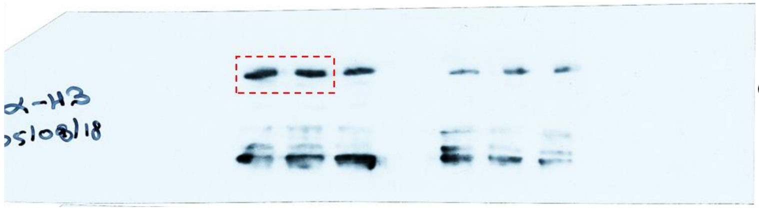

α-H3

Figure 6a

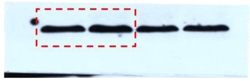

α-actin

Figure 7

Figure 7b

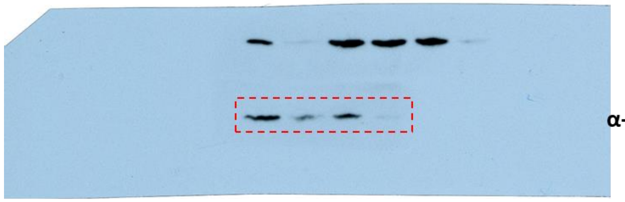

α-H3K36me3

Figure 7b

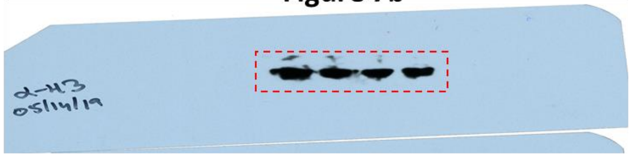

α-H3

Figure 7c

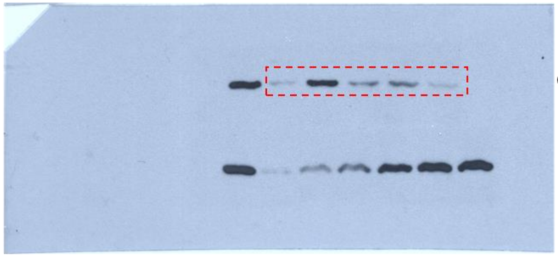

α-H3K36me3

Figure 7c

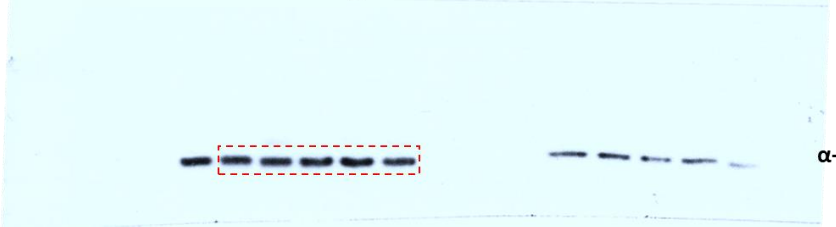

α-H3

Supplementary Figure 2

Supplementary Figure 2b

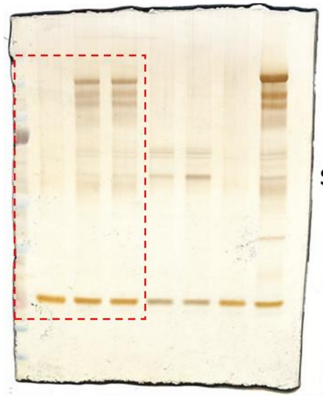

Silver Staining

Supplementary Figure 2c

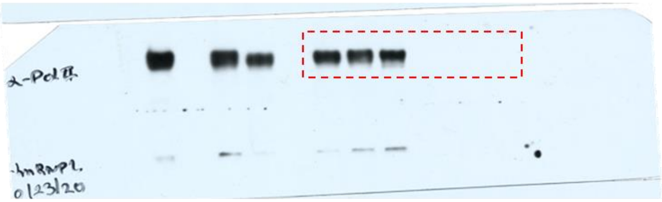

α- RNA Pol II Ser2P

Supplementary Figure 2c

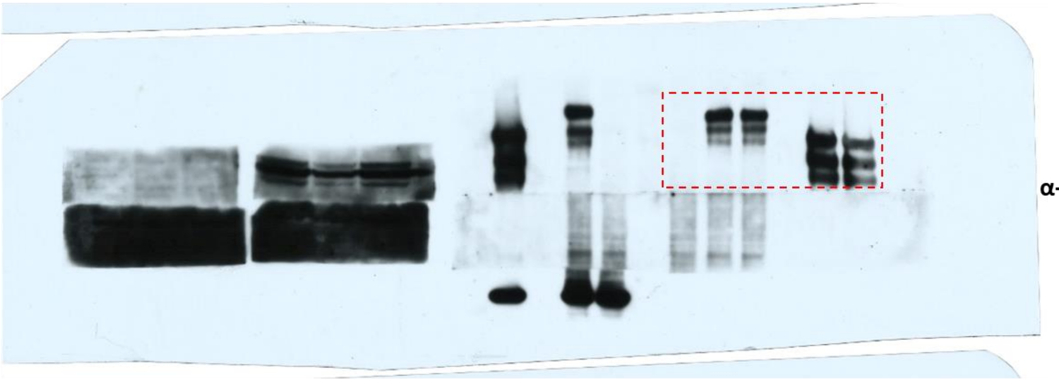

α-SETD2

Supplementary Figure 2c

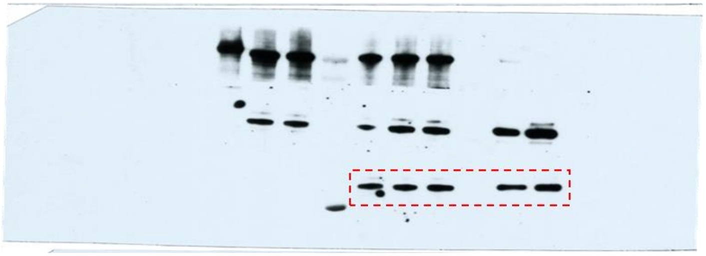

α-hnRNP L

Supplementary Figure 3

Supplementary Figure 3c

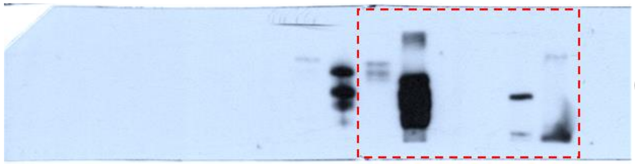

α-SETD2

Supplementary Figure 3c

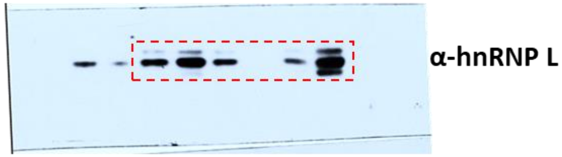

α-hnRNP L

Supplementary Figure 3c

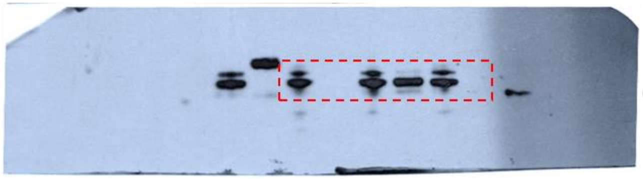

α-hnRNP L

Supplementary Figure 3d

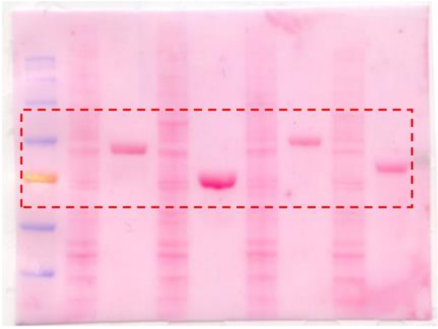

Ponceau

Supplementary Figure 3d

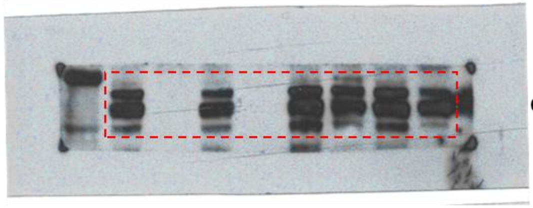

α-hnRNP L

Supplementary Figure 4

Supplementary Figure 4c

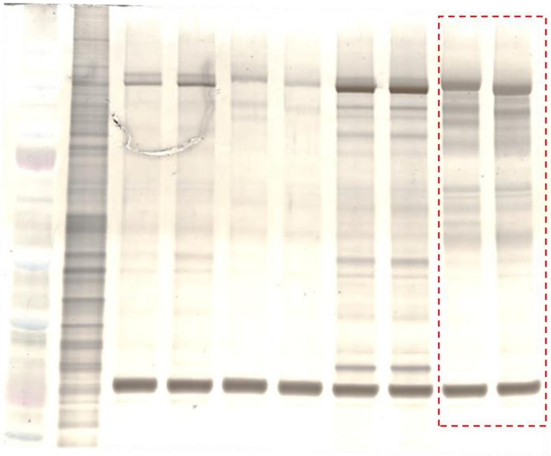

Silver Staining

Supplementary Figure 4d

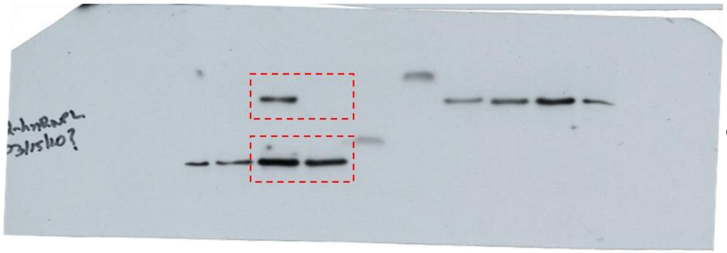

α-hnRNP L

Supplementary Figure 5

Supplementary Figure 5a

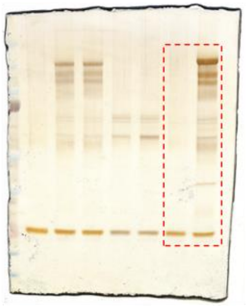

Silver Staining

Supplementary Figure 5b

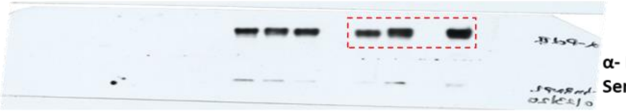

α- RNA Pol II Ser2P

Supplementary Figure 5b

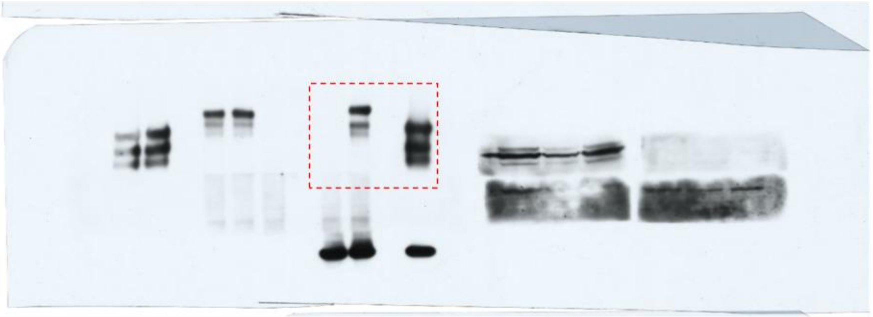

α-SETD2

Supplementary Figure 5b

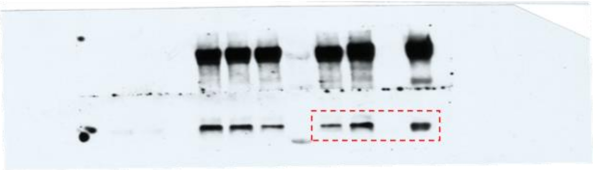

α-hnRNP L

Supplementary Figure 5b

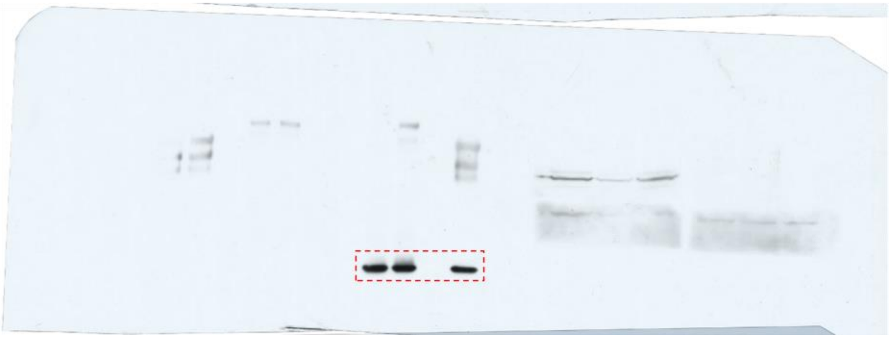

α-HA

Supplementary Figure 6

Supplementary Figure 6a

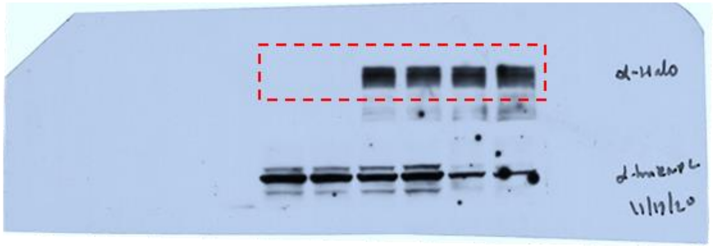

α- Halo

Supplementary Figure 6a

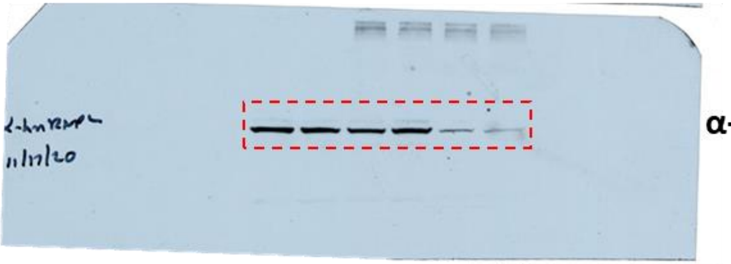

α- hnRNP L

Supplementary Figure 6a

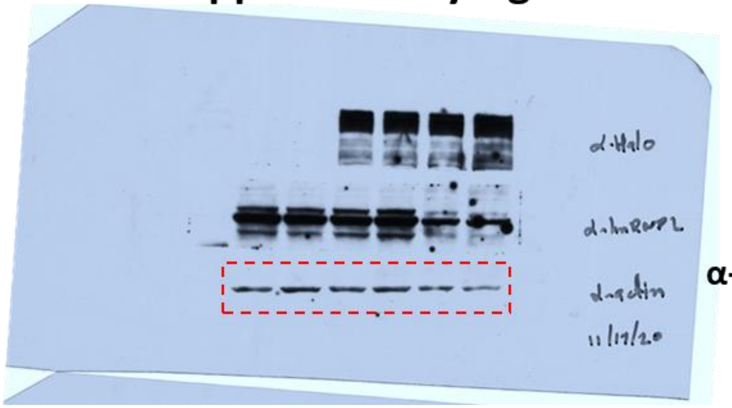

α- actin
