# Supplementary material for: The methyltransferase SETD2 couples transcription and splicing by engaging mRNA processing factors through its SHI domain
Source: Nat Commun. 2021 Mar 4;12:1443. doi: 10.1038/s41467-021-21663-w (PMC7933334; doi:10.1038/s41467-021-21663-w)
Supplement: Supplementary file 2 — Peer Review File [file 41467_2021_21663_MOESM2_ESM.pdf]

Reviewer #1 (Remarks to the Author):

The manuscript by Bhattacharya et al describes in great detail an interaction between hRNP L and SETD2. The work dissecting the interaction in very well done. This characterisation is followed by an analysis (RNA-Seq) of the effect of hRNP L or SETD2 depletion on mRNA levels and alternative splicing. The transcript affected by each depletion show some significant overlap, prompting the authors to conclude that the interaction they characterised is important for these processes. Finally, they show that the genes commonly affected by both depletions have particularly high levels of H3K36me3. These genomics analyses are rather superficial compared to the detailed biochemical dissection of the interaction that precede them. The effects and the overlaps are rather small. Overall, I am not convinced that these experiments establish a clear role for the interaction between SETD2 and hRNP L and splicing. I feel more direct evidence are needed to establish this link and for making that work suitable for a high-profile journal like Nature com.

Beyond, those general comments, I have a few specific suggestions that may help the authors improve their manuscript:

1- The authors show that many splicing factors beyond hRNP-L interact with SETD2 in a SHI domain-dependent manner. Are these other splicing factors interacting directly with the SHI domain or is hRNP L the main direct contact point recruiting other splicing factors. Purification in hRNP L k/d or CRISPR may help answer this question. Testing a few other splicing factors for direct interaction (recombinant) may also provide information.

2- The genes where splicing is co-regulated by SETD2 and hRNP L and show high K36me3 levels: are they genes more occupied by hRNP L or is hRNP L occupancy ubiquitous? hRNP L ChIP-seq would be useful.

3- The effect of deleting the SHI domain on bulk (WB) levels of H3K36me3 is very subtle. It needs to be repeated and quantified. Even better, the readout should be H3K36me3 ChIP-seq since one would expect the effect to be concentrated on genes co-regulated by hRNP L and SETD2. Here again, hRNP L ChIP-seq would be informative.

Reviewer #2 (Remarks to the Author):

This manuscript by Bhattacharya and colleagues describes physical and functional coupling of SETD2 and hnRNP L in controlling gene expression. This story builds on previous work that has suggested a functional interaction of these proteins, but has failed to provide solid evidence of both the interaction and the functional impact. Given the broad interest in coupling of splicing and transcription, definitive analysis of the interaction between SETD2 and hnRNP L has broad impact. The data presented are fully consistent with the conclusions and model proposed. However, in a few instances more work needs to be done to be fully definitive. In addition, more care should be taken in putting the current work in the context of the broader field.

Major comments:

1. In the fourth paragraph of the introduction it is fair to conclude that hnRNPs function in a context dependent manner, however it is not fair to conclude that hnRNPs do not have stringent RNA sequence specificity. Specifically, hnRNP L is well documented to require multiple contiguous CA dinucleotides – which are actually not overly ubiquitous (see Hui et al., EMBO 2005; Ray et al Nature 2013; Shankarling MBC 2014).

2. The authors conclude that the interaction of SETD2 and hnRNP L is independent of RNA and of interaction with Pol II, but these two conditions are not tested together (deltaSRI +RNase). Since it is

possible these are functionally redundant, this combination should be tested. Moreover, it is unclear whether RNase is included in the critical experiments mapping the interaction regions in SETD2 and hnRNP L (Fig 3 and 4) and "demonstrating" a direct interaction between these domains (Fig 4e). If RNase was not included in these experiments the key results need to be repeated in the presence of RNase.

3. The data in Figure 6 show a compelling correlation between the splicing impact of SETD2 and hnRNP L; however, to conclude that this is due to the interaction, the splicing of a few target genes should be tested upon rescue of the depleted cells with full-length vs. deltaSHI SETD2.

4. The text concludes that the SETD2 interaction with hnRNP L requires RRM2, however the constructs used also include ~50 amino acids downstream of RRM2. This is intriguing given previous data (Shankarling, Biochem J 2013) that the linker region between RRM2 and 3 in hnRNP L is required for full splicing repression activity. This should be mentioned in the manuscript, as it would be further consistent with the functional importance of the SETD2/hnRNP L interaction. Alternatively, if only sequences solely in RRM2 are required for interaction with SETD2, it would be interesting to determine how RRM2 differs from other RRMs in hnRNP L, and the similarity of RRM2 to RRMs in other hnRNPs identified in the MudPIT of SETD2.

Minor comment:
- There is an issue with the references, in that (1) commas or spaces are needed so that reference 2 and 3 doesn't look like ref 23 and (2) there are some issues in the listing of citations in the bibliography –e.g. see citations #8 and 10.

Reviewer #3 (Remarks to the Author):

General considerations

The main take home message of the manuscript by Battacharya et al. is that deposition of the H3K36me3 mark by the enzyme SETD2 maybe dependent on the physical interaction of SETD2 with the splicing factor hnRNPL. In this context, it is somewhat puzzling that the authors did not comment two seminal papers, published in 2011, by the groups of Maria Carmo Fonseca (de Almeida et al., NSMB) and David Bentley (Kim et al., PNAS) independently. The titles of both papers are sufficiently clear as to link them to the present work [Splicing enhances recruitment of methyltransferase HYPB/Setd2 and methylation of histone H3 Lys36 (doi: 10.1038/nsmb.2123.) and Pre-mRNA splicing is a determinant of histone H3K36 methylation (doi: 10.1073/pnas.1109475108)]. This said, in my view, the main claimed contribution of the present report is the identification of a specific interaction between an RNA recognition motif (RRM) of hnRNPL and an unforeseen domain of SETD2 that the authors denominate SHI. However, Fig. 5B clearly demonstrates that a full length SETD2 protein from which the SHI sequence was removed, not only loses interaction with hnRNPL but also with a panoply of at least 25 proteins involved in RNA processing, including the constitutive splicing factor U2AF2, the cleavage and polyadenylation factor CPCS7, and and the most conspicuous and well-studied hnRNPs and SR proteins such as hnRNPA1, SRSF1, 2 and 3. Since all mentioned proteins contain RRM domains, I would prefer to consider that SHI is an RRM-interacting domain rather than an hnRNPL-specific one. So, although it is commendable that the authors found SHI using hnRNPL, the whole paper is biased towards a key role of hnRNPL that might not be so, or at least shared by many other RRM-containing proteins. Proof of this is that, although there is some overlap between the alternative splicing (AS) events affected by hnRNPL and SETD2 depletions, its magnitude is small: only 213 out of 1221 events (17,4%) are correlated of which the vast majority are co-correlated (195) while 18 are anti-correlated.
The above general considerations together with the fact that the Reinberg lab showed that hnRNPL is

a "subunit" of SETD2 and is necessary for its activity in vivo but not in vitro (JBC 2009, reference 23) weaken the novelty of the main conclusions of this manuscript.

Specific comments

Experiments
Results

1. Mass-spec results in Fig. 1 are pushed too far. Why to pay attention to hnRNPL when thousands of proteins appear to interact with the hnRNPL 162-321 fragment and hnRNPL is far down in the list of Suppl. Table 1. I confess that I am not an expert in the analysis of mass-spec results, but the general readers of the journal will not be neither: is it not a SETD2 dNSAF of 0.0085 negligible compared to that of hnRNPL of 28.22 (Fig. 1f)? What would one expect for a near stoichiometric interaction? The same criticism applies to Fig. 1g, when full length hnRNPL is used as bait.

2. Fig. 1. A Western blot with antibodies to SETD2 should complement Fig. 1d.

3. Fig. 2c. The authors speculate that the abundance of hnRNPL interacting with SETD2C is so high that they see its band in the silver stained gel. However, the band they point as putative hnRNPL runs above the 58 kDa marker while in Fig. 1D we see that the apparent MW of hnRNPL 50 kDa. There are no detectable bands in the area of 50 kDa in Fig.2c. Again, a Western blot of the whole gel would solve the problem, that cannot be solved looking ast Fig. 2f because the authors only show cut stripes.

4. In any case, the dNSAF data shown in Fig. 2e seem to better support an interaction between SETD2C and hnRNPL (0.93 for the latter compared to 0.192 for the former). This confirms the weakness of Fig. 1 data.

5. Fig. 3c. The authors only show mass-spec results to support binding of hnRNPL to SETD2 fragments 1964-2564 and 2164-2213. I would like to see the corresponding Western blots that would be complementary, using the same methodology, of results in Fig. 3g, where a SETD2C fragment lacking the 2114-2263 segment is nor able to interact with hnRNPL.

6. Figures 5a and b are very informative and conclusive to compare the effects of removing the segment of SETD2 that interacts with Pol II (SRI) or the segment that interacts with "hnRNPL" (SHI). I've already mentioned in "General considerations" my interpretation of SHI as a pan-RRM interacting domain rather than being specific for hnRNPL.

7. Lines 288-299: "The depletion of the targets at the protein level was confirmed by...." What is the figure or table that supports this assertion?

7. Fig. 6. Commented in "general considerations".

Text and cited references

Introduction
- Lines 46-47: Splicing does not "determine the mRNA coding sequence" for two reasons: mRNAs contain 5' and 3' UTRs that do not code for protein and splicing also occurs in precursors of non-coding RNAs. I would replace "determine the mRNA coding sequence" by "yield mature RNAs".
- Reference 8 is not appropriate to illustrate coupling with splice site selection, i.e. AS, because it deals with budding yeast where AS is virtually not existing. For the kinetic model it would be better to cite either the original papers (de la Mata et al. Mol. Cell 2003; Dujardin et al. Mol.Cell 2014) or a review (Naftelberg et al. Annu. Rev. Biochem. 2015) from the Kornblihtt group.
- Lines 66-67: Why cytokine stimulation dependent manner is mentioned here at the same epistemic level as cell type and developmental stage? One could argue that a general feature of AS could be

“external cue dependent manner", which includes, apart from cytokines, every possible external signals like hormones, growth factors, temperature, UV irradiation, etc.
- Lines 72-73: “Combined with the fact that hnRNPs are very abundant and ubiquitous cellular proteins makes it unclear how the hnRNPs engage their target transcripts specifically”. What is the subject of this sentence?
- Lines 82-83: Please replace "specific interaction" by either "specific interactions" or " a specific interaction".

Results
Line 119: a reference is needed after the word "report".
Lines 172-173: "We were investigated which region..." The authors were not investigated.
Line 290: H3K36me3 methyltransferase should be replaced by H3K36 methyltransferase.
Line 407: A reference is needed after the word MRG15, which I suppose is Luco et al. Science 2010.

NCOMMS-20-28264

The histone methyltransferase SETD2 couples transcription and splicing by engaging pre-mRNA processing factors through its SHI domain

Saikat Bhattacharya, Michaella J. Levy, Ning Zhang, Hua Li, Laurence Florens, Michael P. Washburn and Jerry L. Workman

The authors would like to thank the reviewers for providing valuable feedback and suggestions to improve our manuscript, and the editors, for allowing us an opportunity to submit a revised version. The figures, their legends, and the manuscript text have been modified to incorporate the suggestions. The modified text has been highlighted in yellow in the revised manuscript.

REVIEWER COMMENTS

Reviewer #1 (Remarks to the Author):

The manuscript by Bhattacharya et al describes in great detail an interaction between hRNP L and SETD2. The work dissecting the interaction in very well done. This characterisation is followed by an analysis (RNA-Seq) of the effect of hRNP L or SETD2 depletion on mRNA levels and alternative splicing. The transcript affected by each depletion show some significant overlap, prompting the authors to conclude that the interaction they characterised is important for these processes. Finally, they show that the genes commonly affected by both depletions have particularly high levels of H3K36me3. These genomics analyses are rather superficial compared to the detailed biochemical dissection of the interaction that precede them. The effects and the overlaps are rather small. Overall, I am not convinced that these experiments establish a clear role for the interaction between SETD2 and hRNP L and splicing. I feel more direct evidence are needed to establish this link and for making that work suitable for a high-profile journal like Nature com.

- *The role of H3K36me3, which is deposited by SETD2, and hnRNP L is well known in the regulation of alternative splicing. Our poor knowledge of the specific interactome of hnRNPs has been a major impediment in the advancement of our understanding of the mode of action of the hnRNPs. Hence, in this manuscript, we have focused in detail on the meticulous characterization of the SETD2-hnRNP L interaction. By doing so, we have not only identified the specific domain of hnRNP L that binds to SETD2 but also have identified a novel domain in the SETD2 protein. The findings from our work are not only expected to pave a way for the identification of more specific interactions between SETD2, hnRNP L, and other proteins but also demonstrates that the interaction between hnRNP proteins and factors involved in transcription and epigenetic regulation can be mediated through very specific amino acid contacts. This has a broad impact on the field as Reviewer#2 suggested. The authors are confident that publication of this work in a highly reputed journal like Nature Communications will inspire research on hnRNPs which will subsequently help researchers in the field to elucidate mechanistic details behind the role of the context-dependent hnRNPs. We show that in addition to the SRI, the SHI domain is also required for SETD2 activity. This again is a major advancement of our understanding of the factors that regulate SETD2*

function. Moreover, the rescue experiments performed by us (Supplementary Figure 7 of the revised manuscript) provides strong evidence that the SHI domain-mediated interactions are required to rescue the common splicing defects observed upon SETD2 and hnRNP L depletion. The authors appreciate the constructive criticism by the reviewer and the good suggestions to improve our manuscript. We have performed experiments to address those concerns that are discussed below.

Beyond, those general comments, I have a few specific suggestions that may help the authors improve their manuscript:

1. The authors show that many splicing factors beyond hRNP-L interact with SETD2 in a SHI domain-dependent manner. Are these other splicing factors interacting directly with the SHI domain or is hRNP L the main direct contact point recruiting other splicing factors. Purification in hRNP L k/d or CRISPR may help answer this question. Testing a few other splicing factors for direct interaction (recombinant) may also provide information.

- *We would like to thank the reviewer for this valuable suggestion. Our data summarized in Figure 5b raises the interesting possibility that besides hnRNP L, SETD2 can interact with other proteins through its SHI domain. To test this possibility, we purified SETD2 from mammalian cell extracts after depleting hnRNP L. hnRNP L is a very abundant protein in cells and robustly interacts with SETD2. Hence, despite achieving a significant depletion of hnRNP L in cell extracts, a substantial amount of hnRNP L was present in SETD2C purification (sihnRNP L)* ***[Figure 1a, b]****. Despite this, we found that not only did the binding of SETD2 with other splicing related proteins persisted upon the depletion of hnRNP L, strikingly, their binding increased relative to the purifications from extracts of scramble-siRNA (siScramble) treated cells* ***[Figure 1b]****. This is precisely what would be expected to happen if the other proteins also engage with SETD2 through its SHI domain where hnRNP L binds. Notably, the purification of SETD2 from extracts depleted of hnRNP L revealed enrichment of hnRNP A1. On the other hand, the enrichment of U2AF2 was reduced* ***[Figure 1b]****. Possibly, SETD2 can recruit spliceosomes by more than one mechanism. SETD2 by interacting with hnRNP L might result in the recruitment of proteins such as U2AF2. SETD2 might also engage the core hnRNP proteins which in turn can bring other splicing factors for the formation of the spliceosome to dictate the AS outcome. This data has been added as Supplementary Figure 6 and Supplementary Table 6 of the revised manuscript and appropriate discussion has been included. As the recombinant*

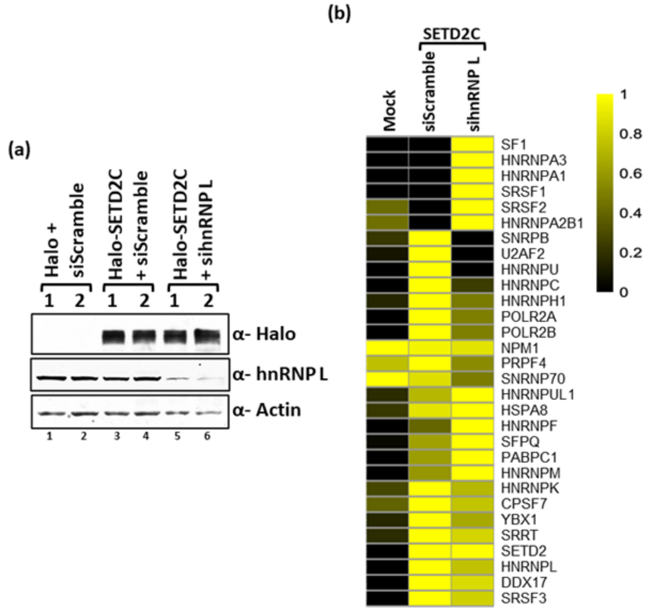

Figure 1. (a) Western blotting of whole cell extracts of 293T cells that were used for Halo purification followed by MudPIT. (b) Heat map showing the enrichment of proteins in MudPIT analysis.

version of the full-length hnRNP proteins are often insoluble and sticky in nature, more information of their specific binding region is required to perform in vitro binding assays. In the future, a detailed characterization of these possible interactions will be performed to establish their binding to SETD2. Structural studies are underway in our group to determine how the SETD2 SHI and the hnRNP L RRM2 domains interact.

In the Discussion section of our initial submission, we have discussed a few possibilities as to why the overlap between SETD2 and hnRNP L regulated splicing events are small much like what was observed for Med23 and hnRNP L by Huang, Y. et al, 2012. In addition to those, the results discussed above strongly indicate that SETD2 engages other proteins involved in pre-mRNA splicing besides hnRNP L, which might be the reason behind the small overlap. This possibility also has now been discussed in the revised manuscript.

2. The genes where splicing is co-regulated by SETD2 and hRNP L and show high K36me3 levels: are they genes more occupied by hRNP L or is hRNP L occupancy ubiquitous? hRNP L ChIP-seq would be useful.

- *We analyzed the publicly available hnRNP L and H3K36me3 ChIP data to see whether a pattern emerges between hnRNP L binding and H3K36me3 distribution:*

Cell Line: HEPG2
hnRNP L ChIP: Encode ENCSR315JJE
H3K36me3 ChIP: Encode ENCSR000AMB

Cell Line: HELA
hnRNP L ChIP: GEO GSE33887
H3K36me3 ChIP: GEO GSE33887

*We first confirmed that in both these cell lines SETD2 and hnRNP L interact by performing affinity purification of Halo-SETD2C and probing the purified complexes with anti-hnRNP L antibody **[Figure 2]**.*

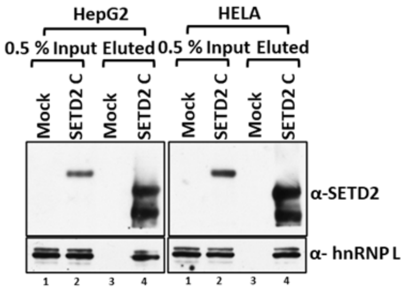

Figure 2. Halo purification was performed from extracts of HepG2 and Hela cells expressing Halo-SETD2C. Input and eluted samples were resolved on a gel followed by western blotting with the antibodies depicted.

*In both these epithelial cell lines, a strong correlation could not be found between hnRNP L binding and H3K36me3 distribution irrespective of whether gene bodies **[Figure 3]** or promoters were analyzed.*

*This is consistent with our data in which a correlation was not observed between H3K36me3 level and hnRNP L-regulated AS genes **[Figure 4]**.*

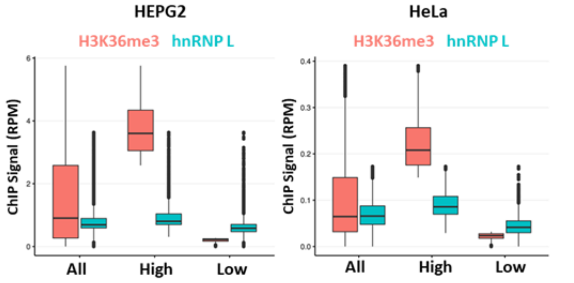

Figure 3. Boxplots depicting the distribution of H3K36me3. The High group contains the top 25% protein coding genes with the highest H3K36me3 signals, and the Low group contains the bottom 25% protein coding genes with the lowest (but positive) H3K36me3 signals.

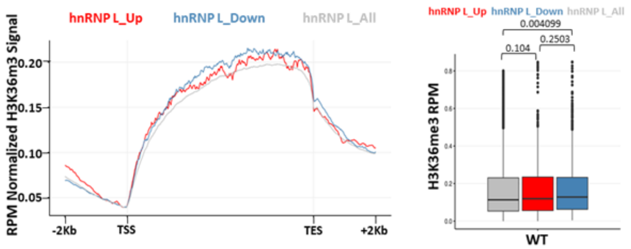

Figure 4. Metagene plot and boxplot depicting the distribution of H3K36me3 of genes that show differential alternative splicing upon hnRNP L depletion. p-values are shown on the box plots.

Next, we checked whether hnRNP L is ChIPed more at its known target genes. For this, we looked at previously reported hnRNP L targets in HeLa cells, namely, CD55 (DAF), TJP1, PAPOLA, LIFR, and ASAH1 (Hung et al., 2008) that we also found to be aberrantly spliced in 293T cells. Noticeably, hnRNP L was not found to be enriched even at its known target genes **[Figure 5]**.

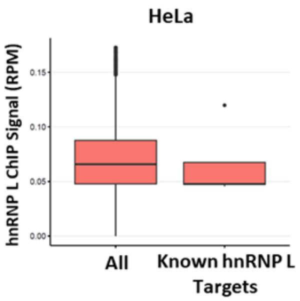

Figure 5. Boxplots depicting overall ChIP signal of hnRNP L vs signal on known hnRNP L target genes.

These datasets also revealed a binding pattern that suggests ubiquitous hnRNP L occupancy unlike that observed for H3K36me3 which is mainly restricted to gene bodies **[Figure 6]**.

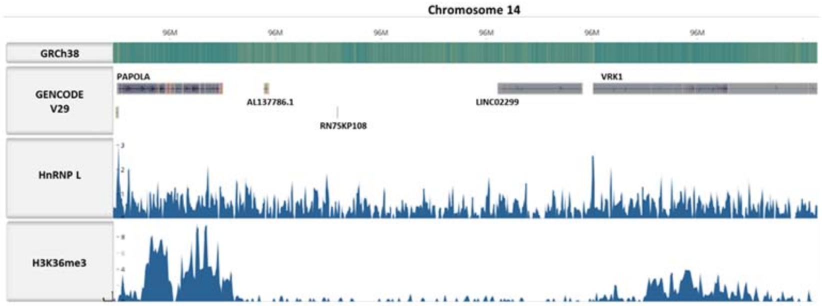

Figure 6. Genome browser view of hnRNP L and H3K36me3 ChIP-Seq tracks.

It must be noted though that hnRNP L does not have a known DNA binding domain and hence, it cannot be ruled out, that the high abundance of hnRNP L may allow it to cross-link to chromatin promiscuously. HnRNPs are one of the most abundant and ubiquitous proteins present in the cells which makes it likely that they are present in vast excess over their respective binding sites. Finally, from our own experience of working with them, some hnRNP proteins can be "sticky" to work with biochemically.

As hnRNP L is known to engage RNA, we tried performing RIP-Seq of hnRNP L using a commercially available kit from Active Motif (RNA ChIP-IT® Kit #53024). The hnRNPs share sequence similarities and their RRMs can be very similar too. Hence, it is critical to use antibody specific for a given hnRNP, especially in experiments like ChIP or RIP to draw meaningful conclusions. In our experiments, we used CST #37562 hnRNP L antibody. The exact antigen sequences of CST antibody are proprietary; however, we were informed by CST that #37562 hnRNP L antibody is produced by immunizing animals with a synthetic peptide corresponding to residues surrounding His313 of human hnRNP L protein. Based on BLAST alignment results, there is a high likelihood that this antibody might be specific for hnRNP L. The Product Usage page of CST does not list ChIP and RIP as applications for this antibody. Unfortunately, our multiple attempts to perform hnRNP L ChIP- and RIP-Seq did not yield much success. Upon sequencing of the ChIPed DNA, specific enrichment of hnRNP L was not observed although more than 25 million reads aligned to the genome. Library preparation did not succeed in our RIP experiments. Notably, RIP of the positive control Suz12 (supplied by Active Motif RNA ChIP-IT® Control Kit – Human #53025) worked as expected and a clear enrichment was observed at the SFPQ locus in qRT-PCR experiments **[Figure 7]**. It is likely that the antibodies against hnRNP L used by us

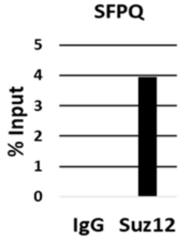

Figure 7. Bar graph showing an enrichment of Suz12 at the SFPQ locus in RIP experiment by qRT-PCR.

were not RIP-grade, resulting in our failure to pull down hnRNP L effectively. The possibility of a promiscuous cross-linking of hnRNP L to chromatin makes it an unsuitable candidate for performing epitope-tagged ChIP.

Analysis of the publicly available ChIP-Seq datasets reveals the complexity of deciphering the mode of action of hnRNPs. Moreover, the rescue experiments performed by us (Supplementary Figure 8 of the revised manuscript) provides strong evidence that the SHI domain-mediated interactions are required to rescue the common splicing defects observed upon SETD2 and hnRNP L depletion. In this experiment setd2Δ 293T cells were rescued with Vector Control, SETD2 FL and SETD2 FLΔSHI construct to test the splicing of few target genes the splicing of which was decreased upon SETD2 and hnRNP L depletion. Individual alternative splicing events were measured by quantitative PCR and represented by the ratios of the intron to an exon, or different exons. Indeed, the rescue of setd2Δ 293T cells with SETD2 FL led to an increase in the ratio as compared to the vector control. No change in splicing was observed upon expression of SETD2 FLΔSHI relative to the control ***[Figure 8]***.

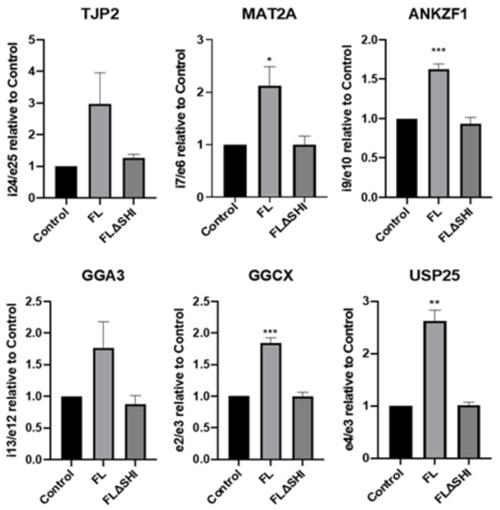

Figure 8. *setd2Δ* 293T cells were rescued with Vector Control, SETD2 FL and SETD2 FLΔSHI constructs and their RNA was isolated 72 hours post-transfection. Specific primers were designed to detect the indicated introns and exons and individual alternative splicing events were measured by quantitative PCR and represented by the ratios of intron to exon, or different exons. Standard Error (SE) is depicted. p-value <0.05 was considered significant.

The mechanism of how hnRNPs regulate RNA processing is poorly understood which partly has resulted from our poor knowledge of the specific interactome of hnRNPs. The inability to differentiate proteins that may be adventitiously bound to hnRNP complexes from authentic interactors has been an issue due to the heavy nucleic acid contamination and the RNA binding nature of the hnRNPs. Our work demonstrates specific interaction between SETD2 and hnRNP L and the domains involved in the interaction. The demonstration of the functional interaction between SETD2 and hnRNP L is an important milestone towards our path to understanding how the crosstalk between transcription and splicing machinery occurs.

3. The effect of deleting the SHI domain on bulk (WB) levels of H3K36me3 is very subtle. It needs to be repeated and quantified. Even better, the readout should be H3K36me3 ChIP-seq since one would expect the effect to be concentrated on genes co-regulated by hRNP L and SETD2. Here again, hRNP L ChIP-seq would be informative.

- *As per the reviewer's suggestion, western blot data for bulk levels of H3K36me3 from biological replicates were quantified and plotted* ***[Figure 9]****. The deletion of the SHI domain results in a reproducible and significant decrease in global H3K36me3. Also, the double mutant*

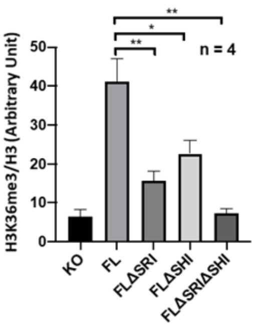

Figure 9. Bar graph showing H3 normalized H3K36me3 signal intensity. The data plotted are from four independent biological replicates. p-value < 0.05 was considered significant. Error bars are for Standard Error.

lacking both the SHI and SRI domains almost completely loses activity.
Emerging evidence suggests that factors such as Pol II (Wang et al., 2015), and H3.3S31ph (Armache et al., 2020) are global activators of SETD2's in vivo methyltransferase activity. The engagement of Set2 and Pol II through the SRI domain is required for the activation of Set2 instead of its chromatin recruitment (Wang et al., 2015). Consistent with this, we recently showed that at a high expression level, SETD2 has a reduced dependency on such activators and can deposit H3K36me3 even without RNA Pol II association (Bhattacharya and Workman, 2020). Considering this evidence and the decrease in H3K36me3 level seen in western blotting, we anticipated a global decrease in H3K36me3 upon deletion of the SRI and SHI domains.
*To test this, we introduced SETD2 FL, FLΔSRI, and FLΔSHI in setd2Δ 293T cells and performed spike-in normalized H3K36me3 ChIP-Seq analysis. Analysis of the ChIP-Seq data showed a global decrease in H3K36me3 deposition in cells rescued with SETD2 mutants as compared to SETD2 WT **[Figure 10]**, corroborating our western blot results. Also, a closer inspection reveals that the loss occurs from both high and low expressed genes, consistent with the idea that both Pol II and hnRNP L are global regulators of SETD2's in vivo activity **[Figure 10]**.*

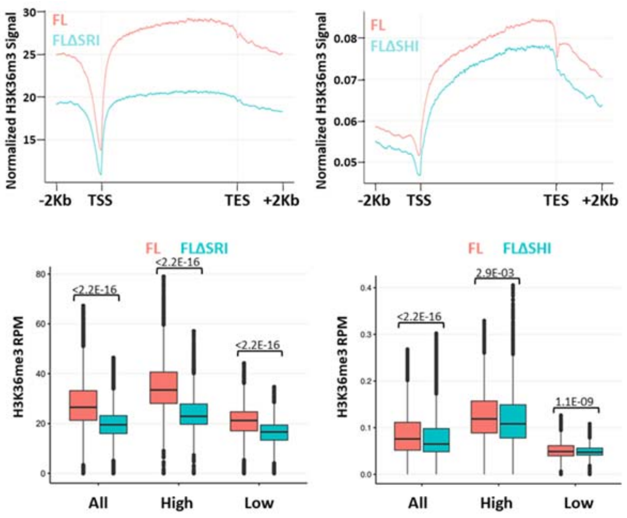

Figure 10. Metagene plot and boxplot depicting the distribution of H3K36me3 upon expression of SETD2 FL, FLΔSRI, and FLΔSHI in setd2Δ 293T cells. p-values are depicted on the box plots.

*Next, we looked specifically at H3K36me3 levels at those genes where the splicing was co-regulated by SETD2 and hnRNP L. In SETD2 FLΔSHI expressing cells, the pattern was similar to that observed in SETD2 FL expressing and WT cells, again suggesting that the SHI domain-mediated activation of SETD2 is not specific to a given set of genes but occurs globally as seen in the analysis above **[Figure 11]**.*
The results discussed above have been incorporated in Figure 7 and Supplementary Figure 10 of the revised manuscript. The appropriate discussion has been added in the Discussion section.

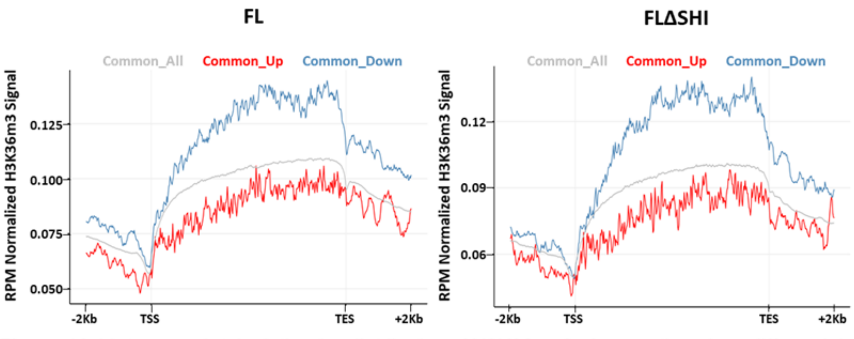

Figure 11. Metagene plot depicting the distribution of H3K36me3 of genes that show differential alternative splicing upon SETD2 and hnRNP L depletion after rescuing *setd2Δ* 293T cells SETD2 FL and SETD2 FLΔSHI.

Reviewer #2 (Remarks to the Author):

This manuscript by Bhattacharya and colleagues describes physical and functional coupling of SETD2 and hnRNP L in controlling gene expression. This story builds on previous work that has suggested a functional interaction of these proteins, but has failed to provide solid evidence of both the interaction and the functional impact. Given the broad interest in coupling of splicing and transcription, definitive analysis of the interaction between SETD2 and hnRNP L has broad impact. The data presented are fully consistent with the conclusions and model proposed. However, in a few instances more work needs to be done to be fully definitive. In addition, more care should be taken in putting the current work in the context of the broader field.

- *The authors would like to thank the reviewer for the positive remarks and constructive suggestions. These have helped us in strengthening our work and improving the overall quality of our manuscript.*

Major comments:

1. In the fourth paragraph of the introduction it is fair to conclude that hnRNPs function in a context dependent manner, however it is not fair to conclude that hnRNPs do not have stringent RNA sequence specificity. Specifically, hnRNP L is well documented to require multiple contiguous CA dinucleotides – which are actually not overly ubiquitous (see Hui et al., EMBO 2005; Ray et al Nature 2013; Shankarling MBC 2014).

- *The point by the reviewer is well taken. The CA-rich regulatory elements that regulate alternative splicing although widespread are not ubiquitous. We have modified the introduction appropriately to highlight the possibility that there might be additional factors that determine the hnRNPs target specificity.*

2. The authors conclude that the interaction of SETD2 and hnRNP L is independent of RNA and of interaction with Pol II, but these two conditions are not tested together (deltaSRI +RNase). Since it is possible these are functionally redundant, this combination should be tested. Moreover, it is unclear whether RNase is included in the critical experiments mapping the interaction regions in SETD2 and hnRNP L (Fig 3 and 4) and “demonstrating” a direct interaction between these domains (Fig 4e). If RNase was not included in these experiments the key results need to be repeated in the presence of RNase.

- *As per the reviewer’s suggestion, we tested the CΔSRI + RNase combination. hnRNP L co-purifies with SETD2 even under these conditions **[Figure 12]**. This data has been included in Supplementary Figure 2 of the revised manuscript.*

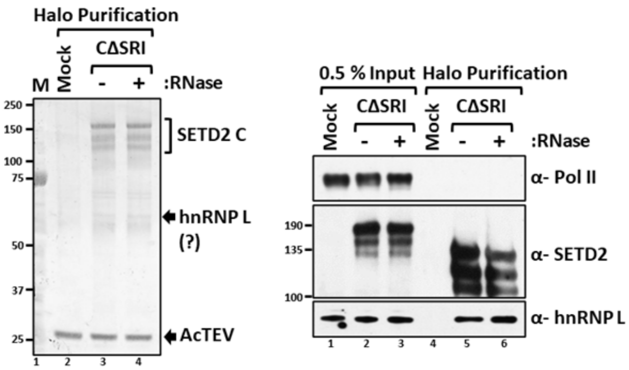

Figure 12. Halo purification was performed from extracts of 293T cells expressing Halo-SETD2CΔSRI. Input and eluted samples were resolved on a gel followed by silver staining or western blotting with the antibodies depicted.

- *RNase was included in the experiment for Figure 4e. However, RNase was not included in the experiments for Figure 3 and Figure 4c. This has now been mentioned clearly in the figure legends.*
- *Also, as per the reviewer's suggestion, we have repeated the key result in the presence of RNase, and it demonstrates the co-purification of hnRNP L **[Figure 13]**. This data has been included in Figure 3 of the revised manuscript.*

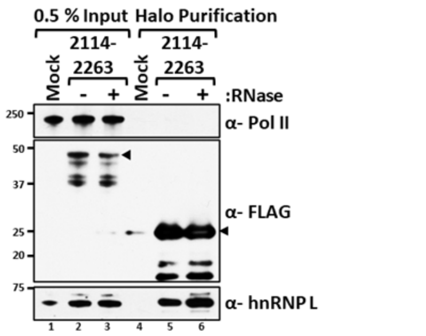

Figure 13. Halo purification was performed from extracts of 293T cells expressing Halo FLAG-tagged SETD2 2114-2263. Input and eluted samples were resolved on gel followed by western blotting.

3. The data in Figure 6 show a compelling correlation between the splicing impact of SETD2 and hnRNP L; however, to conclude that this is due to the interaction, the splicing of a few target genes should be tested upon rescue of the depleted cells with full-length vs. deltaSHI SETD2.

- *We would like to thank the reviewer for suggesting this useful experiment. Consequently, setd2Δ 293T cells were rescued with Vector Control, SETD2 FL and SETD2 FLΔSHI construct to test the splicing of few target genes the splicing of which was decreased upon SETD2 and hnRNP L depletion. Individual alternative splicing events were measured by quantitative PCR and represented by the ratios of the intron to an exon, or different exons. Indeed, the rescue of setd2Δ 293T cells with SETD2 FL led to an increase in the ratio as compared to the vector control. No change in splicing was observed upon expression of SETD2 FLΔSHI relative to the control **[Figure 8]**. This data has been included in Supplementary Figure 8 of the revised manuscript.*

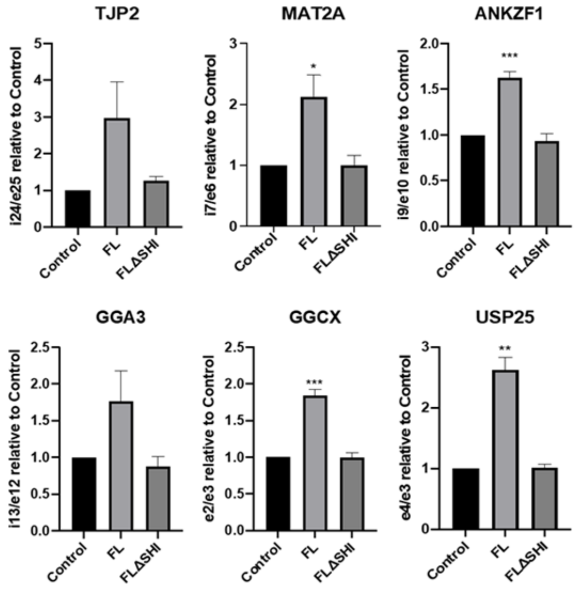

Figure 8. *setd2Δ* 293T cells were rescued with Vector Control, SETD2 FL and SETD2 FLΔSHI constructs and their RNA was isolated 72 hours post-transfection. Specific primers were designed to detect the indicated introns and exons and individual alternative splicing events were measured by quantitative PCR and represented by the ratios of intron to exon, or different exons. Standard Error (SE) is depicted. p-value <0.05 was considered significant.

4. The text concludes that the SETD2 interaction with hnRNP L requires RRM2, however the constructs used also include ~50 amino acids downstream of RRM2. This is intriguing given previous data (Shankarling, Biochem J 2013) that the linker region between RRM2 and 3 in hnRNP L is required for full splicing repression activity. This should be mentioned in the manuscript, as it would be further consistent with the functional importance of the SETD2/hnRNP L interaction. Alternatively, if only sequences solely in RRM2 are required for interaction with SETD2, it would be interesting to determine how RRM2 differs from other

RRMs in hnRNP L, and the similarity of RRM2 to RRMs in other hnRNPs identified in the MudPIT of SETD2.

- *The reviewer correctly pointed out that the hnRNP L 162-321 construct used in our experiments includes amino acids downstream of the RRM2. To test whether just the RRM2 of hnRNP L can interact with SETD2, we performed a co-IP experiment of hnRNP L 189-293 with SETD2. The purification demonstrates that only the RRM2 of hnRNP L is sufficient to interact with SETD2* ***[Figure 14]****. This data has been included as Supplementary Figure 5 in the revised manuscript.*

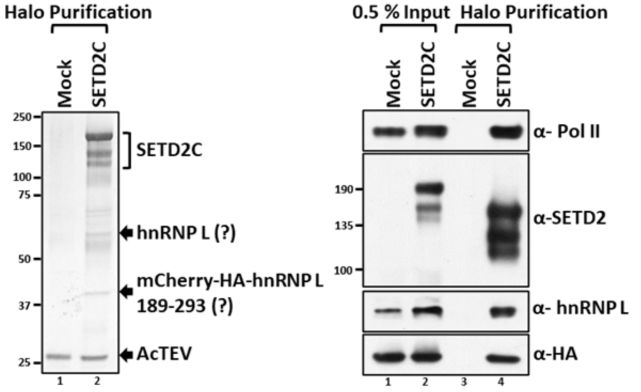

Figure 14. Halo purification was performed from extracts of 293T cells co-expressing Halo-SETD2C and mCherry-HA-hnRNP L 189-293. Input and eluted samples were resolved on a gel followed by silver staining or western blotting with the antibodies depicted.

We have performed sequence alignment of the RRMs of hnRNP L. However, in the absence of structural insights, it is difficult to speculate why only the RRM2 can interact with SETD2 and not the others ***[Figure 15]****. The sequence alignment has been included as Supplementary Figure 5 in the revised manuscript and a few lines have been added in the Discussion section. Based on the new experiments that we have performed (Supplementary Figure 6 of the revised manuscript), it is likely that besides hnRNP L, other RNA processing factors might also interact with SETD2. Structural studies are underway in our group to determine how the SETD2 SHI and the hnRNP L RRM2 domains interact. Once we have more information on that, as the reviewer suggested, it will be very interesting to determine the basis of the specificity of hnRNP L RRM2-SETD2 interaction and what other hnRNPs might have similar RRMs that might allow them to interact with SETD2.*

```
RRM4       --------------------RIQHPSNVLHFFN-APLEVTEENFFEICDELGVKRPSSVK  39
RRM2       ---------------------RSVNSVLLFTILNPIYSITTDVLYTICNPCGPVQR--IV  37
RRM1       -------------------------SPVVHIRGLIDGVVEADLV-EALQEFGPISY--VV  32
RRM3       PSRYGPQYGHPPPPPPPPEYGPHADSPVLMVYGLDQSKMNCDRVFNVFCLYGNVEK--VK  58
                                    * ::         :  : .       *      :

RRM4       VFSGKSERSSSGLLEWESKSDALETLGFLNHYQMKNPNGPYPYTLKLCF-----------  88
RRM2       IFRKN---GVQAMVEFDSVQSAQRAKASLNGA------DIYSGCCTLKIE---YAKPTRL  85
RRM1       VMPKK----RQALVEFEDVLGACNAVNYAADN-------------QIYIA----------  65
RRM3       FMKSK---PGAAMVEMADGYAVDRAITHLNNN------FMFGQKLNVCVSKQPAIMPGQS  109
           .:  :      .::*  .   . .:                     : .

RRM4       -------------------   88
RRM2       NVFK-NDQDTWDYTNP---   100
RRM1       -----GHPAFVNYSTSQKI   79
RRM3       YGLEDGSCSYKDFSES---   125
```

Figure 15. Clustal Omega (https://www.ebi.ac.uk/Tools/msa/clustalo/) sequence alignment of hnRNP L RRMs.

Minor comment:

1. There is an issue with the references, in that (1) commas or spaces are needed so that reference 2 and 3 doesn't look like ref 23 and (2) there are some issues in the listing of citations in the bibliography –e.g. see citations #8 and 10.

- *The authors would like to thank the reviewer for pointing out the issues with the references. The citation style has been corrected.*

Reviewer #3 (Remarks to the Author):

General considerations

The main take home message of the manuscript by Battacharya et al. is that deposition of the H3K36me3 mark by the enzyme SETD2 maybe dependent on the physical interaction of SETD2 with the splicing factor hnRNPL. In this context, it is somewhat puzzling that the authors did not comment two seminal papers, published in 2011, by the groups of Maria Carmo Fonseca (de Almeida et al., NSMB) and David Bentley (Kim et al., PNAS) independently. The titles of both papers are sufficiently clear as to link them to the present work [Splicing enhances recruitment of methyltransferase HYPB/Setd2 and methylation of histone H3 Lys36 (doi: 10.1038/nsmb.2123.) and Pre-mRNA splicing is a determinant of histone H3K36 methylation (doi: 10.1073/pnas.1109475108)]. This said, in my view, the main claimed contribution of the present report is the identification of a specific interaction between an RNA recognition motif (RRM) of hnRNPL and an unforeseen domain of SETD2 that the authors denominate SHI. However, Fig. 5B clearly demonstrates that a full length SETD2 protein from which the SHI sequence was removed, not only loses interaction with hnRNPL but also with a panoply of at least 25 proteins involved in RNA processing, including the constitutive splicing factor U2AF2, the cleavage and polyadenylation factor CPCS7, and and the most conspicuous and well-studied hnRNPs and SR proteins such as hnRNPA1, SRSF1, 2 and 3. Since all mentioned proteins contain RRM domains, I would prefer to consider that SHI is an RRM-interacting domain rather than an hnRNPL-specific one. So, although it is commendable that the authors found SHI using hnRNPL, the whole paper is biased towards a key role of hnRNPL that might not be so, or at least shared by many other RRM-containing proteins. Proof of this is that, although there is some overlap between the alternative splicing (AS) events affected by hnRNPL and SETD2 depletions, its magnitude is small: only 213 out of 1221 events (17,4%) are
correlated of which the vast majority are co-correlated (195) while 18 are anti-correlated.
The above general considerations together with the fact that the Reinberg lab showed that hnRNPL is a "subunit" of SETD2 and is necessary for its activity in vivo but not in vitro (JBC 2009, reference 23) weaken the novelty of the main conclusions of this manuscript.

- *The authors would like to thank the reviewer for constructive criticism and pointing out the necessary corrections that are required to improve our manuscript. We have included the data in the revised manuscript that the reviewer asked for and have provided clarifications for the concerns raised. Please see the detailed response below.*

Specific comments

Experiments
Results

1. Mass-spec results in Fig. 1 are pushed too far. Why to pay attention to hnRNPL when thousands of proteins appear to interact with the hnRNPL 162-321 fragment and hnRNPL is far down in the list of Suppl. Table 1.

- *We assume that the reviewer is referring to our focus on SETD2 rather than hnRNPL in the aforementioned figure and respond below.*
  We chose to focus on SETD2 because, although there were hundreds to a thousand proteins identified with the hnRNP L 162-321 fragment, only 25 were pulled down by both +/- RNase samples that were not identified in the mock control. Of these 25 proteins, SETD2 was of particular interest to do additional experiments because it engages the C-terminal domain (CTD) of the elongating RNA Pol II which makes it a good candidate to bridge the splicing apparatus and the transcription elongation machinery. Furthermore, much like for hnRNP L, SETD2 depletion is known to result in splicing aberrations. These have been mentioned in the Results section. Also, it has been mentioned in the Introduction section that hnRNP L has been shown to co-purify SETD2 although, the functional relevance of this interaction was not clear. We have added a line of text in the revised manuscript clarifying our choice to highlight SETD2 from this list of several hundred proteins.

2. I confess that I am not an expert in the analysis of mass-spec results, but the general readers of the journal will not be neither: is it not a SETD2 dNSAF of 0.0085 negligible compared to that of hnRNPL of 28.22 (Fig. 1f)? What would one expect for a near stoichiometric interaction? The same criticism applies to Fig. 1g, when full length hnRNPL is used as bait.

- *The authors recognize the reviewer's concern that a person not acquainted in interpreting mass spectrometry data may find the tabulated dNSAF values of SETD2 in Figure 1 very low for meaningful consideration. The low dNSAF values of SETD2 can be attributed to various factors:*

1. *Previously, we and others have shown that the full-length SETD2 protein is robustly degraded by the ubiquitin-proteasome pathway (Bhattacharya and Workman, 2020) (Zhu et al, 2016) (Dronamraju et al, 2018). The low dNSAF values of SETD2 could be due to the very low abundance of the endogenous SETD2 protein. A line has been added in the text of the revised manuscript to make the readers acquainted with this information.*
2. *For a near stoichiometric interaction, the ratio of dNSAFs should be close to one. However, for that to happen both the bait and prey should be present in similar abundance and there should be no competing factors affecting their binding. Hence, considering that in these experiments, hnRNP L 162-321 or hnRNP L was overexpressed, together with the fact that the endogenous SETD2 abundance is low, it was not unexpected that the relative abundances between hnRNP L 162-321 or the full-length hnRNPL protein and SETD2 were sub-stoichiometric. Upon overexpressing a truncated form of SETD2 (SETD2C), which is stabilized as compared to the full-length SETD2 protein, and using that as bait, the dNSAF values were more comparable to what would be expected for a stoichiometric interaction.*
3. *Also, it has to be kept in mind that in these experiments, mass spectrometry identified multiple complexes that hnRNP L 162-321 or hnRNP L were involved in besides hnRNP L-SETD2 complex. Furthermore, hnRNPs are one of the most abundant and ubiquitous proteins present in the cells which makes it likely that they are present in vast excess over its respective binding sites. This, together with the RNA binding nature of hnRNPs, makes characterizing the interactome of the hnRNPs very challenging. In fact, the dNSAF*

of another established interactor of hnRNP L, Med23, was even lower than that of SETD2.

4. *Additionally, the size difference between the bait and prey proteins, 17 kDa for hnRNP L 162-321 or 64kDa for the full-length hnRNP L compared 282kDa for SETD2, could have had an impact on the purification of the larger protein with the smaller protein as bait after multiple washes during purification.*

3. Fig. 1. A Western blot with antibodies to SETD2 should complement Fig. 1d.

- *As per the reviewer's suggestion, a western blot using anti-SETD2 antibody has been added in Figure 1d of the revised manuscript. The very low abundance of the endogenous SETD2 combined with its high molecular weight (~288 kDa) makes it technically challenging to detect this protein* ***[Figure 16]****.*

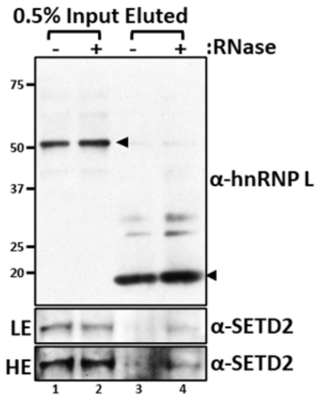

Figure 16. Halo purification was performed from extracts of 293T cells expressing Halo-hnRNP L 162-321. Input and eluted samples were resolved on gel followed by western blotting. The expected band for the target proteins are depicted by arrows.

4. Fig. 2c. The authors speculate that the abundance of hnRNPL interacting with SETD2C is so high that they see its band in the silver stained gel. However, the band they point as putative hnRNPL runs above the 58 kDa marker while in Fig. 1D we see that the apparent MW of hnRNPL 50 kDa. There are no detectable bands in the area of 50 kDa in Fig.2c. Again, a Western blot of the whole gel would solve the problem, that cannot be solved looking ast Fig. 2f because the authors only show cut stripes.

- *The expected molecular weight of the full-length hnRNP L protein is 64 kDa. Based on the numerous purifications we have performed using SETD2, its fragments, mutants as well as other proteins that do not interact with hnRNP L, we are very confident that the band marked as 'hnRNP L (?)' in Figure 2c is indeed hnRNP L.*
  In Figure 1d, the bands that are marked with arrows correspond to hnRNP L 162-321 (17.7 kDa) and not the full-length hnRNP L. The marked bands in lane 1 and 2 correspond to Halo-hnRNP L 162-321 (30 kDa Halo + 17.7 kDa hnRNP L 162-321 = ~ 50 kDa Halo + linker + hnRNP L 162-321) and those in lane 3 and 4 correspond to hnRNP L 162-321 (17.7 kDa).
  The authors would also like to point out that the anti-hnRNP L antibody (CST, 37562) used in the experiment is directed against a peptide in hnRNP L 162-321. Hence, the antibody should ideally detect both ectopically expressed Halo-hnRNP L 162-321 as well as endogenous hnRNP L in the inputs (lanes 1 and 2). However, with the loading of 0.5% input of that lysate, the endogenous hnRNP L protein (64 kDa) was not observed at that exposure level.

5. In any case, the dNSAF data shown in Fig. 2e seem to better support an interaction between SETD2C and hnRNPL (0.93 for the latter compared to 0.192 for the former). This confirms the weakness of Fig. 1 data.

- *In experiments of Figure 1, overexpressed hnRNP L was used as bait and endogenous SETD2, which is very less abundant, was the prey. That resulted in vastly different dNSAF values. Please see the response to comment 2. In this experiment, a stabilized form of SETD2 (SETD2C) was overexpressed and used as the bait. Endogenous hnRNP L is a highly abundant protein. The comparable abundances of the bait and the prey in these experiments resulted in more comparable dNSAFs. By performing the reciprocal purification using SETD2C as the bait and identifying hnRNP L as an interacting prey protein, we have validated the interaction.*

6. Fig. 3c. The authors only show mass-spec results to support binding of hnRNPL to SETD2 fragments 1964-2564 and 2164-2213. I would like to see the corresponding Western blots that would be complementary, using the same methodology, of results in Fig. 3g, where a SETD2C fragment lacking the 2114-2263 segment is nor able to interact with hnRNPL.

- *As per the reviewer's suggestion, we have added the corresponding western blot data for the SETD2 fragments 1404-2564, 1404-1963, 1964-2564, 2114-2163, 2164-2213, and 2214-2263 as part of Supplementary Figure 3 of the revised manuscript. The authors would like to point out that the anti-SETD2 antibody used in these experiments (Aviva, OAEB00589) is directed against the epitope ERDPDKQTQNKE which is present in the SETD2 fragments 1404-2564 and 1964-2564 but not in 1404-1963* ***[Figure 17]****. Also, the SETD2 bait fragments 2114-2163, 2164-2213, and 2214-2263 are only 50 amino acid peptides and hence, could not be resolved on an acrylamide gel.*

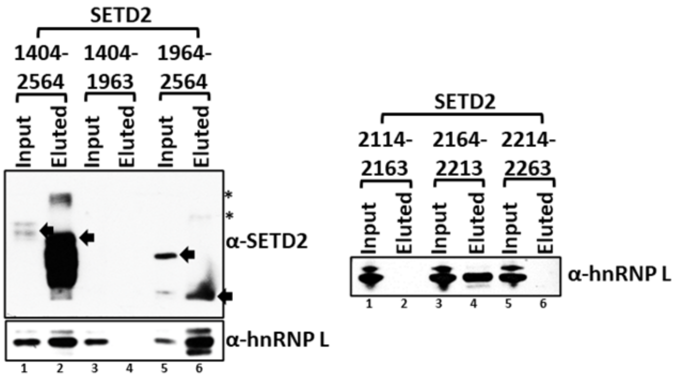

Figure 17. Halo purification was performed from extracts of 293T cells expressing Halo-SETD2 constructs. Input (0.5%) and eluted samples were resolved on a gel followed by western blotting. The expected band for the target proteins are depicted by arrows.

7. Figures 5a and b are very informative and conclusive to compare the effects of removing the segment of SETD2 that interacts with Pol II (SRI) or the segment that interacts with "hnRNPL" (SHI). I've already mentioned in "General considerations" my interpretation of SHI as a pan-RRM interacting domain rather than being specific for hnRNPL.

- *The authors agree with the reviewer's interpretation that the SHI domain of SETD2 might be involved in the interaction with other hnRNPs and splicing related proteins as well. Following are some examples from our initial submission to support this:*
    - *The title of our manuscript also reflects the same.*
    - *There is a section under Results with title 'Association of SETD2 with splicing-related proteins occurs through the SHI domain'.*
    - *In this section, we asked that 'Removal of the SHI domain from SETD2 leads to the loss of interaction with hnRNP L. We wondered whether the interaction of SETD2 with other splicing related proteins was also affected upon deletion of the*

SHI domain. To test this hypothesis, Halo-SETD2CΔSRI and Halo-SETD2CΔSHI, were affinity purified and subjected to mass spectrometry.’

- *We conclude the same section as follows: ‘Collectively, the analysis suggests that the SHI domain mediates contacts between SETD2 and proteins related to RNA processing.’*

As hnRNP L is the most abundant interactor in SETD2 purifications, we decided to investigate the SETD2-hnRNP L interaction in-depth and this is the story we are presenting in this manuscript.

The authors agree with the reviewer that our data summarized in Figure 5b raises the interesting possibility that besides hnRNP L, SETD2 can interact with other proteins through its SHI domain. To test this possibility, we purified SETD2 from mammalian cell extracts after depleting hnRNP L. hnRNP L is a very abundant protein in cells and robustly interacts with SETD2. Hence, despite achieving a significant depletion of hnRNP L in cell extracts, a substantial amount of hnRNP L was present in SETD2C purification (sihnRNP L) ***[Figure 1a, b]****. Despite this, we found that not only did the binding of SETD2 with other splicing related proteins persisted upon the depletion of hnRNP L, but also strikingly, their binding increased relative to the purifications from extracts of scramble-siRNA (siScramble) treated cells* ***[Figure 1b]****. This is what would be expected to happen if the other proteins also engage with SETD2 through its SHI domain where hnRNP L binds. Notably, the purification of SETD2 from extracts depleted of hnRNP L revealed enrichment of hnRNP A1. On the other hand, the enrichment of U2AF2 was reduced* ***[Figure 1b]****. Possibly, SETD2 can recruit spliceosomes by more than one mechanism. SETD2 by interacting with hnRNP L might result in the recruitment of proteins such as U2AF2. SETD2 might also engage the core hnRNP proteins which in turn can bring other splicing factors for the formation of the spliceosome to dictate the AS outcome. This data has been added as Supplementary Figure 6 and Supplementary Table 6 of the revised manuscript and appropriate discussion has been included.*

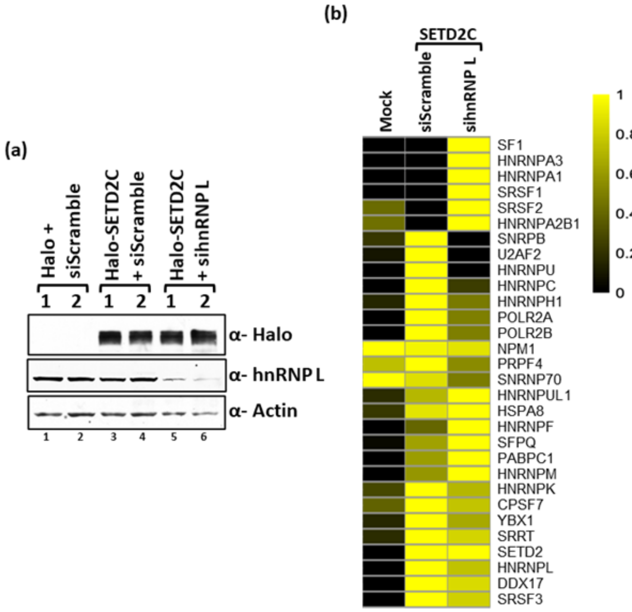

Figure 1. (a) Western blotting of whole cell extracts of 293T cells that were used for Halo purification followed by MudPIT. (b) Heat map showing the enrichment of proteins in MudPIT analysis.

In light of the new data that we have obtained and the reviewer’s interpretation of our data, we feel that the SETD2-hnRNP Interaction domain is a better-suited name for the novel domain that we have identified in SETD2, instead of SETD2-hnRNP L interaction domain. In future work, the exciting possibility of direct interaction of SETD2 with other RRM interacting proteins will be investigated in detail.

8. Lines 288-299: "The depletion of the targets at the protein level was confirmed by...." What is the figure or table that supports this assertion?

- *Figure 6a. This information has been added to the revised manuscript. The lower panel of Figure 6a confirms the depletion of hnRNP L by using the anti-hnRNP L antibody. The depletion of SETD2 resulted in a decrease in the global level of H3K36me3 as expected.*

9. Fig. 6. Commented in "general considerations".

- *The authors would like to thank the reviewer for pointing out our slip in not citing those pivotal papers. This has been rectified in the revised manuscript.*
- *In our manuscript, although we have focused on understanding the functional aspects of SETD2-hnRNP L interaction, we did not make an assertion that the SETD2 SHI domain only interacts with hnRNP L. In fact, the title of our manuscript and statements like lines 280-281 of our first submission clearly suggests that the authors believe that the SETD2 SHI domain might be involved in engaging proteins besides hnRNPL. As is true with most protein purifications, many proteins are co-purified with a given bait. Some are direct interactors; some are indirect, and many are contaminants. Especially considering that RNA binding proteins like the hnRNPs are common contaminants in most purifications from mammalian cell lysates, detailed characterization is needed to validate and demonstrate functional interactions. That is what we have done to not only prove that SETD2 and hnRNP L interact but also unravel the domains in both the proteins that are involved in the interaction.*
- *Although the Reinberg lab did report SETD2-hnRNP L interaction, they failed to characterize the specific domains in both SETD2 and hnRNP L that are responsible for their binding with one other. We not only demonstrate that SETD2 binds to RRM2 of hnRNP L but also identified a novel domain in SETD2 which is distinct from its known domains. These findings not only have the potential to have a broad impact on the study of hnRNPs but also undoubtedly will inspire future research on how the SHI domain regulates the function of the important methyltransferase SETD2. When considered together with the reviewer's and our interpretation that the SETD2 SHI domain might be involved in binding other proteins besides hnRNP L, the significance of our discovery becomes more pronounced. Furthermore, we also show that SETD2 and hnRNP L depletion results in overlapping splicing changes. As rightly pointed out by the reviewer, there might be functional redundancies caused due to the possible ability of SETD2 to engage other splicing factors besides hnRNP L. These possibilities will be investigated in the future.*

Text and cited references

Introduction

1. Lines 46-47: Splicing does not "determine the mRNA coding sequence" for two reasons: mRNAs contain 5' and 3' UTRs that do not code for protein and splicing also occurs in precursors of non-coding RNAs. I would replace "determine the mRNA coding sequence" by "yield mature RNAs".

- *The suggested change has been made in the revised manuscript.*

2. Reference 8 is not appropriate to illustrate coupling with splice site selection, i.e. AS, because it deals with budding yeast where AS is virtually not existing. For the kinetic model it would be better to cite either the original papers (de la Mata et al. Mol. Cell 2003; Dujardin et al. Mol.Cell 2014) or a review (Naftelberg et al. Annu. Rev. Biochem. 2015) from the Kornblihtt group.

- *The reviewer's point is well taken, however, the authors would like to point out that some of the key experiments performed to understand the coupling of transcription and splicing were performed in yeast as nicely summarized in the review by Oesterreich et al., 2011. As per the reviewer's suggestion, we have included the recommended papers as references in addition to the already cited paper by Schor et al. from the Kornblihtt group.*

3. Lines 66-67: Why cytokine stimulation dependent manner is mentioned here at the same epistemic level as cell type and developmental stage? One could argue that a general feature of AS could be "external cue dependent manner", which includes, apart from cytokines, every possible external signals like hormones, growth factors, temperature, UV irradiation, etc.

- *The reason why cytokine stimulation was specifically mentioned along with cell type and development stage is because AS is very well studied in these contexts. The effect of cytokine stimulation on AS in immune cells is an intense topic of research. Nevertheless, the reviewer's argument is well taken, and many other factors regulate AS. The sentence has been modified in the revised manuscript.*

4. Lines 72-73: "Combined with the fact that hnRNPs are very abundant and ubiquitous cellular proteins makes it unclear how the hnRNPs engage their target transcripts specifically". What is the subject of this sentence?

- *hnRNPs are the subject of this sentence. We have modified this sentence in the revised manuscript to avoid any possible confusion.*

5. Lines 82-83: Please replace "specific interaction" by either "specific interactions" or " a specific interaction".

- *We have replaced "specific interaction" with "specific interactions" in the revised manuscript.*

6. Results
Line 119: a reference is needed after the word "report".
Lines 172-173: "We were investigated which region..." The authors were not investigated.
Line 290: H3K36me3 methyltransferase should be replaced by H3K36 methyltransferase.
Line 407: A reference is needed after the word MRG15, which I suppose is Luco et al. Science 2010.

- *The authors would like to thank the reviewer for pointing out the needed corrections. The necessary changes have been made in the revised manuscript.*

Reviewer #1 (Remarks to the Author):

The revised manuscript adequately addresses the concerns and suggestions I had in my review. I notably appreciate the SET2 purification in HNRNP L knockdown conditions and the investigation of alternative splicing in SET2 knockdowns rescued with STE2 mutants. These experiments are considerably strengthening the manuscript.

Reviewer #3 (Remarks to the Author):

Although some of the experimental evidence is still weak, I recognize the effort to comply with most of my comments and criticisms. In particular I am happy that both in the title and in the text the authors broadened the scope at the interpretation of their results to splicing factors other than hnRNPL. The authors have also included missing references. However, they way in which they cite the two papers by Carmo Fonseca and Bentley (refs. 44 and 45 in the revised version, lines 366 and 367) is not appropriate. They say "This is consistent with previous reports that showed a strong positive correlation between splicing and H3K36me3 levels (44, 45)". These papers went much farther than just showing a correlation. By using both splicing inhibitors and removal of introns from template DNA, the authors showed that the act of splicing stimulates the deployment of the H3K36me3 mark. This is a cause-effect demonstration and not a mere correlation. So, I think the authors should replace their sentence by the following one: "This is consistent with previous reports that showed that splicing enhances recruitment of Sedt-2 and the deployment of the H3K36me3 mark (44, 45)". Until I do not see this sentence corrected, I am afraid that I will not be able to recommend publication.
Alberto Kornblihtt

NCOMMS-20-28264A

The histone methyltransferase SETD2 couples transcription and splicing by engaging pre-mRNA processing factors through its SHI domain

Saikat Bhattacharya, Michaella J. Levy, Ning Zhang, Hua Li, Laurence Florens, Michael P. Washburn and Jerry L. Workman

The authors would like to thank the reviewers for providing valuable feedback and suggestions to improve our manuscript, and the editors, for allowing us an opportunity to submit a revised version. The modified text has been highlighted in yellow in the revised manuscript.

REVIEWER COMMENTS

Reviewer #1 (Remarks to the Author):

The revised manuscript adequately addresses the concerns and suggestions I had in my review. I notably appreciate the SET2 purification in HNRNP L knockdown conditions and the investigation of alternative splicing in SET2 knockdowns rescued with STE2 mutants. These experiments are considerably strengthening the manuscript.

- *We would once again like to thank the reviewer for the constructive criticism of our work and suggestions that helped us improve the manuscript.*

Reviewer #3:

Although some of the experimental evidence is still weak, I recognize the effort to comply with most of my comments and criticisms. In particular I am happy that both in the title and in the text the authors broadened the scope at the interpretation of their results to splicing factors other than hnRNPL. The authors have also included missing references. However, they way in which they cite the two papers by Carmo Fonseca and Bentley (refs. 44 and 45 in the revised version, lines 366 and 367) is not appropriate. They say "This is consistent with previous reports that showed a strong positive correlation between splicing and H3K36me3 levels (44, 45)". These papers went much farther than just showing a correlation. By using both splicing inhibitors and removal of introns from template DNA, the authors showed that the act of splicing stimulates the deployment of the H3K36me3 mark. This is a cause-effect demonstration and not a mere correlation. So, I think the authors should replace
their sentence by the following one: "This is consistent with previous reports that showed that splicing enhances recruitment of Sedt-2 and the deployment of the H3K36me3 mark (44, 45)". Until I do not see this sentence corrected, I am afraid that I will not be able to recommend publication.

- *The authors once again would like to thank the reviewer for the constructive criticism of our manuscript for its improvement. We have made the changes in the text that were asked by the reviewer.*
